# Supplementary material for: “Fluctuation is the norm”: Rehabilitation practitioner perspectives on ambiguity and uncertainty in their work with persons in disordered states of consciousness after traumatic brain injury
Source: PLoS One. 2022 Apr 21;17(4):e0267194. doi: 10.1371/journal.pone.0267194 (PMC9022828; doi:10.1371/journal.pone.0267194)
Supplement: S1 File — (PDF) [file pone.0267194.s001.pdf]

Clinician Interview 1

Interviewer: AG

I = Interviewer
R = Respondent

I: OK, alright, so X like I said, we are conducting this study that is funded by the Department of Defense and the VA. And we're looking at patients who remain in disordered states of consciousness after their first year of injury. So part of this study is to better understand how clinicians and family members communicate changes of recovery that they have observed during the care that they provided. So today I want to talk to you about the care that you have provided for patients who were in that state so thinking about those patients who came to you in a vegetative or minimally conscious state when you started working with them. And I want to learn from your experience because we want to understand the language that clinicians use to communicate improvement, decline or maintenance, um and the language that you um use when you are treating those patients. So I am recording this interview. Any information that you share with me is going to be kept confidential. Any people or places that you identify during the interview will be removed in the transcription phase, and it won't be passed on through any analysis or dissemination. Um, so participation in this interview is voluntary. We've already talked about that. You've told me that you agree to participate in the interview, um, but if you at any time don't feel comfortable answering any of my questions or if you choose to end the interview you by all means can do that. OK?

R: Yep

I: Do you have any questions before we get started?

R: I do not.

I: OK, alright…so…first I just want you to think about some of the patients that you've treated in this population, and tell me about a time when you were surprised during your initial encounter with a patient.

R: Surprised about how they presented? Surprised about …what was reported? Anything?

I: Any of those things. Yep.

R: OK.

I: When you went in to see that patient for the first time, what you saw was suprising to you.

R: OK….um…I am thinking of one specific patient, who, he was a professional body builder. He was like over 300 pounds, but like muscular 300 pounds, but by the time I, he got to us as a patient he was down to about 180 pounds. I had spoken with the family prior to seeing him for clinical evaluation and family had told us all sorts of things that the patient was up to and doing and they were seeing, um, so I had one sort of image of what I thought I would see when I went into the room to do the assessment…um..however, when I got there the patient was not doing any of those things. Which will probably come up more, that's a pretty common, um…family thought he was speaking, or making attempts to speak, family thought that he was tracking, family thought that he was doing a lot of different behaviors that puts him a little further along in their recovery. Only for me, he was mostly giving generalized responses, um, not too much localization. Definitely no attempts at verbalization, command follow or tracking. Um so I was a little bit more surprised just given the information that I received um and also in the notes because I had done a chart review and a lot of the people had reported at his previous facility that

he was doing these things, when in fact he probably was not doing most of those things.

I: OK, so just to clarify, so can you give me an example of one concrete thing that the family told you that they….that you expected that patient to be doing when you walked in based on the….um…like describe something that you would have expected to see him doing

R: So, they were, they had said he was attempting to verbalize

I: OK

R: And um in the previous notes and so forth the clinician who had previously seen him said the same thing at the outside hospital. Only when I went in there he had that mouth movement it almost looked like a mouth munching um some movement around the lips and so forth that to an untrained eye I think would be misconstrued, I never heard any vocalization tied to it…umm…but they perceived this as his attempt to verbalize or communicate with us

I: OK…alright….so…when you went into that room and didn't see what you were expecting to..umm…how did you communicate that umm..that disconnect between what you saw and what the family was describing…how did you communicate that with the family?

R: OK, so, since it was only the first time that I had seen him, I was only in there for an hour ummm, I never discredit necessarily what they've reported or what we've seen because again I saw him at one moment in time and perhaps..I don't even remember what time..it was in the morning, but like you know maybe later in the day he was a lot more alert and was doing more or maybe I was an unfamiliar voice and person so he wasn't as comfortable doing the things that were reported..so when family did arrive later that day, umm..they asked how the evaluation went, did you

see him talking, did you see him doing all these different things…umm…I…tend to with this population wear more of my kid gloves as I call them, I tend to be a little more gentle in my approach so I explained to them you know during my assessment this morning I did not see that but we know with this population there is a lot of inconsistencies that we see, he might have better times in the morning or the afternoon or maybe with family around versus no family around so we're going to continue to assess. Every day I'm going to come in and see him we are going to try and find the time where you know we see him at his most alert ability able to participate in what we have to do..ummm. ..but at this point yeah I didn't see that this morning but if it happens again you know have the nurse come get me, I'd love to see it, that sort of thing. Now after awhile we got to a point where we had to have a discussion that what we were seeing, and I described to them was that mouth munching is an involuntary movement and I don't think it's him actually trying to talk, he got to a point where we could occlude the trach to see if there was any vocalization or anything and he couldn't get that. So eventually we started to have more of that tougher conversation that's not necessarily a purposeful movement or behavior that we're seeing.

I: So….as he…progressed through rehab were there any other things that during that stay surprised you at all? Did he umm…make any changes that you can describe?

R: He, I know that at one point he was started on a neurostimulant, and I can't remember which one at this point in time but I know that he definitely, we were giving the DOCS at the time he did make some minor changes in his scores but during his entire time with us we never did see him emerge to consciousness..he never or even…I think if I am remembering correctly he bordered minimally conscious but we never go to the point of consistency with either verbalizations, command follow, tracking, anything of that sort….umm….but definitely had a little pick up when they started the neurostimulant.

I: OK…So describe to me….think about..um..when you've been trying to get him to follow a command can you describe what you saw? So describe the command to me and then describe what you would see when you observed him.

R: OK..um…so…we tried a variety of different commands..umm…that were within his motoric system um..so I know with OT and so forth they had worked on things that were more functional like can he bring the washcloth to his face, so I knew that they were doing that and with hand over hand they were having him do that so I tried things, he had movement within his upper extremeties so we tried things like, can you bring the comb to your hair, can you brush your hair or comb your hair for me. Can you wipe your face? He did have some saliva secretion he couldn't manage it, so can you wipe your face for me…ummm..we also did try tracking asking him to follow ..umm…can you look at yourself in the mirror? Ok now I am going to move the mirror can you follow the mirror with me…ummm…sometimes we would see a little bit of movement initiated with that upper extremity when you would give him an object but he couldn't fully bring it up…..my understanding with the OT and PT involved it wasn't that because he motorically couldn't do it it's just that he….it almost would question if there was an apraxic component because he couldn't get that up there on his own.

I: Ok…alright…so he couldn't move…

R: He couldn't follow the command to do it even when placed in his hand

I: OK…alright…ok…so I am going to have you move on to a different patient….ok? Now I want you to tell me about….um…one of your most memorable experiences treating a patient with severe TBI. And it doesn't necessarily have to be a patient that remained in a minimally conscious or vegetative state the entire time but tell me about the most memorable experience you have treating a patient like this.

R: OK…um…probably like one of my top three patients of all time (laughs)umm.. Her name was xx, she was a 19 year old college student..she was driving…she was

in the what do you call it the ummm army reserves and she was going to school and she worked full time at McDonald's and she came to us in a vegetative state…she..her mom reported that at the acute hospital they had basically told her to give up on her daughter, put her in a nursing home, forget about her, she's never going to be able to do anything and to hear that is just disheartening and then especially when you hear her whole story it's really disheartening but, so she came in vegetative state…ummm..we did the DOCS…she started to emerge and then probably within a couple of weeks she was admitted to what was our AMICOUS program at the time..ummm… she started to emerge to consciousness and then just sort of took off from there….ummm…..we saw much more you know, command follow, ability to functionally use objects, her ability to communicate her basic wants and needs, um, she ended up being severely dysarthric, so communication was a big struggle for her….but….she just did exceptionally well and ultimately ended up going through our whole inpatient program, our day rehab program and outpatient and she ended up graduating from college with honors, got her Master's degree, lives independently and just had an amazing outcome…ummm….and this is someone who early on they said just give up on her. So I know her family really, every year her mom parades her through the emergency room that she went through just to show like don't give up on people you never know what could happen or what their outcome could be

I: Yeah, that's awesome. That's great. Ok, so, I'm gonna, I'm gonna have you go back now to when you first saw her and so when you first met her and she was in the vegetative state…describe to me what you saw.

R: Sure. So she um….when I first evaluated her and early in her stay she was not command following, she was having intermittent between generalized and localized responses to things…if I'm remembering correctly because it's been a few years now, um, I know that we got the most when it came to localization to tactile early on with her…umm.. she was really sensitive, like mom had done her internet homework, like she had googled things at the time and found things so they had

already started doing almost like a sensory stimulation program with her when she was in acute care before she came to us and maybe that's why she was, most of what she was doing was tactile. Ummm….and then she sort of started to emerge, we started to see much more localized responses, her start to attempt to do some yes and no, it wasn't always consistent but she would make a she would attempt to answer the question…umm… and then…progressed onward from there

I: Awesome. Ok, now I am going to take you back to the ..um..the tactile..the response to the tactile stimulation..so when you say that she started out with generalized responses to tactile stimulation describe to me something….like give me an example of what you saw.

R: Sure, so I know ummm….She was really sensitive, I remember the nose, well you wanted generalized, I remember…ummm….ice, cause we would do the DOCS so she would have ice on her toe…ummm….we would see changes in heart rate, respiratory sort of changes we didn't ever see til later…then she started to take off we'd see her actually try to kick me when we put the ice on her foot

I: So later she would do that?

R: Later

I: So when you started off you would see changes in her heart rate and respiratory rate and you saw that because you were monitoring that

R: yeah, I had a pulse ox on her finger

I: Ok and so then later you said when you'd put the ice on her toe

R: She would try to kick me

I: She would try to kick you

R: (laughs) One early on though when she localized all the time was the feather to the nose for some reason she was….that was her thing…from like day one she would score two on that I remember

I: Ok, alright, um….OK, so then you said as she progressed she started to do more localized responses and she was attempting yes no responses…so describe that to me…tell me…give me some examples.

R: Um, so I have to think back…..(knock on the door – interview stopped for a moment)….Ok, so ummm….she would start to….so she would start to…we had the yes/no cards..ummm.. she would start to look more at the yes and no when a question was asked, they weren't always correct but she would make an attempt to answer the question by shifting her eye gaze to yes or no…ummm…in regards to localizing responses I remember that she, like I said she would kick her foot for the cold, I remember. we never really saw too much with smell…umm….with her…but in regards to..umm…visualization she started to do more tracking…umm….so she would localize to where ever we were…ummm…same with sound, if we would play a sound she would turn to find that sound and or for I know when her name was called all of a sudden, before or early on she might have a generalized or no response but then (sigh) I don't know, maybe two weeks in I mean pretty close, you'd call her name and she'd consistently look for you. You would do…ummm….the bell, she would look for you

I: So when you say that she would look for you, would she physically do something?

R: She'd physically turn her head.

I: Turn her head?

R: She'd physically turn her head.

I: OK, alright, so she was making all of these awesome changes. Tell me about how you were communicating this information as a team, so when you would meet with your team members how did you communicate those changes to them?

R: Ok, so we would report the scores because we were doing the DOCS weekly…umm… and I don't remember but she scored X one week now she's scoring Y..umm…we would also talk about what were the changes we were seeing specifically so last week when we called her name there would be no response or maybe you know heart rate changes or something…something more generalized and now I'm calling her name and she's turning her head to find me…umm…you know the same thing with the toe and ice, things of that nature, like in our huddle as to what we were seeing different in behavior this week versus the previous weeks

I: OK and then how 'bout with the family, how would you communicate those changes to the family?

R: OK, so….mom was there primarily and how I'd explain it to mom is….we're presenting all this different stimuli to xx, this is all in her environment and so forth…when..I…put the ice on her toe and we see a change in respiratory rate, for example, she knows something is going on but she can't pin point what it is…her brain's saying "hey wait a minute that's not right why is my toe cold" but she didn't know at that point how to respond appropriately, but as she continues to heal and as her brain is healing and we're trying different medications..um…we start to see, now I put the ice on her toe and she's like wait a minute my foot's cold, I don't want it there, I'm going to do whatever I can to get away from that or kick the ice away from my foot. So I always describe it as your brain is healing and as your brain continues to heal you start to be able to do a little bit more and progress towards that…ummm…..now she's staring to be able to have the ability to communicate with

us in some way, again, higher level thinking, your brain is continuing to heal, we're moving towards more of a state of consciousness and a further progression in her healing.

I: Ok…so tell me…Ok…I am going to take you back again. So the communication, you said that when she first started communicating when you were using the yes/no cards and she would look to yes/no but she wouldn't always be consistent.

R: Correct.

I: Ok…so…can you describe to me…ummm…at the time that she was discharged what that communication looked like?

R: So she emerged to full consciousness, so when she discharged she was verbalizing all of her wants, needs and ideas everything. She was pretty dysarthric so she did have basic AAC in place for like if people couldn't understand her message…family was great, I mean they got it probably 80-90% of the time but if you had an unfamiliar listener they definitely struggled more so she had a basic board and so forth in place so if she needed it but otherwise she was able to verbalize everything she needed or wanted

I: OK…and I understand what you're telling me but I am going to ask you to go another step further….So tell me what she would use the basic board for…what are some things she would communicate with it. How would she use it?

R: So she had just a basic laminated sheet…it was an alphabet board so she could spell out a word…she also then on the back of it had your basic things like hungry, thirsty, bathroom, so let's say a new nurse came in and she said "I have to go to the bathroom" but the nurse couldn't understand because she was so dysarthric she could use her board to point to bathroom. Oh, you need to use the bathroom, and then the nurse could take her to the restroom.

I: Awesome, OK. Is there anything else that you can think of about her that you want to share with me?

R: I don’t think so. I mean, these are the kind of stories that everyone hopes. That their loved one is going to have this outcome. And some do and some don’t and we don’t understand why some will and some won’t. But it is a pretty great story. A pretty great recovery.

I: So, is there anything at all about her when you first started working with her that made you think that she would have this kind of recovery?

R: Did I think she would do well, yes. Did I think the outcome that we end up with five years later would happen, I didn’t know. But I knew she had a lot of things working for her early on. She was not too far post injury, it was maybe 3 weeks or a month, it was not very far post. She had an amazing family and support system. Mom was there every day and if mom couldn’t be someone else was there. She was young. She was healthy previously. She was going to school. She had all of those things that we know bode well for people working for her. So, we knew that if someone is going to have a good outcome, she was going to be one of those people likely.

I: So based on, not just this history that she had, when you first saw her what about her made you think that she had… what kinds of behaviors did you notice or what made you think that she might have a better outcome than someone else?

R: I can’t say specifically anything to be honest with you. Just on her assessments or her initial DOCS or evaluations or anything? I don’t think there was anything glaring from that concrete kind of objective data, you know, her score was really high or her score was really low. It was somewhere in the middle if I am

remembering correctly she didn't one way or another. But all those other things kind of contributed to it.

I: So nothing about her behavior? So there isn't something that you could describe about her that… like just a feeling that you got and if …
R: Not specifically. Sorry.

I: No, no that's okay. Okay, I am going to have you think about one more patient. This time I want you to think about a person that you treated that you felt stuck with.

R: Hm. Okay. There was a young man. He was, I don't know, 16 or 17 years old. He was away at like a military boarding school and he went to a party on a weekend. And he overdosed on something and ended up with a hypoxic injury. Is it okay to talk about hypoxic ones?

I: Yes that is okay.

R: He was down for a really long time before they were able to resuscitate him. Um, he was not doing very much when he was admitted the first time. He was very long post getting to us and we know that hypoxic injuries don't tend to have a rate of prognosis as traumatic injury. But he even got to us it was even 3 months post. He had one infection, one thing after another when he was in acute care. He came to us for rehab and literally over the course of the next 6 months was in and out of our system because of infections and he just medically wasn't stable. He would go out for one infection, come back a week or two and something else would happen. It was like… the doctor just kept bringing him back even though there was no change every time. He was just too medically complex at that point because we were always dealing with those issues on top of the fact that he wasn't yet conscious. We pushed back a lot and would ask "what is the point of this admission, what was the purpose, what are we going to achieve with this young man"? Especially when it got to be that he was 9 or 10 months post, what are we accomplishing, you know? And the

plan was always he was going to go to a skilled facility, mom couldn't take care of him at home. We trained mom to do basic things, but beyond that what were we accomplishing by continuously bringing this guy back through rehab. So, we did feel a little stuck.

I: Okay. So, thinking about treating him, tell me what he looked like when you walked into the room. Describe to me some of the things. What could he do or what couldn't he do?

R: So, he… When we went in, I could remember one of the first times, he trach pegged was on a high humidity trach collar at all times. He was not able to manage his own secretions which were eventually one of his big downfalls in regards to going in and out constantly. He didn't tolerate upright, so he was almost always flat or fairly flat, I mean they could get the head of the bed up for tube feeding obviously but getting him up in a chair was almost always a challenge because we would see a change in respiotory status, change in heartrate, he would become diaphoretic. So we were almost always having to work in bed. He had already established contractures and so forth from a physical standpoint because he had been in a hospital for so long without proper care and management of those things.

I: I am going to stop you for just a second. This is really good information but when you said that he didn't tolerate upright you're saying that he was not able to tolerate sitting up in a chair?

R: Yeah, a tilt and space type chair is typically what is issued to a patient such as this young man. He almost always, I mean we could tilt him back in it and he would do okay but any time you tried to get him as close to 90 degrees as possible you would see physical changes. And then nursing would get nervous and put him right back to bed. He didn't fare well on the, I don't remember the scores obviously, but I don't think he had any localized responses because there was no command followed, no visual tracking, anything of that nature. Everything was either no response or I think

he had some generalized, obviously, but nothing beyond that. And his scored never really changed much over the course of different admissions with us.

I: So can you think of one example of a generalized response that he gave you? Can you describe that?

R: Um. I am trying to think, I can't think of anything specific. At this point I can't think, I'm blank.

I: Okay. So, just in general then if you think about him presenting in a kind of generalized responsive way, how would you describe that?

R: He never was never able to pin point exactly what our target was. So, going back to the previous if you were to put something cold on his toe he never tried to move his toe away or kick his foot away. And it wasn't necessarily motoric in nature that he couldn't do it because he could you'd see him moving in bed and so forth, he had the motoric capability. He never tracked in anyway, he never focused on anything. At one point we had… we were suspecting, "could he be blind"? because no matter what we never saw anything visual with him…so.

I: So when you worked with him visually, give me an example of something that you would show him and what you saw when you would show him

R: Okay, so his room was covered in, obviously, pictures, cards, everything else. Family brought a ton of pictures in so we would use… we tried objects, we had tried different things that way, but we would try pictures of like him and his brothers and so forth. And presenting it in front of him, he would look forward at it but it wasn't necessarily that he was looking at the picture. It was just that he would stare forward no matter what. And then if you started to move the picture either right to left or up and down his eyes never moved with that picture.

I: So, tell me how as a therapist when you are looking a patient when you are presenting a picture to them and you feel like they are staring but not at it. Can you describe that to me, how do you know? How do you know they are not looking at it?

R: Because it seemed, at this case at least, it seemed that his visual was always straight ahead. No matter if there was someone in front of him or someone wasn't. His eyes never moved a lot with whatever was in front of him, whether it was a picture, a person, an object. It was just like this straight ahead kind of gaze.

I: Okay.

R: And then if I did put something in front of him that gaze never shifted or changed and I would be like, "okay I am going to move it can your follow it"? And he couldn't. Even if I gave much more direction like "I'm moving it to the right, I'm moving it up", it's till just a straight ahead stare.

I: Okay. Alright. Thank you, that's helpful. So then tell me about this feeling of being stuck because he had medical complications, because he was back to you a lot, how did you navigate that from a team perspective?

R: So it was definitely a challenge for the team because it was pretty much all the therapists and nursing against this physician at the time. The physician was like "we got to give him a change because he is a young guy" and all of us were like "we've given him lots of chances and nothing is changing and his outcome is going to be the same. We are not going to see, you know, a change. I mean, the first couple times we were accepting of him coming back, this guy literally it was 6 or 7 times that he was in and out.

I: So was the physician seeing something you all weren't or why do you think…?

R: I think the physician at the time she was young, nothing against young physicians, but she had just finished residency, she was just finished a fellowship, was still relatively new to being an attending. Hadn't had a lot of experience in her residency as well as her fellowship with this population. And I think… She was that way with all of her patients, but I think this guy had a special place in her heart for whatever reason, I'm not sure for this patient versus another one, we all have our reasons. She wanted to give him every single opportunity and never give up on that chance that maybe this admission would be different.

I: So, as you kind of navigated this, and you came in and out, what did that experience look like at the end when he was finally discharged? Is there any… can you describe anything that had changed since the…?

R: So essentially he never got an official discharge if you will, he had gotten sent out for whatever number of time that was, 7 or something, and then insurance refused to bring him back because they, also, were like, what are you doing for this young man, you brought him in for the past 6 months, we bring him in for a week or two and he ends up in acute care and maybe he is just too fragile and not stable enough to tolerate what you guys have to provide. We never got that official, kind of, parting of ways were, you know, we had to compare it to because he got sent out emergently.

I: Okay. So, was the family involved in this situation?

R: Somewhat. Father had passed away; mother came a couple times a week. Brother was the one that was the closest but he also was in school and working and so forth. Intermittently throughout the week you would see different people but it was never someone sitting bedside 24/7.

I: So, how did you navigate that communication of that being stuck, with the family? With everything going on, obviously it seems that there was a lot going on back and forth. First he was here and then he wasn't.

R: So we handled… We never let the family know that we were felling stuck essentially. They didn't know that. But they even at one point would make joke like, "here he goes again, we will see you in a week or two". Brother was pretty realistic as too, he was highly educated but also kept a foot, with like he read that his anoxic injury in this case and he probably doesn't, and it's been nine months, so he kind of understood a little better than mom who was "this is my son, of course we are going to do everything we can until the end of time." Which I understand as a mother I get that, you would want to exhaust every effort we have. But we would not let them feel that way, we did our same thing we trained them. They were able to do a sensory type program with him, do his transfers. They learned how to manage his splints and so forth for his contractures. This protocol stayed the same with them, but we would, obviously they would ask me "what happened with his scores this week? Oh, isn't today the day you are going to do the DOCS, what does he get?" And I would say, you know, we didn't see much change. He was someone they did try a couple of different things with stimulant wise, but since he was so fragile we had to be careful what we could give him and how much. So, we didn't have that advantage to see.

I: Can you think of an example of how described that to them, when you didn't see a change from one week to the next. Can you describe that to me? Can you explain that?

R: Um, I would say "Last week we gave the DOCS…"
When I was giving it in inpatient I tried to give it with regularity, unless we found a reason to give it at a different time, I tried to always make… you know whatever…Thursdays at ten o'clock I would see the patient so we would have that consistency versus one day it would be the morning one day the afternoon. Obviously if they try to medication and family says "Hey I want you to try it in the afternoon" we were flexible with it. But I remember we would just go through and say ok last week when we administered this, these are the responses. I would keep

the sheet and if they wanted to see the sheet obviously I would show them, “here he wasn’t responding, this week he also didn’t respond to the stimuli. These days he responded, last week he did but this week he didn’t, so maybe he is more tired this week or maybe other stuff was going on.” We could also gage if he was getting sick because he would start to trend downward and you would see a drop off of score. But the family started to see that too clinically even without the scores. “He’s not having a good day today, I do think something is brewing, and sure enough within a day or two out he went.

I: So, can you think of a specific example of something that he did that would tell you that something was brewing and he was on his way out again?

R: He wouldn’t open his eyes as much; his eyes would be closed, so I wouldn’t even get that stare. We would be fighting to open his eyes up and it would be like, “Do I need to tape your eyes open today”? He would be sweating, he would be diaphoretic. Sometimes he would start to have a low grade temp. But he definitely wouldn’t perform…even though his scores weren’t super high to begin with he would score lower when things were going on.

I: So, he was someone who wasn’t responding even to very much tactile right?

R: Yes

I: So, can you give me an example of something that he was doing on a good day versus on one of those days when you knew he was going downhill?

R: Without looking at the forms I can’t think of something specific. I think it was more just his clinical presentation. You would walk in and he would be dripping sweat or the bed would be soaked. Or his eyes would be closed; the eyes were one of the biggest telling signs because he would have his eyes open, not that we

questioned if he could see but he was always very eyes open when you would walk in but if something was going on they would be closed.

I: Okay, alright. All of those descriptions are helpful. Is there anything else that you can think of that, with this population, as we talked through some of these situations that you would want to tell me that I didn't ask you? As far as communicating either to families or your team as far as change goes?

R: I think it is really hard for newer clinicians to work with this population. It is hard for everyone, I'm not going to lie, even with years of experience it's a challenging population and especially when you work on a unit where you have a variety of, levels of change, I guess. I have a rancho of 2s and a range of 8 all on the same floor, right. Or you have a rancho 4 or whatever. So, you have those families of the lower level patients who want… "I wish my husband was kicking, screaming, and swearing and everything." So, it is really challenging when you have that mixed population and it is definitely for a newer clinician to feel comfortable and it is hard if they don't make change to be like "So, they got the same thing" or "we are not seeing them start to track the way we hoped they would" or "we tried this medication" and for some reason this group it hurts more almost. It feels worst to give those "he didn't make a lot of progress reports" then it does to someone who maybe is a rancho 5. Yeah, they are still disorientated. Yeah, they are still in posttraumatic amnesia. Ok, we will keep at it versus someone here where there on that borderline of success and they just haven't been able to get over that hump. So, I think it is definitely a much more challenging group to work with. And I don't think that every clinician has the skill set, necessarily, to work with this population.

I: So tell me what that skill set is.

R: I think it is the ability… not that I'm perfect by any means but definitely I think that there are some clinicians that have a better ability to communicate with the families about change or lack thereof versus those that do not. Or who understand

this population better and there are a lot of clinicians still, sadly, that don't think these are rehab candidates that think "what are you doing in this bed, you're not conscious, you can't follow commands, you can't do these things, why are we working with you at this point. If you are going to regain consciousness you are going to do it no matter what." There is not a ton of research that says what we are doing makes them come to consciousness, right, so it a hard sell for certain people and those are the people that I think have a harder time having those conversations with family.

I: So, what do you think the ideal communication situation is? Or how do… can you describe that?

R: I think you need to, as I said earlier with my "kid gloves", I think this population you have to start out a little more docile then you would with any other family. You gage, I mean certain families can take news better than others, you gage obviously. But I think that they have been through the ringer so much and because they know, at least in our program, they are there for a limited amount of time. Now it is not as limited as it use to be, early on it was like you got two weeks or you got three weeks and that was it. And if you don't make change then you are not going to continue here. So there was that pressure on them and I think being able to explain you know this is a process, if it is going to happen we are going to take our time and do everything we can to get them there but ultimately the brain is healing and everyone's brain heals at a different time and feeling comfortable to have those conversations.

I: Okay, it makes sense. Alright, I think that's it. You shared a lot of good information.

Clinician Interview
Interview # 2

Interviewer: EM

I = Interviewer
R = Respondent

I: We are conducting this study that is funded by the Department of Defense and the Veteran Administration for patients who remain in disordered states of consciousness during their first year of injury. Part of this study is to better understand how clinicians and family members or caregivers communicate changes they have observed in this patient population. Today I want to talk to you about the care that you have provided for patients who were in that state of disordered consciousness, meaning they were considered to be in a coma, vegetative state, or minimally conscious state. I want to learn from your experience because we want to understand the language that clinicians use to communicate improvement, decline or maintenance, and plateau in neurobehavioral performance when treating this population. This interview is being recorder for research purposes. The recording will only be shared with authorized members of the research team. Information you share will be kept confidential. Any people or places identified during the interview will be removed in the transcription phase, and it won't be passed on through any analysis or dissemination of this study. Participation in this interview is completely voluntary and you may choose to end your participation at any time. If you choose to end the interview any information already collected, via audio recording can be used for analysis. Do we have your consent to continue?

R: Yes.

I: Okay. So, I want you to go ahead and think about this disordered consciousness population and patients that you treated and I want you to tell me about a time when you had a frustrating experience.

R: Hm, um. I think one of the first minimally conscious or comatose patients that I ever worked with was when I was a new therapist and I really didn't know what to what to expect with someone at this level. I wasn't expecting a patient like that to get admitted to this type of setting. So, it was frustrating for me to figure out what I was going to do with the patient every day in therapy when they are not responding to anything. They weren't demonstrating any balance reactions; they had very low tone everywhere. For their entire stay they were a passive participant. So, we are used to patients being an active participant in therapy. So, trying to figure out how to set my goals accordingly and patient due to extenuated circumstances ended up being here for months. And so, kind of doing the same thing every day with him due to limited options we could work on tolerance to an upright position, range of motion, and on the rare occasion that his mom, who was his primary caregiver, was actually here I was able to do some caregiver training with her. But otherwise it was the same thing every day working on tolerance to upright, siting balance, head control, and range of motion. And this went on probably for a couple months before they finally found a place where they could be discharged to.

I: You had mentioned that the patient had low tone, can you explain to me what that looks like?

R: So, his limbs and his trunk and his neck were all flaccid. I wasn't seeing muscle activation anywhere.

I: And you said that he wasn't responding, what kinds of things were you trying?

R: Um, sitting balance trying to get him to initiate some balance reaction or protective reactions from an unsupported sitting position. Um, I would try to get him sometimes on the motomed lower extremity odometer to try to provide some proprioceptive input to his legs to see if he would initiate ligament movement. Um, and he never did, he never came around unfortunately, his injury was so severe that I don't know if anyone expected him to make progress, but, you know, we had him so we had to keep working with him, keep trying.

I: So when you were working on the balance, and you said you were trying to work on balance reactions what kinds of things were you looking for?

R: Ym, I was looking for him to initial some type of either activation of cervical muscles or head control, or activation of his core muscles, his abdominals or his trunk extensor, something to try to keep himself from falling, to protect himself or to prevent a loss of balance.

I: And did you ever see any response from that?

R: No. Not in that particular patient

I: So, when you are trying to illicit these balance reactions, can you describe for me what that would look like?

R: So, um, generally what I do is I try to get a patient starting from an upright, neutral sitting position or a I'm sitting on the mat table behind the patient supporting their trunk and I withdrawal my support to allow them to loss their balance and see if they react at all, see if they initiate. Sometime I might start with them from a forward bent position and withdrawal my assistance and see if they will try to go from a forward bent position to extend their back and sit up or vice versa I may have them sit up and let go and see if they initiate some abdominal

control to lean forward. Or you can have them leaning to one side or the other and let go and see if they initiate some control in the opposite direction.

I: So, going back, you said this patient didn't make any progress at all. So, how did you communicate that with the rest of the team, the fact that you weren't seeing any progress?

R: Well, in this setting we have daily huddles where we talk about every patient and we talk about progress and once a week we have to write a progress note and we go into more depth about the progress the patient has made. So, I would communicate what I'm seeing to the physicians and the other team members as far as if they were meeting goals and what their current limitations still are.

I: So, what were this patient's limitations, do you remember? Can you give me an example of what you would say his limitations were?

R: Uh, his altered state of consciousness was his biggest limitation, the general hypertonicity, the lack of motor control.

I: How did you know that he was in an altered state of consciousness?

R: Um because he wasn't responding to any external stimuli. He didn't respond to noxious stimuli.

I: What do you mean by noxious?

R: Painful or uncomfortable stimuli. Sometimes I might try to get him to react by doing an external rub or, maybe a rubbing his chin or tickling his face or pressing my fingers into his spine to get him to initiate some type of extension. He never had any response to that.

I: No reactions?

R: No reactions to that. No reactions to loud noises. No reactions to visual threat. He wasn't visually tracking objects or people.

I: Is there anything else about this patient that you would like to share?

R: No. (11:10)

I: So let's move on. Think about your most rewarding experience treating a patient with disordered consciousness.

R: Um, one of the most rewarding experiences I had is I had a patient come in that was…I guess he would have been considered a rancho 3 on admission. So, he was minimally conscious showing signs of inconsistent arousal and attention. He was moving spontaneously not necessarily purposefully or on command. I think the initial evaluation he was not showing any balance or protective reactions and unsupported sitting. He had a lot of cervical flexion tone, so his head was stuck in a flexed position. So even if I was to force him in an upright position I could never really get him to an upright neutral cervical position. Um. But we kept working on it every day when he first got here. Initially that was sort of our focus of therapy was getting him to initiate some head and trunk control. He was a young patient and over, probably, the course of three or four weeks I noticed that his initiations of balance reactions and trunk control and head control started to slowly improve and he reached a point where he was slowly able to sit up by himself or with very little assistance. And he just kept making more and more improvements. He started moving his limbs more purposefully and he stated to help with transfers. He started to initiate some leg extension during transfers to help with weight bearing and eventually he was initiating lower extremity extension and truck extension that he came to where we started to try some standing. He was very dependent on standing initially with probably a total assist

of two people to get him on his feet. But every day he just kept requiring less and less assists for everything and then we started doing some body with supportive gait training. He made very good progress with that then we started doing over ground gait training with two people doing handheld assist. He was really, really challenging because we started to become more and more conscious he went from the rancho 3 stage to more of a rancho 4 stage where he was confused and agitated and uncooperative and sometimes combative and sometimes resistant. But, we persisted in therapy tried to walk him as much as we could. His parents were very involved; they would help and actually ended up walking out of here at a min-assist level. From where he stated that was a pretty rewarding case because of how far he came. He went from nonambulatory, total assist for everything, to min-assist by discharge.

I: That's great. I am going to rewind a little bit back to when you said he was initially showing inconsistent arousal and attention. Can you describe for me what that was like.

R: So there was times were he basically appeared to be asleep during therapy where he would not be responsive to verbal commands. He wouldn't open his eyes, was not necessary responding to noxious stimuli and then there would be times where he was quite awake where he would be visually tracking, inconsistently following commands.

I: What kind of commands?

R: Like, lift your head up or put your hand on the mat to try to support yourself. Sometimes you could command him to look a certain direction and he would look a certain direction but at that stage he was not consistent yet.

I: Okay. And then has he stated to make that transition, you said from the rancho 3 to the rancho 4, how did you know that that was starting?

R: Um, he started to follow commands more consistently, his arousal was more consistent, he wouldn't switch from a state of wakefulness to a state of being asleep within a span of a single session. His movements became more purposeful where he would purposefully reach for and use objects.

I: Can you give me an example of that?

R: For example, when he was sitting at the edge of the mat, he would put his hand at the edge of the mat to support himself, whereas before he was not doing that. He might try to purposefully try to grab you and hurt you or pinch you, he is a pincher. Like for bed mobility, moving from side to side, he was purposefully using the bed rails to try to pull himself over onto his side. He was able to start holding on the parallel bars.

I: I can't remember if you said he had family involved?

R: Yeah, he did, his parents were very involved.

I: And how where you communicating some of these changes to them?

R: Um, He was a TBI from a… he was a pedestrian that got hit by a car at high speed. So, with his type of brain injury, he has a diffuse axonal injury, so with that type of injury the progressions that he was going through were normal and what we would expect to see. So, I always tried to communicate with his parents that these behaviors that we are seeing and the progression that he's going through is normal progression from someone who is recovering from a TBI. So, I would always try to point out to his parents that these things we were seeing and some of these behaviors, although sometimes they seem negative because he is going from being somewhat sedate to somewhat agitated it's easy to interpret as a negative thing, but we interpret that as a positive thing because it means he is going

through the progression of things as we would hope to see for somebody with that type of injury.

I: Can you explain what diffuse axonal injury is?

R: I can try. So, diffuse axonal injury is a way of describing a sheering injury in the brain where the axon or white matter are sheered basically you have axonal damage throughout the brain or in multiple areas of the brain. It is usually associated with a high speed accident where the whole brain is jarred within the confines of the skull. So, the grey matter on the outer part of the brain moves at a high speed relative to the white matter which connects to the brainstem and those axons get sheered. So, they refer to that as diffuse axonal injury and with that type of injury you often expect to see the brain sort of recover from inside out, so you start to see recovery function from the midbrain out towards the cortex.

I: So, that inside out recovery that you are talking about, what would that look like to somebody looking at this person from the outside?

R: Um. So, that inside out recovery is sort of described by the different rancho levels. So, early on as somebody is recovering, rancho 2, rancho 3 stages here see recovery of the mid-brain and as they progress to rancho 4 you are starting to see more recover of the cortex part of the brain.

I: So what does that look like behaviorally with the patient?

R: Oh, behaviorally? So, behaviorally as a progress from rancho 3 to 4 you are seeing sort of non-purposeful, maybe some motor restlessness to more pronounced motor restlessness, more purposeful behaviors. You are stating to see the patient verbalize more and you are starting to see the patient more agitated, not necessarily aggression but agitated behavior which can be motor restlessness, perseveration… um.

I: Can you explain what perseveration is?

R: Perseveration is when you get stuck on a certain thought or idea and you're not able to redirect your thoughts. So, being focused on having to go to the bathroom all the time, for example, or being focused on having to call your family member or just some idea that you just can't stop thinking about.

I: Ok. And one more, can you describe for me what motor restlessness looks like?

R: So, motor restlessness looks like, I guess a good laymen's term would be someone who is fidgety who can't sit still. They may be consistently shifting around in the wheelchair or sliding out of the wheelchair or constantly rolling from side to side in the bed or trying to sit up in bed or lay back down. They have a hard time staying in one place. And it is usually not due to any external stimulus they just are going all the time it doesn't have anything to do with someone bothering them.

I: I know that we kind of got off the patient that we started with, so I am going to bring you back to him. You said when he had moved to rancho 3 to 4, that he was confused. Could you tell me what that looked like?

R: So by confused I mean disoriented to time, place, and situation. He didn't know where he was or why he was here. Didn't know what time of year it was.

I: And eventually he moved out of that?

R: Yeah. Eventually he… when he left here I would say he was more of a rancho 5 at the time he did get discharged. Still not fully oriented, still confused, but not agitated. Not internally agitated. He could still be externally agitated, and by that I

mean, if he was agitated it was usually because of something or somebody did, or some type of external stimulus that bothered him.

I: Anything else about this patient that you want to share or I didn't ask?

R: No

I: No? Okay. I want you to tell me about a time when you were surprised during your initial encounter with a patient with this sort of state of consciousness.

R: Hm. Well this may not necessarily be what you're looking for but we have had a number of patients that on paper are described as being minimally conscious but by the time we see them for their initial evaluation they are actually conscious. We often get those types of surprises and I don't know if that is because whoever said that they were minimally conscious doesn't understand what minimally conscious is or maybe somehow in the transition of whenever that was written to the time they got to us maybe they emerged to become conscious. But I feel like that seems to happen a lot.

I: So, when you get that chart and it says they are minimally conscious, what are you expecting to see?

R: Um. I am expecting to see, possibly, somebody that is inconsistently awake and not awake, maybe inconsistently verbalizing, inconsistently moving, not necessarily purposefully and somebody that is not able to express your needs.

I:Did you ever have the opposite occur where you had a chart that somebody was doing XYZ and you went in and they weren't doing what the chart said?

R: I can't think of a time off the top of my head where somebody was significantly worse than they said on paper, at least not in terms of consciousness. I have had patients come in slowed on medication but they were given something

to sedate them, but that is not the same as a different level of consciousness than they were described.

I: So, let’s think of another patient of disorder consciousness who maybe wasn’t surprising at the initial encounter but maybe down the road in their stay something surprised you about them.

R: Um. We get a lot of patients, especially some of the younger ones with described disorder consciousness, that end up emerging fairly quickly and end up doing better than anyone could really expect, but that is the thing about disorders of consciousness that are heard to predict how they are going to recover because a lot of times that initial evaluation they are just not giving you anything. And so because you never really know what to expect it is always sort of a surprise when they do recover because we shouldn’t be surprised but we often are pleasantly surprised. I think more so with the younger patients, but um, I have had some older patients too that have surprised me that end up doing a lot better than you could hope for.

I: Let’s pick maybe one of the older patients; tell me what they looked like when you first started seeing them.

R: Let me try to think of one. Um. I’m trying to think of specific examples. Ok, so, sorry can you repeat the question?

I: Talk about somebody…

R: Who came in unconscious who…

I: Ends up surprising you at some point. Not necessarily at the beginning.

R: I had one patient who I can't remember exactly how old he was, I want to say he was in his late fifties, who came in a rancho 3, I think he had had a motorcycle accident and he had a history of chronic alcohol abuse which is often a poor prognostic indicator especially in older patients. So, I honestly was not that hopeful that he was going to make a great recovery

I: Just based on…

R: Based on his age, based on the severity of his injury and the history of his alcohol abuse. It is often a pretty poor combo but motor-wise he ended up doing fairly well. It took a few weeks before we started to see him become more conscious and interactive but once he did he kind of took off motor-wise. I don't think he was ever orientated to his time, place, or situation while he was here, so cognitive his recovery was not that great but physically he ended up surprising me because I think we ultimately ended up getting him walking at contact garter min-assist level. He still had a lot of behavioral deficits, he had no idea where he was but. So that is one example of someone that surprised me.

I: You said he took off motor-wise, what does that look like.

R: When he first got here he was not really initiating a lot of movement, he was pretty dependent for transfers and mobility, and he wasn't helping with anything. Once he started moving more purposefully, he… sometimes when they first get here and they are not moving, you are not sure if it is because of paralysis or they just are not initiating. In his case it was just that he wasn't initiating, he was not paralyzed anywhere. He was actually pretty strong and so once he became conscious and moving purposefully he was able to start using his strength that he still had pretty quickly. And he progressed from being nonambulatory to ambulatory within a span of a week or week and a half.

I: And then you mentioned that even though he was walking pretty independently, contact guard, he still had behavioral deficits. Can you explain that to me?

R: Yes. He was probably still a rancho 5 by the time he left, so he could still be difficult to engage in therapies because he was still disoriented to situation, I don't think he ever really understood where he was or why he was here so trying to engage him in therapy could be hard. He didn't have enough insight to be motivated to participate; he didn't recognize his own deficits. He didn't recognize the need for therapy and he could at times be combative if you would try to push or coerce him into doing therapy.

I: Okay. Anything else about this patient?

R: Nope.

I: Nope? Okay. We can do one more. Can you tell me about a time when you felt stuck with a disordered consciousness patient?

R: Um, stunk in terms of progress, or…?

I: Yeah. Progress, discharge plan, therapy plan, family…

R: Um, I know that the first patient that we talked about, the one that was I guess comatose or rancho 2 through his entire stay. I definitely felt kind of stuck with him both due to lack of progress or lack of a viable discharge plan. He ended up being here way longer then he needed to be just because there was no place to send him, his family couldn't take care of him and he required so much care that most facilities didn't want to take him either. So, it took us longer to find him placement. I think he ended up in a long-term care facility.

I: And he never made any changed while he was here?

R: He never made any changes while he was here, no.

I: Okay, anything I didn’t ask you that you want to share about your experiences?

R: No, not that I can think of.

I: Anything else you would like to comment on about disordered consciousness population?

R: Not that I can think of right now.

I: Okay, thank you.

Clinician Interview 3

Discipline:

I – Interviewer

R – Respondent

I: We're conducting a study funded by the Department of Defense and the Veterans Administration for patients who remain in a disordered state of consciousness during their first year after injury. Part of the study is to better understand how clinicians and family members or caregivers communicate changes they observe in this patient population. Today I want to ask you about the care you have provided for patients who are in a state of disordered consciousness, meaning they were considered to be in a coma, a vegetative state or minimally conscious state. I want to learn from your experiences because we want to understand the language clinicians use to communicate improvement, decline or maintenance and plateau in neurobehavioral performance when treating this patient population. This interview is being recorded for research purposes. The recording will only be shared with authorized members of the research team. Information you share will be kept confidential, and people or places identified in the interview will be removed during transcription and not passed on to the analysis or dissemination of the study. Participation in this interview is completely voluntary and you may choose to end your participation at any time. If you choose to end the interview, any information already collected via audio recording can be used for analysis. Do we have your consent to continue?

R: Yes.

I: Ok. So, I want you to think about these patients in the disordered state of consciousness that you've worked with and I want you to tell me about your most memorable experience treating one of these patients.

R: Uh, the most memorable one was probably one of my first patients here, because I haven't been here for a year yet, umm. Uh, we did the DOCS on him, Disorders of Consciousness Scale,

and um, … I think at first umm, uh… I don't know where I'm going with this. Can you ask the question again?

I: Sure, um, tell me about your most memorable experience.

R: Oh, Ok. So I think he was so memorable because the fa.., he was my first patient and because the family was so involved and they had a lot of questions, so I had to like study up really hard and understand his predicament and how to explain to the family what is going on. Um, I think what was also memorable was that I worked with him for 3 months, so he kind of just, he made a lot of gains but he never really…he did, he did leave the minimally conscious state but because I worked with him for so long, that's why it was so memorable.

I: Ok. Can you describe him for me? What did he look like when you first started working with him?

R: Umm, when he was first here he was giving us mainly generalized responses, ummm, we'd give him different stimulation, whether it be taste, sound, calling his name, giving a mirror to look at, and quickly he had more localized responses, um to where I could say his name and he would look over, or his mom would ask him something he'd kind of respond. Umm, and then eventually um, in conjunction with um, continued therapy, medication and with the brain healing, he was becoming a little more responsive, umm, but it was just little things, like his mom would ask him to give her a kiss and he would give him a little one, umm, I'd ask him to do something and if I'd get him at the right time of day when he'd just had medication, he'd be able to participate a little bit, move very slowly, he would need time to respond, umm, and I think a lot of times that his parents would interpret his umm, reactions to his personality. So, if like, I said or did something and then he would respond to me in a certain way then his parents would be like, "Oh, he's he wants this, or he's doing this because of this, but it's, sometimes it's hard to…you're always putting yourself into the patient's shoes and kind of being the voice from them and it might not be what they're exactly communicating. Or they might not be communicating anything at all.

I: You've given me a lot of really good stuff, so we're just going to go back and

R: Oh, sorry,

I: No, that's good, you've given me really great stuff. Umm, so you said at the beginning he was giving you generalized responses to different stimuli

R: Umhm, umhm

I: So can you give me some specific examples of stimuli you used

R: Yeah

I: And the responses you saw.

R: Yeah, so umm, when he was first here we used the Disorders of Consciousness Scale, so that's a series of different stimuli, you do to a patient to see how they responded. So there is either no response, a generalized response, so if I say your name um and you kind of just don't look at me, but you make, your eyes will flutter, you'll open your eyes, make a movement, that's a generalized response. Umm, or I would put a mirror in front of him to look at and or any patient, and how do they respond to that mirror, they can look at it, do they track the movement of it, um, if I hold out a colorful ball and ask them to follow a command "Reach out and grab the ball" do they actually reach out and grab it, do they look at it, which is generalized, reaching out and grabbing it would be localized, or do they do nothing. Another one we do is taste, so we take a cotton swab and we put it in some juice and we put it in their mouth to see do they lick their lips, or do they like ha… grimace or anything, or do they just not respond or do anything.

I: So you said he started out first with the generalized responses…

R: Yeah,

I: …and then moved to the localized responses umm, and then you also mentioned that the family um seemed to be misinterpreting some responses?

R: I think so, I think a lot of times what happens with patients is they're just doing generalized responses or it's just a movement and the family is like "Oh, he's in pain" or "Oh he wants something" or "Oh he needs something" and it like I don't know how to communicate to the family that you know, they might just be making movement, but I would never tell them that because that takes away, like hope, or anything and that's the person's loved one, so I just kind of go with it, like "yeah, you could be right, definitely"

I: Do you remember like a specific response that was misinterpreted for this patient, a specific example?

R: Umm, let me think, not for this patient, there's another patient and umm she has umm flexor tone, so

I: umhm

R: …anytime you mover her she just kind of like curls up, because just the tone and her parents think like "Oh, she doesn't like it, she's like curling away from you" and it's like " no, that's just like a tone response, it's not really anything" but I wouldn't say that, I'd be like "maybe, yeah"

I: So how do you, so then uh, talk to me about how you navigate that with the family.

R: Uh, I try to give a lot of education in terms of what's happening when their brain is healing and as they are going through therapy, umm, about the, we talk a lot about the Rancho scale, and I kind of give them an education on that I what like, that kind of helps them understand cognitively how the brain is healing um and then I talk to them about any other symptoms that patients have, like the tone, or umm, or anything else, just continued education.

I: Ok, so um, I think you said to at the beginning this patient emerged from disordered consciousness.

R: He did, yeah,

I: Ok, so how did you know that he was emerging?

R: So, I think the biggest clue was when um, I was talking to speech and they had a video of him actually singing along to a song and then his responses became more consistent more localized, he's following commands, he was participating in therapy, he was um (chuckle), one of my favorite memories of him, was I was pushing him and I was like get, like asking him to do a task, I was really pushing, working really hard, he looked at me and he mouthed "You suck" and I was like "yay! You're talking"

I: Umhm

R: So

I: Only a therapist would appreciate that!

R: Yeah,

I: So how important is it that communication with your allied health counterparts? When you are working with these patients?

R: Oh, very important! Sometimes they see something that I didn't see, so like, with a person who's minimally conscious, when they are out of it, they need to have one of four things, command follow, eye tracking, um, functional communication or functional object use. Once they do it once, then they are not in minimally conscious anymore, so then if it doesn't happen during my like thirty minute session I can talk to speech and be like "Yeah they were following,

they followed a command" like "What really?!" so communication is key, I'm always asking the other therapists how that patient is doing or progressing.

I: Uh, anything else about this patient that you want to share with me or that I didn't ask?

R:

I: No? Ok, ok, so let's go on and let's talk about a frustrating experience that you may have had with a severe TBI patient.

R: Umhm, oh yeah, so I think of the frustrating experiences when and it's and it's no one's fault, umm, one patient we just kind of, we had hopes for, umm, the doctor was saying to all of us, "He's going to get better, he's going to come out of this" and then he never did and it was just getting frustrating because I was, his family had really great insurance so they kept extending his stay but we were just kind of doing the same things over and over again and ummm, and I knew this patient was probably uncomfortable just like I'd have him like tall kneeling, or sitting at the edge of the mat and it's like not very comfortable. And then we find out later he's not going to make progress, they took him to the brain scan and they realized the damage was a lot more significant than they originally thought. So it was just frustrating that I like worked so long with this patient, I got really close with his family and then we get that news like it's not getting any better and it's just kind of going to be who he is. Maybe not forever, but for awhile there was not much else we could do.

I: So how did that conversation go with the family?

R: Umm

I: As far as his treatment.

R: Umm, so it's kind of funny cause a lot of times we rely on the doctors to have those difficult conversations but then sometimes, depending on the doctor, we've got great doctors on this

floor, they are team players and they will have those conversations, umm, but other time the p…the families grow close to us because we see them everyday for an hour or two or…and we're spending a lot of time with them, so they feel more comfortable coming to us. Even though the doctor says one thing they will come to us and be like, "what do you think?" and I kind of have to determine what the doctor says. I never want to extinguish hope, but I want to keep people realistic.

I: So what kinds of things did you say to this family?

R: Umm, I would say things like "Hey, like this is how he is now, so we don't know where he's going to be one, two, three, four, five, six months from now, a year from now, so we need to focus on where he is now, and how he's going to be able to live and be as independent or as functional as possible at home. So then instead of talking more of the recovery model, I switched gears to more of um, the um, how, how he's gonna…what's the word I'm looking for, um, compensation, so how are we, how are we going to home, what tools do you need, to like make sure he can shower, and get dressed, he can go to the bathroom, all that stuff. So, the conversation switched from "He's going to get better and be able to do this," to "Ok, he's not going to be able to do this right now, how is he going to go home and be taken care of."

I: Can you describe him for me?

R: Umm, he's in his early twenties, umm…. I don't know, how much I can say because of HIPPA.

I: No, uh, more like presenting

R: Oh, ok

I: what was his clinical picture, what was his neurobehavioral condition?

R: Oh, ok, umm, he had a lot of, he had a lot of tone, extensor tone in his hips, in his legs in his knees, he moved very slowly, umm, he…I don't know how to describe it, he was like this basically, umm

I: So kind of stuck?

R: Yeah, stuck pretty much, kinda like…not locked in, but kind of locked in. I, and this might be me projecting on him, I feel like he's in there, like his personality was there, he just wasn't able to move his move his body and express his feelings as much as he wanted to. That was after, when he was out of the minimally conscious state.

I: So how did you know that?

R: Umm, I think it was just those responses he would have, umm, when, cause sometimes he could mouth words

I: Umhm

R: When he told me I suck, when he was able to mouth words to songs, or follow commands consistently.

I: Ok, so I wanna um go back to what you said about you could, you could tell that he was there.

R: Umhm

I: So, as best as you can…

R: Ok,

I: Because I know, I know what that's like,

R: Yeah.

I: Can you put that into words.

R: Yeah, umm, when, I guess I can compare it to someone who, who I worked with who I think wasn't quite there yet.

I: Ummhmm

R: to him, so the other person she, if I said her name, if I tried to catch eye contact with her, if I tried to get her to track something, there was just not really consistent response. She would either look at me, look away, look around, close her eyes, like hard to arouse when I gave her the sternal rub she would kind of keep her eyes closed. This other patient, he, I, he was there, I would call his name and he would look for me, or when I was talking to him he would lock eyes on me and like, you know like, normal conversation with a person you make eye contact, you nod, when they're talking, kind of acknowledge what they're saying, he would do that like maybe little nods, or he'd look at me, or he'd be like we're going to do something hard today, he'd sigh, and then like "ok, here we go!"

I: Perfect.

R: Does that

I: Yeah, yeah yeah, that's great. I know it's a hard thing to try and put into words

R: Yeah, like you know it when you see it.

I: uh huh, yeah. Umm, anything else about this patient?

R: No

I: Ok, Tell me about a time when you were surprised with an initial encounter with a disordered consciousness patient.

R: Mmmm, I don’t think I’ve ever been surprised, yeah, I don’t know, I don’t have an answer to that one.

I: No,

R: No

I: So something like, um like uh you walked in expecting one thing and you got something else, or

R: Oh, Yeah! OK, yeah. I guess this happens all the time when we get new admits and they’ll they’re minimally conscious or AMICOUS and we had a girl and I walk in and she’s talking and she could kind of sit up at the edge of the bed and she could put on a t-shirt, so I guess that was a time where I was the most surprised, where I kind of expected her to be more, I was expecting to do the DOCS on her but she was like….I asked her her favorite color and she was like pink or what’s your favorite animal? Cats….so

I: OK, or something like um…um, like the opposite of the last patient you talked about, where you said you know the doctors said he’s going to make progress and then you know he’s not, anything the opposite where you were told somebody’s not going to make any progress and then all of a sudden.

R: No

I: No? OK. How ‘bout a time when you felt stuck with a patient?

R: It would be the same guy, we, I just kept trying the same thing in treatment over and over again, with, I mean, there was that first initial like span of improvement and then he just kept

getting extended and extended and extended and I was like nothing's changing. Only if he had medication like right before my session then I was like going to get something out of him, but other than that he was just like, sometimes I just felt like I don't know what to do, or like, and I think that he doesn't want to be here anymore and he wants to go home, and I felt stuck like we shouldn't keep him here because like medically he's doing fine he's just not progressing through his other therapies. And I think we all kind of felt stuck.

I: Ok. Can you give me a comparison of what he looked like without the medication versus with the medication?

R: So, without the medication he was not very responsive um his eyes his eyes were open, and, he'd look at you but he wouldn't move his arms or his legs, his balance, we would sit him up at the edge of the mat, his balance was not there, um, he wouldn't mouth words, he wouldn't nod or shake his head or say yes, mouth yes or no. um. And then I think the parents, their big indicator of when the medication was working was when they asked him to give them a kiss and he gave them a kiss like then they knew the medication was working. And then when the medication was on he'd be able to hold himself up at the edge of the mat he'd be able to move his arms a little better and participate in therapy, he'd make more facial expressions, and he'd mouth more words.

I: OK, um so what kinds of……now I'm going to back up…..did you use the DOCS with this patient?

R: Yeah.

I: And, how did you use that to inform your treatment plan?

R: ummm, knowing that so we use the DOCS and we even though speech kind of reports RANCHO level, we all collaborate on that and the DOCS is like huge for us to understand where that patient is so knowing that OK like right now we're rating him as a 2 with generalized

response I'm still going to look for that localized response. So it's like I'm using the DOCS to inform me what to look for when I am working with that patient.

I: Can you give me an example?

R: umm, just more command follow, more eye tracking, um, functional object use, or just consistent responses to different stimuli……just looking at it and maybe showing a different facial expression

I: Um, what kinds of stimuli do you use? As far as like what you're giving people to look at.

R: Oh, um, it kind of I really guess it depends on the person, so this particular person for him, according to his family he's always loved looking in the mirror at himself and when I got him a mirror he like locked on to it and he would follow it and another patient we have she looks at umm people's eyes and if she catches your eyes she'll follow them, she'll track them. Other patients they use pictures of family or video or maybe music and they turn to it. Ummm, I'm not sure if it's premorbidly what that patient preferred to preferred, I like to kind of think that, but ummm it all, sometimes it just varies on the person.

I: Anything else about these patients that I haven't already asked?

R: Ummhmm.
I: Alright, let's try one more. Tell me about a rewarding experience. Your most rewarding experience.

R: My most rewarding experience. ……..ummmm……OK, it's like, it's the same story, when I was pushing that patient and he didn't want to do it but he did it anyway and he looked at me and he said "You suck" and I thought, I got teary eyed because I was like that's the first time he like communicated , like mouthed words to me other than yes or no or just gave me a sigh and he made like a grimace face with it and his mom was like "Wow, he would never say that". And I

was like no he said it. I offered, I don't know for some reason it was very rewarding knowing like that I was pushing him to the brink where he would mouth words like that. I'll take it.

I: Yeah. Umm. So what were you having him do when he said that?

R: Ummm, we were, I was having him move his arms, so I would put his arms in overhead pulleys, so for lack of a better term he kinds of looks like a marionette doll. But we do that so we can unweight his arms so that he is able to produce shoulder movement. Umm, without his forearm and his hand weighing it down. So I put him in a pulley and I was asking him to punch the ball, punch the ball and I kept rolling the ball and he wouldn't do it and I kept saying you have to do this you have to do this, I kept pushing and pushing and I was just really annoying basically, and he just, I think he just had enough of it and he just looked at me said (whisper) "you suck", and then when I told his mom he said that, he like looked at her with these big bunny, funny big eyes, little puppy dog eyes, like "I would never do that", I guess that's me projecting again, but….

I: Umm, so how did you know that you could push him?

R: Ummm, well his, I kind of used family as my guideline, so he's a professional athlete, um, and he said that he wakes up in the morning, he hits the gym like three times a day he loves to be pushed, he loves to push himself. Umm, and at first when I worked with him, I pushed him a little bit but not too hard and I, after awhile I kind of felt like I was babying him, so then I kind of did more challenging things. I would, get him to do tall kneeling on the mat which is not comfortable and for me to him up in there, it's a lot of work on my end or get him in the stander and have him do a functional task, ummm, which that's a lot. I guess I could have just kept him in his chair and kept working on arms and hands but I was like I think this person according to the family wants to be pushed, even though, he's uncomfortable, so I kind of used that as my guide.

I: So you've talked a lot about all of these patients, you've talked about the family influence, um, so how much does that play into these patients recovery?

R: A lot, ummm, I think the more the family is involved the better the patient outcome, in terms of not just the patient recovery but when they have to go home and take care of that patient themselves. Some families are fantastic, they're here everyday, they're here during therapy, they're asking all the questions, they want all the information. Some families are too much, they really come on strong and they provide too much stimulation, to where the patient is just like overwhelmed and then you have to do like, you have to ask them to take a step back. More education about knocking down over stimulation um, and then the majority of the time families get it. But, I think the more the family is involved the better they feel prepared for the patient. I think they also get more realistic expectation about recovery umm and I think it's just better for the patient with family there

I: Can you describe over stimulation?

R: Umm, yes, so it is when, it is when, well, I'll just give the family example. It is when they are just constantly like "come on do it, let's go", so "you can do this, come on look up", "look up, sit up straight, do this do that" (said quickly and stearnly). Like that giving them constant directions of what to do. And it's like let's just give them one thing at a time to focus on. I am having them sit up at the edge of the mat, they don't need to sit up perfectly straight each time while I'm working on trunk control and sitting balance, like they can take a break, they can do it slowly, the families just like come on come on come on come on (with a quick tone)….do this do this do this I know you want to do this (spoken quickly again). It's like stop, calm down.

I: So umm, how do you communicate that with the family? The patient's needs and what you're trying to do in therapy with this expectation that I have to constantly be giving cues?

R: Yeah, so, I always tell the family, like, I am doing this because I am working on this, I'm getting them on the edge of the mat because I'm working on trunk control. I'm putting their arm in this pulley to see if I can get them to move it. Um, I'm playing music because I want to see if this patient will look over at it. Ummm, and then when I do something and someone's like, and they start overstimulating I kind of take a break and give them the education piece. If we give

them too much stimulus it’s too much for them to be able to do anything at all. So we should just take a step back and focus on one thing at a time.

I: Anything else that you wanted to share about this patient?

R: No.

I: No? OK, so I want to ask you specifically about your use of the DOCS. Um, with this patient population, um, how often you’re giving it, what you’re using it for, how you explain it to the family?

R: Yeah….umm…kind of what I’ve already said, about what I’m looking for when I’m giving the different types of stimulation. Are they going to give me any response? A general response or a localized response? We do that, we do it when the patient first gets here so we have our baseline and then depending on whether the patient emerges from a minimally conscious state, we might do it again week after week, umm, I don’t think I’ve done it more than two, three times on a patient. A lot of times, well, I do it on my own usually, ummm, I think we do have patient here that has been getting it a couple times. But, yeah, I haven’t done it too many times on one person.

I: how do you know when you want to stop using it?

R: When they emerge.

I: When they emerge?

R: Yeah

I: Ok…. Ummm, and can you tell me how you know, give me some specific examples um of emerging behavior.

R: Umm, just the, like the four things, tracking, command follow, functional object use and functional communication. One of those and them I'm like, and then, they say that they only need to be doing it once but if it's once and then I don't see it again, then I kind of keep working with the DOCS but then if I see it consistently then I'm like OK they're here.

I: Umm, what kind of functional object use, can you give me an example?

R: Ummm, yeah, so, one patient gave him a toothbrush and they tried, they like held on to it and they did like the brushing teeth motion or gave them a washcloth and they're able to bring it to their face and they wipe their face as best they can.

I: Ok. Anything else about the population, you want to share anything I haven't asked you?

R: Ummhmm.

I: No? OK. We are all set

Clinician Interview 4

Interviewer: EW

I = Interviewer

R = Respondent

I: So I want you to think about the patients in the disordered state of consciousness that you've worked with……..I want you to tell me about your most Memorable experience with one of these patients.

R: Um, I think working with this patient population, I've found they….it's the connection not only with the patients but also with their family members um… I should have like prepped for this……I think that I think about those patients that respond to something that I wasn't expecting and those are some times the most memorable experiences, so you know I think about um this one patient that I had I think two years ago and he was in like slumped over position and it was really hard to tell kind of like what he was responding to or if he was responding to anything and those little things that you do like I was just trying to get him to respond to anything functional object use or something

I: Can you give me an example?

R: Um, yep, so I took the basketball and I tossed it in his lap and like nothing happened and it kind of rolled away to his wife and so I sat behind him and I was working with him on the mat and tossed the ball again into his lap and like one arm kind of started to kind of jolt over and then the ball rolled off his lap so then his wife tossed the ball again to his lap and like one kind of hand came and caught the ball a little bit. It was really inconsistent at first but then like it was funny to see how as he progressed out of the disorders of consciousness state, how, those initial

responses became more and more consistent and I think one of the things that I find I tell family members a lot initially is that um we're looking for patterns that become more consistent so like the……..so the um one time that I see um them open their eyes to a favorite song, um or the one time I see them lock to my eyes when I'm talking to them or the one time that I see them um you know grab the washcloth or um take the picture or something then I tell the family members we want the consistency and I think the things I love the most is when you see somebody, you see somebody start to be more consistent. So they grab it once in a week and you don't see it again and then all of sudden they grab it twice and you see them grab it a little bit more um like I'm working with a young woman right now and she has no functional use of her hands meaning that she's not grabbing for things, she's not reaching for things, her hands are pretty contracted I'm not really seeing anything there. She has pretty poor head control, pretty poor trunk control, she's not able to hold her head up when she's sitting and yet if you say her name (Is it ok if I say her name), so if I say xxxx, right? She…..if I'm within her view so she doesn't really have to turn her head…she like locks eyes. And those are the times, that, like, and again at RIC we are typically, I would say for the most part get Rancho 2s, Rancho 3s, rarely are we getting Rancho 1s, I can think of only time that I worked with a Rancho 1. Um, but even that Rancho 2 state when we are seeing mainly generalized responses so meaning that sort of their bodies are just overall responding to a stimulus versus a loc you know consistently responding to, it's a hard thing not to use those terms, so, um, this young woman she's not responding to tactile stimuli so much but she will lock onto auditory so she'll lock onto your eyes and those are the time that you know I think it is really interesting to see, as a clinician that is motivating. Like the one time that I think of that we had a guy that was like a Rancho 1, I mean, it was really hard you just didn't get anything. No reciprocal response at all, positive or negative. Those are the more challenging types.

I: So you are talking about Rancho 2 and Rancho 3 can you describe those a little bit for me?

R: Rancho 2, for me, is somebody who is responding in general to things. So, like the young women I was just refereeing to, if I roll her around in bed she is not moving her arm to like stop herself or she is not reaching across to assist herself but her eyes get huge, her breathing rate

increases. If I had a pulse ox on her heart rate is probably increasing because she is getting stimulated and her body is responding to that in a generalized way. So her body has an all response to it as opposed to, as I was talking about earlier, as opposed to her reaching across and grabbing something with her hand. That one I would call a generalized response, so if I said 'xxx we are going to roll' and she kind of reached her arm a little bit or she help initiate roll that would be more of a localized response. The same thing for her and for other individuals, right when I'm touching them or turning on music they may respond overall, so they might open their eyes, they may move their arms a little bit but they are not looking at the music or looking towards the music or looking at the phone or that kind of thing. That's more of a generalized one. Localized response is something were I would say, well like the young one who I was talking about where she, like, locks on to my eyes. She is showing that she is responding to my voice not just a voice randomly happening, she's knowing that… she is able to specifically identify that it is me talking to her and she is giving me that sort of reciprocal response.

I: So how do you differentiate them between a Rancho 2 and a Rancho 3?

R: Um.

I: Is it the generalized or localized?

R: Clinically?

I: Yeah

R: Clinically, I am going to look at, I mean we do use the disorders of consciousness scale and we also use the CRS. We I say collectively because the speech therapists are using the CRS and we use the DOCS. And so with the DOCS I would say there is not really… we are not using the CRS or the DOCS as a cutoff point because, that would be an interesting research project, but I

don't think that there is, to my knowledge that the is a correlation between a DOCS score of this equals Rancho. And I don't think it is meant to be that way. So, I don't typically use a DOCS score or a CRS score to give me that indication. I would say that I focus more on localized, generalized response to kind of choose the Rancho 2 and 3. And it is a little bit of a discussion because as an OT I think I'm lucky, versus a speech therapist who may only be seeing them from the bed level. I can get them aroused and awake so that I can get them able to respond in a different way. Whereas, for a bed level or chair level I think speech often only see them in a certain way. And the reason I being that up is because as a team we talk about it and I'll say no I am seeing a localized response, they are localizing to this… and speech may say I see that but I'm not seeing it consistently. So that is why sometimes we will wait until it consistent or cross disciplines before we jump between levels

I: Perfect. That's great, that is exactly what we are looking for. So, I want you to try and focus on one patient. Tell me about your most rewarding experience.

R: Um, it's tricky because a lot of the rewarding experience part is more of watching them comes out of the disordered conscious state.

I: Mhm, that's fine.

R: Um, but I'm trying to think of someone who stayed in that state a little bit longer than expected. So, I think one of the most rewarding parts of the process for me, is watching an intervention that I… So, I was trained by my colleagues to just try stuff. Because the research… there is a lack of research, not because people aren't trying, just that there is a lack of research with disorders of consciousness state as far as interventions that actually work. A lot of the times I feel like we are trying stuff, and we are just seeing what happens. And I think the most rewarding part of working with different patients is trying something and seeing it work. And when I saw seeing it work, that could mean a lot of different things. So, for example, one women

that I am thinking of, she was… her muscle tone… so the way she was positioned in the sort of tightness over all in her arms and legs, was really hard to manage. It was really challenging for her mom to toilet her, for bed level to change her. She looked really uncomfortable overall. It was just really hard. And I for her, the biggest thing we were trying to figure out was how to keep her as comfortable as possible so that we could get another response out of her. And so, when it comes to trying something and seeing if it works (those were air quotes there) a lot of it was trying to find a position for her that was most comfortable. So, some of the things that were most reward is finding some random positions, whether it be side-lying or prone. I use prone a lot, prone just means on your stomach. I use a wedge to give them some support so their head doesn't hang down and if they have a trach they aren't bumping all over those res concerns. But someone who once you get them in prone and their balance system is sort of all over the place, and three minutes go by and they would fall asleep. She would fall asleep in prone which just mean to me that we finally found a position for her that was comfortable and her body could just (sigh) take a big sigh and relax. And I think that for that young women, her mom was really motivated to get her in that position, which therapeutically, is also just a great positions from a nursing and a medical and therapeutic perspective in general and then to have that carry over to where it was something that would benefit her. Then there were some other times when we got that same young woman into random positions that she didn't love very much but she responded to in a way which showed she was using muscles in a way she may not have been able to before. For example, we got her into tall kneeling which means she was just on her knees and I was supporting her from behind. We had a big ball or bolsters in front of her that her mom would push against. So we were effective… I was holding her from behind, we were using the bolster to hold her up from the front and having her lean forward and kind of her work on her postural muscles and then to hold her head up. She did not like it very much but she was definitely using her muscles in a lot of different ways. So, I think that having that sort of… in a way call and response in a lot of nontraditional ways, so instead of me saying 'hey, how's it going' and they respond, that's a traditional response that I would love, but a nontraditional response would be like I put someone in a position and they respond or I move them around and they respond or I play music and they respond… so, ways that I would not normally expect to interact with patients.

I also think, something that is rewarding in general is watching people come out of a disorder state of consciousness. I can think of many people, the young man with the basketball for example he was slow goer in terms of coming out of a minimally conscious state and he did and it was still slow even when he did, he never moved fast, that wasn't his M.O. Watching him take steady steps to come out, you know. One day he would grab a washcloth and the next day it was he finally pushed his arm through the shirt and it was little victories that we got every single day, and I think those are the most rewarding experiences that I can think about. And those are far more numerous than I give credit to because I usually see people for 4 to 8 weeks I usually don't remember those first ten days. I mean, I do, but I often remember what they were doing by the time they left. Like, I still remember this one guy who got to us and wasn't responding to anything. I literally shave him for an hour trying to get him to wake up, trying to get him to smell things, and shave his face, and water, and didn't respond and was asleep the whole time. One of my coworkers had two minimally conscious patients on her caseload and I said 'hey let me take one' to kind of mix things up a little bit. And the one that I took, this guy that I shaved ended up like really making strong progress to come out of a minimally conscious state. By the time he left he was walking. So he was a quick learn, were the other one that she has wasn't.

I: So I want to go back to the example you were talking about, the girl with the high tone, and you could tell that she was uncomfortable. How did you know that she was uncomfortable?

R: So that young women **** she had a trach and I can't remember if we were capping her so I can't remember if we were just occluding her airway or not so that she would have to breathe through her mouth and nose. But she had a silent cry and the reason I say that I can't remember if it was because we were traching her or not or if she just had vocal cord paralysis because if all the intubation and everything. I just remember that her face would contort and her mouth would open and the trach would oscillate it was almost in that sobbing state. So for her it was very obvious, she would tense up so her muscles with all her arms and her legs would tense up, she had a tendency to scissor her legs. So her legs would be scissored closed and her whole body would be this tense tight mess. Her face would sometimes get red, for her it was blatantly obvious. I can think of other individuals, this young women that I mentioned earlier ****, when

I roll she doesn’t… when I roll her in bed or when I grab her arms… when I grab her arms she’ll pull back whether that is reflexive or not, but there is strong resistance when I move her arms around. But other than that I don’t see a lot of facial changes the biggest thing for her is her eyes. You mover her around you got, side to side, from sitting to laying down, any of those positions her eyes become like huge saucers. So that is one way I know that she is experiencing something and potentially is overwhelmed. There is other people whose heart rate and breathing rate goes up. There are other people who get red in the face. So I think if I had to summarize it, I think we are looking at physiological changes. Sometimes I hear some form of noise, I would not necessarily call it voicing but sometimes I will see that. Something I look for in a prone position or that on your belly position is looking for increased drooling or even some vomiting. Drooling I kind of expect just because of the position of the head and the face, but anytime someone starts vomiting in that position it is time to move. I think that some of thing I find… that I had to explain to family members who are observing somebody be uncomfortable, talking about pain versus a response. And I think even as a clinician we have to be aware of that. I may not know somebody’s response to something being annoying, to something being uncomfortable, to something being painful, maybe the same kind of response for them. So, I think I have to be mindful of what is what and I may not always know. But sometimes family members will see one response and think of it always as pain. The family members can be a little bit limited at that stand point as wanting to restrict as a clinician, what I’m trying to do because they perceive that it’s painful and other times family members read in a lot to what they are seeing so I think that I need to be aware of that as well so that I’m not totally allowing a family member to influence what I’m seeing and I still keep my clinical mindset on. That is not to say that I’m not always telling a family member, ‘oh no that’s not pain” because sometimes that their way to cope.

I: So how do you circumvent that, how do you explain the difference in responses to the families?

R: I think that one of the ways… I think it depends a little bit on the family members. So if there is someone I think is overall… I perceive that they are coping at some level whether that means that they are helping out and accepting the change in mobility. Whether that means that they

are… When I say helping out I mean helping to roll, kind of getting their hand quote unquote dirty, getting in to what their family member is doing so that they understand that not all touch is causing this response. Then I'm able to have that… 'so you notice when I do this and this you see how they are responding just because it is a stimulation, whereas when I do this and this that may be uncomfortable so I'm responding to that'. So I feel more comfortable having those conversations and being a little bit more direct saying 'not all touch is painful even though so and so may look they are responding in a way that is painful. Right now that is their only way of responding.' They have one response right now and that is their response. So that is usually how I explain it. I think for family members who have sort of made up a whole story like 'well when the wrinkle their nose like this…' and I don't say this to be um flippant but I have heard it a lot. 'when they wrinkle their nose it means they have to go to the bathroom' and so that is when I immediately have the nurse and have them come in and when they open their eyes that means they want their eyes wiped and when they move their hand they want this… if I'm not seeing that correlation as much I am going to acknowledge what the family member is observing but I am not necessarily… I think that I'm just cautious in how I support their observations. I am going to acknowledge it, because I think that after this type of injury there is no sense in me saying 'what you were perceiving is completely wrong' it may not be linked to fact but they are observing something so acknowledge like 'yeah, it seems like she is wrinkling her nose a lot' a little bit of that reflective listening where like "yeah, it seems like she moves her arm a lot around this time' and then start again talking… 'what I want you to do, you're going to be the ambassador you know her better than I do, so you're going to keep watching her and I want to see that consistency so not only is she doing it for you but she is doing it the night nurse, for me, she is doing it for the physical therapist. So really emphasizing that idea of 'you keep watching, you keep observing because you are an important piece of the puzzle' I think where some, in my perception where some clinician can kind of get into trouble is when they say 'yeah, you're right, everything you see is exactly right' because then they start facilitating a little bit of false hope. Or on the other spectrum when they say 'no, what you're seeing is not true' because then it becomes a well I'm going to show them kind of thing. Nothing else acknowledging what the family member is experiencing, whether or not it is necessarily truthful I don't feel like I'm being shady I'm just being supportive of where they are at.

I: Okay, so I want to come back to this idea, you talked about watching people come out of a disorder consciousness and that being rewarding. So, can you describe, maybe if you want to pick one person in particular, what that looks like, how do you know that they are coming out of this disordered state of consciousness?

R: I think that there are probably, well, I would say that there is the clinical guidelines, you know, functional object use, functional communication, um I'm trying to think of all of them. Um, oh and pretty consistent localized responses, for me those are the clinical things. But the process to get there isn't always they wake up one day… they are not always going to be that guy who I shaved one day and he didn't respond to anything and the next day he is brushing his teeth, that is literally what he did. Most people it is one day I hand them the wash cloth, since I'm and OT I'm working a lot with self-care things, and wash their face a little bit and then I hand them the washcloth and say 'oh, can you finished' and nothing. The next day I put the washcloth in their hand and they grab it with their hand and the next day they bring it up towards their face. Seeing that sort of progression, I think that… Um, can you ask it one more time…

I: So, talking about watching people come out of the disordered state of consciousness and how do you know they are coming out?

R: Okay, so for me it is the consistency of that response to… for me it's the functional object use. Functional communication is important but so often we have people who are trached who have potential problems with aphasia, all sorts of other things. Somebody who is using objects, or using their environment functionally, for me those are the things that stand out. Again, it may be functional communication, they may reach out and use a laptop or they may write with a pen. But I think only relying on functional communication, especially that population because of the potential cognitive and language deficits. Now functional object use is also tricky because of apraxia but even if they are incorrectly using a functional object but still attempting to use that object… So even if they are brushing their hair with a tooth brush or even if they are trying to write with a razor or something the idea is there. So, that is for me… that's my go to. That would

be the thing that… Just like somebody with motor movement impairments, so we often say I can't, not that I can't but it's hard for me to make a person move if they do not have movement to begin with. The same kind of idea for somebody with disorders of consciousness. So, if somebody has movement or if somebody has the sort of platform for using objects I can then give them different types of objects and then generalize their responses to a lot of different things. Where if somebody doesn't have that, it makes it tricky for me to build. So, that for me is a really telling…

I: I am just going to ask you to define a couple of these terms that you used. You said aphasia and apraxia, can you explain those.

R: Aphasia, I am probably not going to get the official definition, but the way I understand it is a language impairment that makes it difficult for someone to either receive verbal or written information and to express verbal or written information. There is a couple different types of it but it makes receptive and expressive… so understanding language and then being able to communicate a variety of different types of language, it makes it difficult and so sometimes it can limit somebody's ability to communicate verbally or using written language. Apraxia is also very tricky because it's basically a difficulty… It's when somebody has a hard time motor planning to… It's when somebody has a hard time using objects appropriately. So, earlier I mentioned using toothbrush as a hairbrush or using a razor as a pen. So, recognizing that there is an object and they want to use it in such a way but they are not using the object in the way that it is meant to be used. Sometimes it is when somebody just can't quite spit out the plan, they see a spoon, they know it is a spoon, they tell you it is a spoon, but they cannot figure out how to use the spoon so they just don't use it.

I: Perfect, and then one more thing, can you, kind of describe for me this idea of functional interaction with the environment. So you talked about functional object use and then you has also mentioned functional involvement with the environment so can you explain that a little bit?

R: Yea. So, one of the… I mean one obvious one might be like putting somebody in front of a sink and seeing how they respond to it or looking, you know, there you have the sink you got the mirror, do they reach out and put their hand in water, do they look up at the mirror, are they sort of engaging with the environment as you would expect them to. I would say a wheelchair as well, but a wheelchair is tricky because most people who are in a disorder state of consciousness weren't previously in a wheelchair and weren't exposed to it, but some people do initiate pushing the wheelchair which is always fascinating to me. That drive for mobility is amazing to me, people when they need to move or what to move they will find out a way to move. Um, which is why some of those early mobility things… Like if I could do anything I would create a… Take what **** is doing and put that into the disordered state of consciousness. If I could put everybody who is in a disordered state of consciousness in a power chair and give them the ability to move in whatever capacity they could, I would because I think that that's huge. Anyway, the next uh… What was I going to say?... Oh, the other thing in terms of functional environment is I look for automatic responses to change in position. So, often times I am working with people in a sitting position or maybe in a laying down position. So, in a sitting position if I lean them over to their right side and that person starts to give me a little bit of what we call like a righting reaction in their trunk so that means that if lean them over to their right side the left side of their trunk starts to contract a little bit to attempt to keep them in that upright position. Um or if lean them forward if I am pushing them forward and their head starts to come up or their body figures out a way to kind of respond to that change in position. So, I think maybe it is less of functional interaction with their environment and functional changes with position. So, less of response to specific objects and more what is happening overall to their body. I also think it is interesting to look at how are people responding to lighting in different ways. Are they kind of blinking or closing their eyes when I am like, everything is straight up florescent lights when they are laying on their back or when they see a window are they looking to look out of that window, all those sort of things.

I: Anything else about this that you want to share? Okay. I want you tell me about a time when you had a frustrating experience with this population, one patient in particular.

R: A frustrating experience. Um, I think that for me this patient population doesn't often… the patient them self isn't often the frustrating part and most often my emotions don't necessarily go to frustrating, like if when I think about patient in isolation and more of like disappointment, sadness, and a lot of those… I use those emotions or those descriptions because for me I perceive that I am not… I know there is only so much that I can do but at some point, you kind of, in your heart, as a clinician give up a little bit because you are not seeing that reciprocal response. Or you are not seeing the improvement you would have expected. So, there was one women who she had a cardiac arrest while running, she was probably Rancho 2 if that, she was one of those rare Rancho 1s that we get. She just wasn't… Rancho 1 meaning she wasn't responding to anything and it was basically just decided that her husband was going to take her home and take her off life-support. And it was hard because we were all everyday saying like, 'oh I think she might have responded to this' and emotion more went to that sadness, because I as a clinician knew that I had reached my limit and I think that is the hardest thing with this patient population is that some point if they're not emerging, you don't see a progression and you move to more of a chronic state, and I think that is where I don't necessarily always get that perspective of being on the inpatient rehab side and that is why I think it is helpful to… I know that there are resources for both clinicians and just the lay population in terms of books and websites and things where you can learn from sort of family members who have loved ones in chronic states of disordered consciousness. So, for me the most quote unquote frustrating patients are the ones who don't improve. Even someone who has localized responses but does really emerge to the point where their fully conscious, you know, that is easier to kind of work with than patient who just has no response or isn't making progress. I think the frustrating part honestly comes with the families. Um, I know as much as I can tell myself that there are not responding to me as an individual, um, I think that watching the families struggle and then watching the family then turn against staff is probably the hardest part. Because family can, and to np fault of their own really, just become nasty and it gets uncomfortable because they don't understand why their patient isn't moving as far along as their neighbor. And I think because most people try to keep a cordial interaction, keep people… kind of keep it at bay. And I think that as a clinical team we do a pretty good job of having those conversations. The physicians try as hard as they can to at the very beginning start setting expectations, outlining expectations to the family members but just

like with patients who, let’s say, do emerge from a disorder consciousness state and then kind of start plateauing and then family members get really mad because the family members see all this progress and then the patient stops. I think in the same way; this population is really hard because family members expect to see changes just like they have seen elsewhere in the unit and they don’t their sort of hopes are a little bit dash. So, you see a little bit of that, you know I always wonder how I would be in that situation would I be the person who accepted the loved one wherever I was, would I get angry, would I continue this sort of façade of false hope and expectation. Um, I guess I shouldn’t say false because for them it is real hope. So, I think for patients when I think about it that is where I just get sad and disappointed with myself and it is easy if I think I am honest with myself I think it is easy to blame the patient and be like ‘they aren’t doing anything anyway’ but I think some of that comes because I am disappointed that I couldn’t do enough. But I think that is where as clinicians we kind of talk about and realize that you can only do so much. You can’t change the patient… the patient’s social structure before they got in the hospital, you can’t change it while they are in the hospital. Patients who lay in bed and have no family around are also just sad and disappointing.

I: You talked about this idea of being chronic, can you explain that a little bit more?

R: Yeah. Um, I guess it… the timeline could probably vary. I think that most of the patients we see at the hospital or on the floor that I work on as being more of an acute stage meaning that their injury happened most likely within the last 6 months and the person is still… maybe they are fighting infections, maybe the doctors responsible are still making a lot of medical and medication changes, maybe they still have wounds that are healing. So, over all their body is still in an active state of healing. And the reason I say up to 6 months is because sometimes people have a long time that they are weaning from the ventilators. So, coming off the ventilators so that they can come and be on what we call room air or just with oxygen. Sometimes people just keep spiking fevers and getting infections whether that is because of a surgical wound on their head or elsewhere. So, those people in that sort of beginning 6 months, I feel like, are still in more of that acute stage. Some people are in a disordered state of consciousness and they only last a couple weeks, whereas the patients I am thinking of or at the almost 6-month stage. Chronic to me is

less of what I see on a regular basis at the hospital, but I would say that is anybody like over 6-months to a year or somebody who for all intents and purposes sort of started to plateau in terms of their response, their body is settling in a little bit to the changes in the spasticity and muscle tone so that they are starting to tighten up and contractures are starting to form. Their starting to get some more long term conditions, I guess I would say, that my impact the ability for me to provide therapeutic care. For example, contractures… their muscles are tight and in acute stage I can kind of work with those in a chronic stage it may just mean surgery. After a while they may just get to the point where medications and repositioning and casting and splinting and that kind of thing isn't going to make the different, it is going to have to be a more intense intervention. Um, I also think…we had a woman a couple years ago, who… she got to a 18 months to 2 years, I can't remember which one, after her original injury. She had basically gone straight to a skilled nursing facility after her accident. So, she really hadn't gone to intensive rehab, I don't know if she would have been appropriate, I don't know what she was like beforehand. But at that point there wasn't… I wasn't expecting a lot of change and so I would say that the chronic population are the ones that I don't expect a ton of change to happen. And so I kind of work on training the family, trying to figure out what will be the care for like a maintenance plan, not that I would bill it as that, but I am sort of putting into… putting everything inline to get ready for more of a maintenance or a home program.

I: Anything else about this topic or other disorder consciousness population that you want to share?

R: Um,

I: About your experiences?

R: No, I think… I guess the only other thing is like, the frustration also come from just the health care system and that is probably not super related to this study but I think that the patient

population in disordered state of consciousness they aren't really given a fight for a chance to fight. And I feel like that starts well before inpatient rehab. And people are chosen, you know you'll go to a skilled facility, you'll got to a long-term care and I think about all the people that we don't have access to in terms of providing intensive rehab. Um, you know being able to tolerate 3 hours of therapy looks different for different people depending on where they are and their recovery process and I think people aren't often given the benefit of the doubt that they can't actually participate, because participation looks different. So, I think that's the other frustration that I experience is seeing somebody like the young women who was 18 months to 2 years' post if I had gotten her earlier could we have helped manage her positioning, her contractures, and those kinds of things early on. Um, maybe, I don't know. But without having access that patient population and being able to keep them for longer than the two weeks to a month how are we ever going to know. You know, how are going to know if the positioning that I want to work on and if putting somebody in prone 3 times a week for 6 weeks makes a different in their hip positioning and their neck and their posture and other things, or is that not actually working, but I never know because we are always sort of… it's like 50 first dates is it just like trying something new for every patient. I think that is the frustrating thing and it is less of individual patients.

I: Okay. Thank you

R: You're welcome

Clinician Interview

Interviewer: EM

I = Interviewer
R = Respondent

I: We are conducting this study that is funded by the Department of Defense and the Veteran Administration for patients who remain in disordered states of consciousness during their first year of injury. Part of this study is to better understand how clinicians and family members or caregivers communicate changes they have observed in this patient population. Today I want to talk to you about the care that you have provided for patients who were in that state of disordered consciousness, meaning they were considered to be in a coma, vegetative state, or minimally conscious state. I want to learn from your experience because we want to understand the language that clinicians use to communicate improvement, decline or maintenance, and plateau in neurobehavioral performance when treating this population. This interview is being recorder for research purposes. The recording will only be shared with authorized members of the research team. Information you share will be kept confidential. Any people or places identified during the interview will be removed in the transcription phase, and it won't be passed on through any analysis or dissemination of this study. Participation in this interview is completely voluntary and you may choose to end your participation at any time. If you choose to end the

interview any information already collected, via audio recording can be used for analysis. Do we have your consent to continue?

R: Yes, you do.

I: Okay. So, I want you to think about your experiences with this disordered consciousness population and I want you to tell me about a time when you were surprised by one of these patients.

R: Um. Man, I can tell you about a bunch of times. Surprise in terms of starts really low level and then progresses really quickly or comes out of it?

I: Sure.

R: Um. I had one patient that presented as a minimal consciousness Rancho 3 and no head control, no visual scanning, no form of functional communication or anything to elicit that he had active awareness of what was going on. And then this patient who is under my care right now, he is status post 3 months from when he got to here. He is now ambulatory, transfers at a min-assist level and is preforming all sorts of functional mobility, but it didn't happen super quickly, it happened very gradually. But on looking back on where he was to where he is now, it is very exciting when he started communicating towards us, following our commands, and all of that progressing now to a Rancho 5. There were other times where, I had another woman presented exactly the same way but even lower level. The first subject that I had just spoken

about he was motor restless on one side of his body, this female was not motor restless at all, she had fluctuation tone and she was still in this autonomic storming. Unfortunately for her, her stay was a lot shorter, it was about a month because insurance ended up denying her. But although her head control was progressing, the speech and language pathologist found a functional way of her communicating of her sticking out her tongue. And from there we were able to use that to progress therapy but it was an exciting and surprising moment for all of us.

I: Okay. So, I am going to go back and I'm going to ask you about some of these terms and these behaviors that you were mentioning. So, you mentioned motor restlessness, can you describe for me what that looks like?

R: I would say imagine being really amped up on caffeine where you can't be still. And again this is all within the realm of if the patient is paretic or not, or has paresis. To some extent you will see taping of the leg nonstop. You will see continual shifting of the weight anyway, trunk, arm, hand up/down. I just like to think of it as someone who had 1000 cups of coffee. But the restlessness is not active to a specific command. If I were to say tap your leg, they don't tap their leg, they are just doing it regardless.

I: You also mentioned fluctuating tone, can you explain that a little bit?

R: Yes, tone is always a really fun one to explain. So, we all have tone has humans and after an injury we can have less tone or more muscle tone or more, I guess, muscle mass. Let me thing, that's a grey one. It looks, again… I want to describe it as, like, some is… For example, the way

I see it is someone's feet are regularly positioned, they are angled and then all of a sudden, you see them pushing downward, like they are pushing down on a gas pedal. But it is not drive, again, from active volitional movement its drive by the centers in our brain that control the amount of tone we have.

I: The last one I wanted to ask you about was the autonomic storming. Can you explain what that is like?

R: Yea. So, the centers in our… our brain is like a thermostat where it autoregulates things that we don't need to worry about. So temperature control, alertness, arousal, all that. And breathing and all that and so forth and so forth. After a significant injury to the brain, those structures that normally control all the stuff we don't really need to worry about are injured. So, often times I'll see patients profusely sweating, or they have very high temperatures because their body can't autoregulate it or it's very hard. Their heartrate is really really high, their blood pressure is all over the board from a vital standpoint. A lot of work looks like it is being done on the body.

I: And would you associate that with the lower Rancho levels?

R: Yes.

I: Okay. I want to go back, you gave the example of the functional communication where you had the woman who was sticking out her tongue. Can you give me an example of how you used that during your treatment or during her stay.

R: Yeah. For one, it is really nice for, 'are you in pain'. Granted we can look at facial features, to maybe give us an indication that someone's in pain but if there is paralysis on one side maybe you can't really tell. So, 'are you in pain', yes or no, or however I can use it. If a patient wants to… Then I can give them more choices into their treatment too. 'Do you want to work on support and standing, or do you want to work on head control, or shifting your weight. And then, I'll say commands they can follow, I either just ask a yes/no, or I say those three and then I tell them 'I'm going to ask you three, here they are stick out your tongue which one you want to do when I repeat them.' So, it gives them a little bit more control too. If the patient as more control, then I feel like they are more likely going to participate in treatment sessions and you are going to get better results from that.

I: So, the sticking out of the tongue was her 'yes' response?

R: Yes. And then, sticking out a tongue but moving it horizontally was the no. Sometimes, and then sometimes it a blatant closing of the mouth.

I: Would you say she was consistent with that?

R: Not initially, but it came with time and again her level of arousal too. Because in the earlier stages her sleep wake cycles, like a lot of patients, are altered, but hers especially. Even doing the smallest movement or activity can be very fatiguing too. So, if she was very lethargic or sleeping you couldn't get it. But then as her awareness and her alertness increased for a longer

period of time then we were able to make it more consistent, and then yes, she became consistent.

I: How did you know that her awareness and her alertness were improving? What did it look like?

R: Eyes were awake during my session. There was more active with her. She had visual scanning. So if I wanted her to look move toward a certain direction, I would see her eyes go towards that direction and then I might see shifting of weight. A longer tolerance to an hour session as well. So I got a little bit more each time out of that session than just the first time.

I: So what do you mean you got more?

R: So for her. With her we started lower level with head control. She was able to initiate head control more, so, it was already weak to begin with. Her head was flexed so her chin was lowered, but I was able to feel her cervical extensors now pick up her head, holding it for longer periods of time, upright, unassisted, being able to initiate head turn to the right and left within the restrictions of her tone. Yeah, so longer periods of time able to do more repetitions.

I: And you said she was one of the ones that surprised you with how well she did? Why was it surprising?

R: She is also didn't typically follow a standard, there are a bunch of different theories for recovery tap up or tap down, but she was one that had communicated like functional means of communication before she had anything else and I would have liked if she stayed longer to see where she was able to then come physically from that, but generally some we see, generally I see when someone starts coming out of that minimally conscious state we tend to see things coming together, so we might see the tongue sticking out as a functionally means of communication or blinking for example or whatever it is in conjunction with head control in conjunction with active voluntarily movement in a limb or an upper extremity or what not. She also started so sick too. She couldn't regulate herself. She was always sweating. She always had spike temperatures.

I: So did you say that when you first started with her, you weren't expecting her to do well?

R: No, we didn't say we wasn't expecting her to do well. I think part of it was her brain scans when we reviewed them with the doctors during neuroradiology rounds. The images showed significant injured to the deep brain structures and those deep brain structures are the ones that are pivotal for sustaining life, so controlling your breathing. On top of moving outwards for areas that were injured such as communication, other areas of the brain that were injured such as your motor function, but I wasn't entirely surprised either because when a patient moves from acute care towards back to where we are now a lot of factors are changing. Meds too because in the earlier phases a lot of the medications that the patients are on are sedative so getting her on a stimulant helps to and then you can kind of see where they are at functionally. Her injury was also further out than we would have liked to see when she came here, so to me that was a sign

where prognosis isn't great when the injury, where it took a while to (?) from an event to come towards us.

I: So you mentioned the stimulants and how they help? Can you give me an example of whether it's this patient or another patient of describe for me what they look like before they are on the stimulant and then what the changes are once those stimulants are on board?

R: Generally, speaking I think I'm going to talk grossly, not one patient specifically. Decreased level of arousal so maybe just eyes closed and then now their eyes are open. I have also seen just very slow, lethargic, sloth-like, not a lot initiation, so maybe they're so lower level their eyes are open, they visually scan something, but you can't get anything more. Then I might see just more attention, more levels of arousal, but as we start progressing I will tend to see that motor restlessness that I was talking about earlier, so it might not specifically follow a command, but now I'm seeing a lot more active movement and jittery movement.

I: So before we move on, is there anything else about this patient that you started talking about that you want to share?

R: Yeah, with that low level patient it was unfortunate that insurance ended up denying her because technically from a billing perspective across all disciplines PT, OT, and speech and I don't want to talk on behalf of speech and OT, but as a team we would always talk together, this one patient was making these gains. Her storming was getting better. She now had a functional means of communication. She had longer time to hold her head up. She was able to do more

with her head. If you can do more with your head, you can have a second means of communication now. Insurance ended up denying her because technically on paper there were no objective means to show progress, so it was really hard for us to see that this patient now attending and participatory in the best way that she could, but her body was just lagging behind so on paper objectively she wasn't making gains when we saw she was.

I: So you mentioned the team, PT, OT, speech? How important is that collaboration with this patient population?

R: Extremely important. Very,very extremely important because speech is the one that saw the tongue sticking out was a way that she could communicate with someone and then the speech rolled with it and then let us know this patient has voice and while, as a PT, don't focus on communication you need the communication to progress forward in terms of your treatment. A lot of us will work on similar things too such as visual scanning and fixating because that's something that we all really use as humans in general no matter what.

I: Okay, so we are going to switch gears a little bit.

R: Oh, sorry one last thing. The last time that was important with the speech, speech, OT, PT, where so speech collaborated with me because I, as a PT, can put the patient in a supportive standing position through weight bearing through your joints, through the use of the machine that we had to stand this low-level patient. Research shows that it activates your deep brain structures or reticular activating system increases your level of alertness and arousal. So, then

speech was able to use that. I put the patient in standing and then speech wanted to do a session with the patient in that supportive stance. So, that was a collaborative treatment session where we were able to really maximize our patients gains.

I: Okay, so I'm going to switch gears a little bit and I want you to tell me about a time with a disorder consciousness patient where you had a frustrating experience.

R: By frustrating, I would imagine being remains essentially the same level of consciousness I guess I would gather from it, aside from the one I just mentioned, someone booted them out and I don't think that was appropriate. The other in terms of frustrating would be you see someone that is lower level and unfortunately they end up remaining lower level, so there are no signs of visual scanning, visual fixation, localizing to auditory stimuli, responding to tactile stimuli, no writing or balance reactions, no initiation, no slight initiation or trace amounts of initiation, head or trunk control, something that is called "doll's eyes" so you shift the patient down and to the left and their eyes just immediately go down to the left like a baby doll with those eyes would versus the typical response if you shift someone to the left, they're going to want to go to the opposite way. A lot of those reflexive, deep brain structures, that's when I know they are not progressing or even just not being able to continuously arouse a patient throughout the session to they're are sleeping through most of it. High, high, high levels of tone on top of it where they're almost essentially contracted and then they remain like that for the remainder of the stay or they have, they are so contracted, for example, at their ankles which is very common that it limits my treatment session as well, so I can't necessarily get them into a stander because it would too

much pressure on their joints or they're at high risk of spraining, breaking etc. So then that limits my sessions in terms of all the different things I could for treatment.\

I: Can you describe for me what being contracted looks like?

R: Well I guess it depends where in the brain you have an injury in terms of posturing. Contracture can be what ever the position of comfort that one tends to stay in, those muscles then shorten and if they shorten over a time the way I like to describe it to other family members is that position will get stuck and you will not be able to stretch out of it at all. It will be fixed in that certain position. Oftentimes, the way I'll see it, for example, at the ankles is the toes will be pointed down like you're pushing down on the gas pedal and the bottom of your foot or the sole of your foot will be turned inward.

I: You mentioned, the lack of visual scanning and fixation. Can you describe for me how those two look different? Scanning and the fixation.

R: So visual scanning, you're searching around in your environment. This is within the patient's capabilities. There is no specific or huge cranial nerve deficits because if you have a cranial nerve deficit to your eyes it's going to limit your range of motion or the ability to look forward, so generally speaking this the ability to with your eyes search your environment. Fixation is, let's say a patient has a tendency to look up and to the right or gaze preference that way, but I put music down and to the left and they hear it so they can localize to it and then they look that way again within their limitations and what they have and don't have.

I: If you can, pick one patient who started low and then stayed low throughout their stay and I want you to describe them behaviorally for me.

R. I have like three in my head right now. Are we still talking rancho 3 or rancho 4? I would imagine 3 if it's the most minimally conscious.

I: Whatever you want.

R: I'll do the one I first thought of so, the rancho 3's. Behaviorally, no active participation, no command follow in any way. Essentially, it felt almost the lights are on, but nobody is home.

I: Can you explain that a little bit?

R: They are awake meaning that their eyes are open, but I'm not seeing any of the things I had just spoke about. So the visual scanning,visual fixation, no active or trace amounts of movement what so ever. Very quiet sometimes, oftentimes, I'll get moaning, but it might not necessarily be related to what I am doing. Sometimes it is. Sometimes, I'll get moaning as a pain response, but the pain response is very reflexive. So stretching or discomfort, For whatever reason and I don't know if it is med driven or what, but later those patients, this one specifically, it's almost like sucking on a pacifier, but nothing being in the mouth. So a lot of those mouth noises. Their head is down low. They're sitting just totally hunched over, not continent. So likely, to be profusely sweating or sweating when doing any functional activity.

I: Okay, so I want to go back to this idea of sometimes you'll get moaning and sometimes it's a pain response? So how do you know if it is a pain response or if it is not.

R: The best way to figure that one out is by stretching someone. So, obviously as a PT I know when a muscle is tight and so if I go through that stretch where I would expect someone, anyone to be able to feel it because it hurts or its uncomfortable, then I would see that pain response on their face. Moaning, but on the face, facial features that just look like, you can tell that they are in pain.

I: Can you describe those?

R: I know, I am trying to think. Scrunching up of the face, frowning, something like that.

I: Anything else about this patient that I didn't ask you already, that you want to share?

R: Not that one. There was another low level patient that was cool and physically he was so low level, but there times where he would be following commands and that was also really cool. He would like wiggle his toe. I just saw some movement, I didn't know if it was tone driven or not and then I would ask, "Can you wiggle the toe?" It was mid-treatment session, so I guess the overall theme would be not to keep trying everything because you don't know if someone is actually listening or not or (9:33-30?) during a certain time period and so it's important to always be trying new things.

I: So these patients that start low and stay low do you know at any point during their stay that they are going to remain in their current state?

R: Somewhat, I would say it's absolute and for some people if you have time you do see gains and progress, but indicators for me are the age of the patient, so if they're younger you're more likely to have better adaptability of your brain to heal. So younger, how long the injury since the time they've been here. Their level of activity and actually intelligence or IQ. All of those relate to prognosis, so that gives me an idea of the gains I can make, but for some people it's a time thing too and maybe they'll make gains, but it just won't be within the month or two period that they are with us. Also, I will see if they start low, the ones that have the best prognostic indicator is where I see quick gains fast. So, they don't have high control one day, literally within a day or a week they have high control and now they are doing some sort of communication or I see motor restlessness now. If I see quick jumps, then I know we're going to be able to do more or the potential to progress is going to be there versus someone who is still acting the same way that I saw from day one.

I: I want you to tell me about your most memorable experience with one of these disordered consciousness patients. Try to pick one, if you can.

R: The one I'm going to relate to is one that started really low and then all a sudden just woke up. It kind of was like the one I just described to you. Started really low wasn't visually scanning, wasn't fixating, wasn't showing a means of attending or awareness of the environment. Then within the week they had head control. Their vitals were stabilized. They started

progressing in treatment in terms of we want someone to be weight bearing as soon as possible, but in order to do that we need to make sure their vitals are able to tolerate it because they've probably been horizontal for so long. There is a gradual progression. So starting at the tilt table, the vitals remained pretty solid. There was no (storming?) and then from there all of sudden head control and then from there it was now participating more in sessions. Speech would come back and say I found a way to communicate with them, try this in your session and now you're incorporating it. Then they are starting to voice or mumble something. Then this guy who is pretty weak still is attending to things, but not 100% accurate; he was able to stand for the first time within those two weeks or whatever it was, but it happened so fast and it happened. It was just really, really cool. By the end of this stay, he walked out of here without a wheelchair. I mean there was still a lot for him to do, but it was so exciting to see from where he came to where he was able to go. Word has that he passed his first (5:16?) class with an A. Yeah, it was just really, really cool stuff.

I: So I want to go back to this idea, you've mentioned a couple of times the increase in participation. I know this is going to be challenging. Can you put that into words? What does that look like?

R: Well I guess, the attention is part of that so, now if you know that they are able to attend in some way. So, you talk and they look at you. Or you ask them to do something and you can see within the confounds of what they are capable of doing, if they are hemicratic you're not going to ask them to move that hemi side, you're going to ask them to move the side that they have capability of moving and use that first. It kind of goes back to the whole sets and reps thing. So

can they do more in therapy that when they started in terms of a time component, before fatigue, or repetitions whatever that might be so standing, or sitting, or what not.

I: So these lower level patients who don't have that communication ability yet. How do you know if they are fatigued?

R: Well I guess part of that is in terms of alertness. So, either eye primarily open in the first half of the session and now they are snoozing and you can't arouse them by shifting their weight or repositioning them or any of those features. Or maybe I'll get them on their stomach over a mat because that really facilitates cervical extension or picking up the head and I'm seeing bobbing o the head earlier on and now I'm not seeing any of it. It's just hanging down later, so to speak.

I: This patient who started to progress really quickly, was there anything about this patient initially that told you he was going to progress quickly? Aside from his prognostic indicators.

R: Other than he just, he changed his presentation fairly rapidly, so we ran with it, but at first eval there was nothing that could indicate that to me when I evaluated him aside from yes he was young and he was active and he was really smart, but his brain scans were terrible and if you walked into the room and you saw him it looked terrible.

I: And then he started to progress, so then what changed? What looked different?

R: He picked up his head, so he had head control then and he kind of progressed, this is kind of ideally and he was an almost peripheral candidate where he progressed in all areas of PT, OT, speech so in PT now he held up his head and speech now he started following commands a little, showing evidence of following commands so speech ran with that and found out a way to do things. Or even though his head control was weak I saw that he had muscle movement. So, once his vitals were stable I was like okay well let's just use the little that I see and see what happens and I got him standing for the first time in the parallel bars or against the mat table. He was able to do it. He was just able to progress in terms of treatment.

I: It's so hard to put into words.

R: It is so hard.

I: Anything else about this patient?

R: Not that I can recall or think of.

I: Anything else about your experience with the disordered consciousness population that I did not already ask you?

R: I think that's about it. It would be really great to come up with objective measures to show insurance that we are seeing these changes, these functional changes, but they just for some

might just be over an extended period of time, unfortunately. It would be nice to show that we see changes objectively in a way that is just so hard to.

I: Okay

R:Cool

Clinician Interview 6

I= Interviewer

R= Respondent

I: …Do we have your consent to continue?

R: Yes

I: Okay, so I want you to think about these patients in the disordered state of consciousness that you've worked with and I want you to tell me about your most memorable experience with one of these patients.

R: Let's see. I think probably…I can think of kind of collectively what my most memorable experiences were and it's usually when they come to us in a state of minimally conscious state so any sort of inconsistent response to environmental stimuli that we're not really sure if they are taking in what we're saying or they're not able to communicate with us in any sort of way. Usually the most memorable experiences are when they do start to do that, so when you go in and say hi and they look at you and they say hi back or they might just look at you and that's, I think a really powerful thing because it's something that you see. I think a lot of times your hear from family "Oh they did this" "They moved their arm" but when you see it and you see it consistently I think it's really exciting. So I think it's usually any sort of patient that I've seen

more reaction or response to either what I'm saying, what their family is saying or also I really have enjoyed when I'm not sure if the person can hear or is understanding what I'm saying if you put a song on and they mouth the words, that's always really exciting. Those are probably moments that I enjoy.

I: You said the increased reactions and increased responses to different stimuli or commands. Can you think about maybe one specific incident and walk me through what that looks like.

R: I think it's usually when I am first walking in the room and greeting them and they turn to look at me or they might even, I might reach my hand out to shake theirs and there might be a little bit of initiation to shake to my hand. There's times when I see patients interacting with family members and you can almost see a change whether it's a smile or else they relax in the presence of family members. It's sometimes a little more subtle, but usually it involves…we kind of think of those four areas of emerging from a minimally conscious state with any sort of communication, tracking whether they are following something, their face in a mirror or family photos or music video, so those two things. Any sort of functional object use. As OTs we're always trying to see if we put something in their hand that they recognize or we get them in front of the mirror at the sink, how will they perform in a functional context. So any of those things.

I: Let's talk about the functional object use a little bit. Can you give me an example of what that might look like with one of these patients?

R: Well usually I put the object in their hand. If they can initiate a reach for it, great, but usually I'm putting it in their hand and I'm giving them a direction that something they'd use with the object. So I'd hand them a toothbrush and say, "Brush your teeth," or I might hand them a hairbrush, "Brush your hair," try to keep it pretty simple. Something that most, the vast majority of people know how to use most grooming items. I did have one guy, actually I have one guy right now that he had like a spray can of deodorant, so a lot of the times I would put it in his hand then we'd spray under his arms and just to see if we could kind of get that because then you get a lot of the sensory from that like you smell it. You also spray it, so you hear it, you feel it because I'm pushing your finger down. Usually, it involves me putting it in their hand and asking them to do something with it.

I: At the beginning your mentioned inconsistent responses to environmental stimuli. Can you give me some examples of what some environmental stimuli might be for one of these patients and what their reaction might be.

R: In terms of inconsistent?

I: Or to just think about one of these patients who's minimally conscious and give me an example of what environmental stimuli might be.

R: It might be family members talking. A lot of times people will be talking and sometimes they're talking about the patient or they are trying to talk to them and they might see them kind of looking over and observing. It might be bright lights. I try not to leave the tv on. I know family members do it all the time and it drives me crazy. It may be environmental stimuli could also be PCTs, washing them up if we're doing an inserted toiling from bed level. It may be rolling back and forth some movement. I realize that might not be natural, environmental stimuli. That's pretty much it.

I: Let's pick one. Let's use the family talking as an example. What are you expecting to see?

R: I'm usually, at the bare minimum, what I would want to see is shifting gaze over to the family members talking. Any head turn would be fantastic. Any sort of usually visual or motor recognition that they are there. There's not much else that I see that I would be looking for in that respect.

I: That's perfect. Anything else about your memorable experiences that you want to share?

R: I wish I had a better memory other than when people emerge because that's always like so exciting, but I know some people don't. They last few patients I have had haven't emerged and a few have gone to live with family. I think from a family perspective it's really nice see the family pull together and really listen to what we're saying about keep talking to them, keep

interacting with them, keep making them a part of your conversation and doing the things that they like, playing their favorite music. So when people don't emerge, I like to see the family come together and that's really nice to see. On the flip side, it's awesome when somebody starts responding to what you're saying and is able to see improvement because then everybody just gets on board, everybody is just in it. I think there's two sides to it. I would say probably half the patients that were minimally conscious that I've seen have not emerged and half have. Or even seeing them like six months later, it's really cool to see.

I: So let's talk about one that has emerged. Do you have somebody in mind?

R: Yeah, yeah.

I: I want you to walk me through what this person looked like to you when they presented and then walk me through their emergence and what that looks like.

R: When I first saw him his head was down in the wheelchair. He wasn't able to pick it up. He wasn't really responding to anyone and he wasn't really able to communicate in a meaningful way. His eyes were closed. He wasn't really responding to touch and really not to movement. He required total assistance for every transfer bed mobility, everything. He was essentially, we were doing everything for him and family was there all the time. They were always talking to him and trying to get a little more out of him. Then slowly, it happened slowly, but then it kept

going. It was exciting because one day, we asked him to open up his hand and he did. Then he slowly started to pick his head up. I think, honestly, family was really essential in how quickly he recovered because they were there around the clock and always talking to him. They learned transfers right away. I didn't have to do a lot of the prep work. As soon as I got there he was ready to go. He was already in the chair and they would carry over everything that we asked them to carry over. Eventually, he walked out of here. That was really cool, but he slowly just started, I remember he was able to look at you. I think that was the first thing that he was able to do. So he picked his head up and he was able to look and he would hang it down, but for a minute or so he would be able to pick it up, look at you. He wasn't speaking. He wasn't following commands, but I think then we just went right into the bathroom and started doing stuff with brushing teeth, combing his hair, shaving like they had all his stuff there. At first he would let us help him with his hands, so I was just putting my hand on his hand and just helping him brush, helping him brush his hair and all that stuff. Then eventually he did start to help and he was able to do it. He still need some direction and some assistance, but I mean that was kind of what we worked on next. Usually, from an OT perspective as soon as they are able to visually focus on something or visually attend, then I'm going right in with ADLS, functional object use, things like that. I don't know if I'd say vision is the most important thing to start with, but it's so important because I don't know you can do a lot with sensory stamina I think that's really good too, but in terms of functional object use, usually it helps if they can focus on what's in their hand or they can see it and then initiate. It was just a really good team effort from everybody. It was so good. Everything we did the family would carry it over. He was (26:24?) in supervision for all his ADLS when he left. He still has a lot of cognitive issues, but he's back in college. He's doing really well. He started with no response.

I: So when you started to see some of these changes, I know you said it was at team effort, how did you communicate some of this to the rest of the ADL team?

R: Usually, if it was in a huddle or conferences or in the office it would usually be well he picked his head up, it felt like he was listening when we were talking to him or when I was talking to mom and dad it felt like he was listening. He would really fixate which I feel like doesn't happen a lot of the time. Usually, sometimes they might be looking all over the place, but it felt like he was very focused. It felt like he was listening and he was visually attending to what we were saying. Then of course when he started to move his hands, I'm like yeah he's started participating, he's starting to help with his selfcare. He's not a passive participant. He's actively participating. When we said his name, he knew exactly. He was able to identify himself before he was able to speak. We did the (decks?) the first week he was there, but we stopped it after about a week because he was looking at us. He was visually attending. He was tracking and able to start some motor movement.

I: How did you use the DOCS initially to inform you or to inform your treatment plan?

R:Usually, I take because I view it as all five senses. Usually I take their strongest or where they are localizing the most and I kind of capitalize on that and usually it's nice the family's there when I'm doing it because they see it as well and they can help. I can reinforce, so he was

looking at himself in the mirror and he was able to follow it a little bit. I want you guys to practice this a little bit when he is awake. Give him his sleep when he is sleeping, but when he is awake work on that. Or try to give him a toothbrush and see if he can…say “Brush your teeth” and so usually I have them to mimmick what I was doing, but usually I take their strongest or what they are localizing the most to and work on that. For him it was auditory and visual. At first he didn’t respond to the bell and the whistle, but when we said his name, he responded. Visual, he started to respond a lot more.

I: Can you give me some examples, with this patient, what localizing response is?

R: It’s either, for him it was shifting eye gaze, turning head, usually that was, he really didn’t respond much when we were touching him or moving him, but when he heard us say his name he would look up. So any sort of motor or visual response in the direction of what we were doing.

I: Anything else about this patient that you want to share?

R: Umm, no. We tried to incorporate his interest as much as we could. He liked classic rock. His dad always playing classic rock with him. And then he would actually mouth the words (22:31?). So that was cool.

I: So we’re going to switch gears a little bit. Now I want you to think about a time when felt stuck with one of these disordered consciousness patients.

R: This person had a hemorrhagic stroke while she was pregnant and it was really severe. She came to us in a lot of pain, but she was responding to really anything else. She had a lot of spasms and that made it really limiting because we weren’t able to get a clear understanding of how much she was taking in or how much she was able to respond to us because she was in so much pain. She did improve a little bit where she wasn’t in so much pain and she was able to turn her head and she was tracking inconsistently and was able to hold her head up, but we didn’t get much done functionally. That’s where I feel frustrated or stuck, is when I can’t progress them into something functional a lot of the time. Her hands were really spastic and so I couldn’t even do hand over hand assistance with a lot of things. I felt stuck in that sense because I wanted to get her doing more ADLS for herself for even just helping her do that so she could get the feeling of doing it and she wasn’t able to that so a lot of it was just like educating family on how to help her with selfcare tasks and then almost ideas of what to do to improve arousal. We worked with her with a pain specialist. XXXXXX did somethings just to help with tone and reflexes. We did like different developmental positions to see how she would tolerate it. When any sort of transitional movement or her position changes she would start to cry or get upset. She would calm down after a little while, but a lot of intolerance to that. We do bouncing on the swiss ball to see if we could, I think that helped her head control a little bit and she was able to hold it up after a little while. I’m not sure if that was really helping, but we’re okay, there’s something there. I know her husband ended up getting one and I hope he did it at home. He was

really on board with anything we did. I always feel stuck when I don't have much functional stuff to do, so it's me constantly try different stimuli to see what gets the best response from her.

I: Can you give me some examples? I know you said the bouncing on the ball and the hand over hand, what other kinds of stimuli would you try with her?

R: We did a lot of music. She loved to dance. Before she would go out dancing with her friends, so we did a lot of high energy dancing movements. We'd sway on mat or bounce on the ball. We did hand massage some of the time. I'd like encourage them to get her favorite scented lotions and rub those on her. We did a few showers where we had the really good smells. Just a lot of different tactile stim, but it was mostly I would say movement, auditory. Oh, they ended up being able to save the baby, but they had taped the baby crying and laughing and moving around. So they would show her those videos too.

I: I want to go back to the beginning when you said when she first came to you she was in a lot of pain and she had the spasms. How did you know that she was in pain?

R: Even without the cap on the trach, her face was just, almost like she was silent screaming. That's kind of what it looked like. Then she would go into these, you could kind of tell when it was coming because he legs would go into extension, her arms would go into extension, then she'd kind of scream. Now when they were able to cap her, it was the actual crying. No tears,

but just lots of pain expressions. She did that too with positional changes, but it didn't last very long. It was last for five minutes and then she was back, but with the spasms it was like all the time.

I: Did this patient ever end up making any changes while she was with you?

R: I would say she made some changes that I think family knew because they saw her every day. We could see, but in terms of functional changes, no.

I: So what kinds of changes did you see?

R: Her head control improved and her eyes stayed open for longer periods of time. She was able to track and initiate some head turning. I think tone was really limiting because we were never able to tell her motor capacity because her hands were always clenched and her head was…she had kind of a tightness on one side of her neck. She would start to turn and we would help her with the rest of it, but we weren't really sure what was holding her back some of the time. So mostly changes with head control. I never saw any improvement with upper body function. So no initiation of reach, no movement there, but in her thumb at the very end. There was a little bit of thumb twitching, but we couldn't tell how functional it was because she was inconsistent with like being able to push a button to start and stop music video. Her eyes stayed open longer. I think she was more attentive with increased time if the stimuli was always in the same spot, she

would start to turn and look toward what was happening, but no communication. Family was around the clock with her as well. I think that's what makes it hard when there's not that much improvement and they're like doing everything you want them to do. They're always trying everything and they're really attentive and then you know, of course it's not the end of the road here, but ultimately want to see a response and I think it's hard to set a discharge date and to send them home when not much as improved.

I: Can you talk me through how that conversation went with the family or one of those conversations went?

R: I would tell them what I was looking for and then tell them what we can try and do to get her there. I did talk through some of the DOCS and like why I was doing what I was doing and kind of try to give like a very basic, "we're looking at anything that can help her respond to her environment, so smell, taste, touch, familiar sounds, pictures, vision, things that will get her to almost say hey pay attention to what we're doing. Usually, after a month we say, "we're still waiting to see these things and when she starts to show us this, then we can move on to this and usually giving them reassurance that you're doing the right things and it really, this is going to be a long healing process." A few of the times her sister in law just asked me, "honestly, what do you think? Do you think she is going to get better?" I said, "I can't make that call because I've seen both sides where the person doesn't get better at all and the person makes a full, almost a complete recovery. So I can't speak for each individual person." I did say I'm not sure her arms and legs are going to make a full recovery, just because of the state they're in. They're so tight.

Nothing was happening with them. I see her making more improvement with communication before I see anything with arms and legs. That was kind of like my prognosis, but it's really hard for me to say I wish I could give you more answers of a timeline, but there's just not a timeline for this sort of thing because the brain heals and it might not heal all the way.

I: Anything else about this patient that you want to share?

R: I don't think so.

I: I want you to tell me about a time you were surprised by one of your disordered consciousness folks?

R: I had one guy who, he was really far out. His accident was six months prior, but he had a lot different medical complications so he had gone to different facilities, so by the time he came to us, he was contracted. He hadn't been showing much response to anything. Family was still very much. They were very faith based so it was very much like, well, the woman told me, "God told me it's going to be a year and then he'll be better." So I was like okay. So she said that right off the bat. So they were telling me all the things he was doing. I hadn't seen it yet, so I was like okay, I think that's awesome that you're seeing it. I can't wait to see it again. I want to see him do it again. I think initially I had some, not judgement, but kind of disbelief like are they really seeing him do these things? Is he really communicating or is this just because they would

say “he moved his hand and he waved and he would say hi” and I was just like I’m not seeing any of this. And I just couldn’t tell, I think with the whole package and how long it had been. I mean Dr. XXXX came in and was like the scans are so bad. I was like okay, alright. But then he actually did improve. He definitely, I would say emerged. He left still requiring total assist for all his ADLS and he was still very dependent, but he did actually start to talk and he would attend. His body, I think, it was almost like he was trapped in his body in a way. He would get really tired, so he was limited by closing his eyes some of the time. He was turning his head. He was looking at us. He was mouthing words. He would especially attend to any sort of mud truck races. We would put those on and he loved those. He liked to watch that. He liked shooting guns. He really attended to those things. He was able to look and speak and even reach for things. So he did have some returning function. More than I would have expected. That was my surprise. I think there’s always that skepticism as a clinician. You don’t want to skeptical of course. If the family saw it, then they saw it. It really doesn’t matter if they saw it or they didn’t because if the person doesn’t do it again it’s irrelevant. I think it’s fine to say that’s great, I’m glad you saw that keeping watching for it because it may be just a coping mechanism of there’s as well. Usually, I’m not going to have any conversations with them about, well actually, are you sure? If they don’t do it again, then we’re still at that same spot and if they do, great, then we can keep going. It’s always good to have family looking out for that stuff.

I: So you said you didn’t expect a lot of this stuff from him? What did you expect when you first saw him?

R: I expected, I really did not think we were going to get not much response from him at all just because he was so far out and he was limited a lot by his medical complications which I think we deal with a lot in patient levels. He was always spiking a fever. I don't think he ever had GI percussions or C-diff. He was always either asleep or eyes clothes not responding, so we couldn't even try anything. I would get him up and we'd get him in prone and put him in the prone cart, try just different positions so relied a lot on the head. I don't have like, if the eyes or closed it's like well what can we do to in a sense get them open or get him more awake. So that was the biggest challenge and for like weeks we just kind of thought wow he's not responding to anything and he is asleep half the time and he is storming and sweating. He's just like miserable some of the time. I just wasn't expecting us to get very far and then on top of that it was kind of like the collective big picture. The scans look really bad, he's been out for a long time, he's not looking good. So I was expecting to see much.

I: Can you give me some more examples of different things that you tried to arouse him?

R: I think a lot of positional changes. So we did, I didn't try the ball with him, but I probably would have done it now looking back because I was like this fairly early on. We did a lot of sitting on the edge and rocking back and forth from elbow to elbow, tried being in prone, we even did prone in the bed, and then sometimes I would retilt the wheelchair or work on…if I ever wanted him to work on more arousal related things I would get him out of the chair and so I was moving him, just anything to work on arousal. We did a few things with, I was able to, he didn't have a ton of, he had tone, but he was, I could use his hands to do things, so we'd wash hands a

lot, do like the warm water and I'd try to move them together. I like to do a lot of handwashing and face washing just to see what they respond to. So those types of things.

I: Then you said too, when he first got to you he was contracted? Can you describe what that looks like?

R: His elbows were very tight and he came in with these crazy orthotics splints. He wasn't really tolerating those well. His one hand was pretty contracted. One side was way worse, but it wasn't basically like you can't move it easily. So, either there is some resistance or it's been tight for so long due to tone and now you've developed something that you can't move physically, so there is some tissue shortening, soft tissue shortening. I always struggle with that because sometimes I think well maybe they can move and they're just not able to right now. Like maybe they actually have this capacity, but their body is not allowing them to move and it kind of reminds me of a locked in thing. The right arm got a little better so we were able to do a little more with that.

I: How did you know that wasn't tolerating his splints?

R: He had a lot of redness and he would almost pull out of them. So we would put them on and then maybe a few hours later we checked back and he had somehow, not wriggled out of them actively I don't think, but his hand would be in a strange position or his fist would have balled up

and the splint would have still been there and his hand was curled up in it. So usually things like that and yeah just a lot of redness. They were dynamic splints that started from the operant arm when to the forearm, wrist and hands. They were huge contraptions on both arms. So it was just like bionic man. The family hated to put them on because they were like he doesn't like them and I think, he didn't really grimace that much, so I was like well I understand I know it's probably a lot so we would try and do it alternating. Or when his right started moving more, we started to focus more on the left arm.

I: Anything else about this patient that you want to share?

R: No, again families are around the clock. Some families aren't, but I feel like a lot of the minimally conscious patients we get a lot of people that are around the clock families, but I would say it's like 60/40. The families are there 60% of the time. Sometimes we get people with no family there and are minimally conscious.

I: And how does that influence what you are able to do?

R: I mean family is so big regardless. It can be hard if the family is intense and wants answers that you can't give them, but family is like essential because they will ultimately carry over what they see and what you tell them and also they're also like those familiar people that he wants. So they're going to talk about things that he likes, they're going to always be engaging him and

they're always going to be there trying versus your one hour of the day. We've got professional and emotional investment, but they have so much more investment and then he's got more premorbid investment in them. It's a good thing for expectations to because they can see day in and day out when they come to the therapy sessions like what's happening and what he's responding to, what he's not because I think so of the harder times are…we had a guy who his family wasn't there very much and they came in like one day and had all these questions and it was like overwhelming for us and for them because it was hard to say well he's not responding to what we've been doing because there is always like well what have you been doing? Well have you tried this, this, and this? He actually really likes this and we're not great we want to know these things, but we wanted to know that like when he first got here so we could try it and they are just like. So it's nice if you can have that kind of communication right off the bat and then the carry over.

I: Anything else about this patient population and your experiences that I didn't ask you that you want to share?

R: I've learned a lot about it that there are so many more outlets for therapy approaches that I never thought about.

I: Like what?

R: Well, how there is almost like a, somebody can total assist for ADLs their entire stay, but they'll still show progress and I think like the (Fin?) that's what PTs have to go by so we're always like well how are their ADLs. They almost like take a step back from ADLs and saying like well can they recognized this object, can they recognize their family members. All the precursors to selfcare, those things that are necessary like visual attention being able to hear and follow simple instructions, things like that.

I: Perfect. Anything else?

R: No

I: Thank you.

Clinical Interview 7

I: Interviewer

R: Respondent

I: …..do we have your consent to continue?

R: Yes.

I: So I want you to think about the patients that you've worked with in the disordered state of consciousness and tell me about a time you were surprised by the patient?

R: Time I was surprised? You're looking for like specific examples right?

I: Yeah. Anything.

R: Yeah. I'm trying to think about a very specific example, I'm having a hard time thinking of something very specific just cause it's been awhile now. I can remember moments where like a patient would follow a command for the first time, like that being like, "Woah, did they actually just do that? Did I actually just see that? Or was that sort of random? So like a lot of times it would be something like a visual target kind of thing like look at this look at that kind of thing and the patient, you know that first time the patient would actually do it, it was like wow. I think they did that and being surprised in that moment about that happening. Or like when you were trying to get patients to communicate in a different way I can remember one patient that I picked up like one time just like randomly floating down back down to 10. Where he was in a minimally conscious state still, but tech center had just been in there working with him and had said that he was somewhat consistently communicating with his tongue and then I was working with him and I couldn't get him to do anything like that. It was stick out your tongue for yes and pull it back in

for no or something or move it around side to side for no and he wasn’t doing any of that for me and then I remember coming back to see him and it was just like spot on consistent like you’d ask him a question and like stick out the tongue for yes and like boom, you know side to side for no, boom. He would just like do it really, really consistently and so I remember being like, “Oh wow, that’s different! Like that’s a big change.”

I: Do you remember how much time was in between?

R: I don’t. It couldn’t have been that long. Although, I know he ended up being on 10 for awhile, but I would say it would’ve been in the time frame of like 1-2 weeks, not months between that time from when I first saw him to when I saw him again because I was still like real familiar with him.

I: So you talked about the command following for the first time? Can you think of anything specific? A specific command with a certain patient or?

R: It’s been so long now. I may not be as useful to you.

I: It’s okay.

R: Like trying to run through some of my patients in my brain. I could remember one patient who I remember you could ask her when she first came in. She wasn’t consistent, definitely still in a minimally conscious state, but you could ask her to like point a finger and she would inconsistently do that and you could try and kind of capitalize on that then like from a communication standpoint a little bit with her, but unfortunately then she ended up actually going downhill to more of a vegetative state later on, but she’s one I can think of where it was like pointing with something. She would inconsistently do. It was a command she would inconsistently follow.

I: So let's talk about the decline for a second. Do you remember her decline and what that looked like? Can you tell me what that looked like?

R: Oh so that inconsistent command follow was just gone and that was the biggest thing that we noticed. There was, when she first came in to us she would inconsistently do certain things for you like point, she would look at objects for you again somewhat inconsistently. She definitely fatigued pretty easily so. When she first came in it was, she would do certain things, sometimes for you, but then she fatigue and kind of fall asleep. I don't remember the exact time frame that it happened over, but there were some medical events. She had seizure and it was just kind of gone. She didn't, all of sudden she wasn't following any commands at all. She wouldn't look at things for you. She wouldn't point to things. She had some volitional movement. I think it was in her right upper extremity and so you could try and kind of direct her to use her right upper extremity for some purposeful tasks and she again it was just gone. She just would longer do anything with that right upper extremity for you. So that was the biggest, biggest thing that declined. I think the spasticity, had gotten a little worse too kind of over the course of that. The biggest thing with her decline was that total lack of any command follow or participation and any kind of purposeful tasks anymore.

I: Anything else besides the seizure that preceded that decline?

R: There were a lot of medication changes happening around that time frame, really from the first point that she came in. So I know the medical team definitely attributed it to her seizure. Family felt strongly that the medication changes were related to some of the decline that had gone on so, but those were really the main things that I remember around the time of that decline in her function.

I: So how did you communicate this with the family?

R: Not effectively is the short answer. You know, some of the initial communication would be around the idea of, “Hey, these people’s performance can fluctuate a lot and so you know the initial days that you’re noting some of this decline. It’s like well maybe something is just off today. We expect fluctuation in this patient population. Fluctuation is the norm. We don’t expect consistent performance, that’s sort of the definition of being in a minimally conscious state. So we’re happening to see less participation right now and we expect that, that will continue to fluctuate.” You know beyond that when it started to be more consistent, it was just trying to more, kind of say talk to the family about we have to try the things that we can get her to participate in at this point. We need to try and stimulate her level of alertness in any way we can. I don’t really know why this is happening, but just trying to talk to them about how we needed to change our approach and the activities we were working on, but the hard thing was is that you know the family at that point was coping very poorly. So their ability to, they were very upset and very disheartened by this decline. They absolutely recognized it too and really were wanting answers. And there wasn’t a clear answer to give. So, just talking to them about how we’re just gonna keep trying to kind of address the things that she used to be able to do and see if we can engage her in those ways again.

I: So do you remember or if you can give me an example of some of the activities you were doing with her at the beginning and then how those changed?

R: I mean in the beginning, it was in a lot of ways, I mean in some ways they didn’t change that much because from a physical perspective she was very low level to start out with either way. So I mean the types of activities we were working on with her were things like static sitting balance, working on head control, upright tolerance, and positioning in the wheelchair and then caregiver

training like we would do with any patient in that kind of cognitive status and physical status. So in some ways, even in the context of her decline, we were still working on those same things, but it was decrease in the complexity of those activities. So early on we could be working on sitting balance, but we might be working on engaging in some sort of a reaching task while working on static sitting, so almost kind of moving maybe towards some amount of dynamic sitting balance versus later on it really was pure static sitting without being able to add any of those additional tasks to that sitting balance task. Early on we would have been working on, positioning the wheelchair, but then again maybe her doing some different tasks with that right upper extremity or moving your head around and attending to different tasks when she was well positioned in the chair versus later on then it became really that it was just about can we position her effectively in the chair and again not being able to add that complexity of trying to engage her in any sort of meaningful activity while she was in the chair.

I: Anything else about this patient that you want to share? About decline? First stay?

R: Umm, no. Nothing that I can think about.

I: Let's move on and talk about your most memorable patient?

R: Like from a minimally conscious standpoint? Trying to think if [patient name] was minimally conscious when she came in. Yeah, oh she totally was in the beginning. Yes. So [patient name] is by far my most memorable patient. So tell me what she looked like when she first came in.

I: So tell me what she looked like when she first came in.

R: I mean she was small. She was just thin. She was a young girl and so she was just very thin. She'd had a craniectomy so she was in a helmet. So half her head shaved.

I: Can you tell me what a craniectomy is?

R: Yeah, so they removed a portion of her skull to alleviate pressure and swelling within the brain due to injury, so she had the helmet and from the beginning she was all decked out in when she came into rehab and her family was very involved and so they had multiple personal belongings there in the room. She was like into bright colors so they had all kinds fun pink sweat suits and like coordinated outfits for her. Multiple like soft blankets and like stuffed animals and just lots and lots of pictures in the room. So, and at that point, like from a physical perspective, she was total assist for everything and not following commands, but also pretty restless right from the beginning, so she was moving around a lot and I can’t remember which side was…I want to say it was her right side was hemiparetic, but the left side was pretty restless right from the beginning which from perspective always seemed like a good sign, like restless patients always seem to do a little bit than the ones who aren’t doing anything at all.

I: Why is that?

R: I don’t know. I don’t know if there is something with the just the neurologic pathways just being more intact, that there’s that motor control piece is there. So they maintain some of their muscle mass to a certain degree in some ways you hope that you can channel some of that restlessness into purposeful movement at least, so that there’s some movement there already so you can try channel into a purposeful task in some ways, but versus the patient who isn’t moving at all it’s hard to engage that or get that initiation of movement was a harder task to get out of a patient I felt like.

I: So were you able to do any of that with her? Were you able to use her restlessness to your advantage?

R: I don't know in the beginning if we really used it to our advantage or if more we needed to sort of wrangle it in, in the beginning. So, you know similar to the other patient I talked about you know initial types of tasks we were working on were things like sitting balance and positioning in the wheelchair and so the restless patient is just really tough to contain with some of those tasks because they don't have any sort of dynamic stability and so you're trying to like kind of build in and contain this sort of dynamic stability so, but the good thing is that she would do things like pick up her head. She would put her arm down inconsistently and intermittently and not always in a helpful was, but so I guess yes in some ways we could kind of build upon that because at least it was happening randomly and you could try and reinforce and get positive feedback when those kind of random occurrences would happen.

I: So typically when we think about a patient who is vegetative or minimally conscious we're thinking about those patients who aren't moving and aren't doing a lot and you're talking about her restlessness. So how did you know that she was minimally conscious?

R: Because even though she was restless it wasn't purposeful. She didn't use her extremities in any sort of purposeful way. You know she wouldn't take object. She wouldn't use any sort of an object appropriately. It was really very random restlessness. This isn't the right analogy, but almost in the way young infants just sort of flail their arms about. There's just no purposeful connection to the movement at all and it wasn't you know she wouldn't reach for family members with her purposeful movement. She wouldn't try and avoid things with the restlessness. It was really was just this random restlessness happening.

I: And then, how did she change. So overtime, the restlessness became more goal directed. It did get more purposeful overtime. She became more kind of alert and aware of family members and familiar people. She did get to the point of where you could engage her in just more purposeful

tasks so. Like overtime we got to the point where we could try things like standing and it was still kind of limited by the restlessness like she would kind of assist for brief moments and then stop assisting because it still that sort of inconsistent pattern of activation and understanding what you were trying to engage her in, but she would assist for brief periods of time. So when we were sitting, she would more kind of purposefully orient herself against gravity. You could get her to look at different people and look at different objects and then try and use that to engage her to participate in the tasks that you wanted. So like if you were working on sitting balance and you needed her to lift her head up, you know, you could look at a family member and get that motor output that you were looking for by engaging her in something you've asked her to do or getting her attention on something she was interested in.

I: So at that point would you say that she was still minimally conscious or would you think?

R: Well, that would be hard to say. I mean there was a point where it tipped over into she was fully conscious because it was, she was doing that stuff consistently. I'm trying to remember because she had her craniotomy at some point where she had a skull flap put back on and I think she kind of had a little bit of a set back at that point. And then I think it was kind of after that craniotomy then that she really maybe truly emerged into fully consciousness if I'm remembering correctly, but I think she was definitely an example of somebody who had got through blurry for a period of time about where are we at in the spectrum about you know is she consistent? Is she truly consistent? Like 100% consistent? Or is she still inconsistent enough where you'd say she was technically still minimally conscious? Or had she truly emerged into that conscious state?

I: So how do you navigate that?

R: Making that decision?

I: Mmmhhmm.

R: I guess I would say honestly at that point my level of experience and exposure to that patient population probably, I don't know how hard I was really trying to make a clear distinction at that point. There were a lot of other factors in her case that were dictating things like length of stay and therapy goals and not the therapy goals, I shouldn't say, but kind of what our long term approach was going to be with her. So I don't know that I was even that concerned honestly with where that fine line was and how that was impacting her. Although we were having a lot of discussions about prognosis for her and what her expectations were improvement. So I guess I would say around that time, I would've been trying to use some of that distinction and that information to think about what her long term prognosis was and at that point my perspective was that the long term prognosis was that she was going to need significant physical assist. So I think from that perspective I was still not necessarily projecting her to be in a fully conscious state and didn't know that she would achieve that. Did I answer your question?

I: Yeah and it's interesting because I feel like we're always very concerned about labeling people one way or the other. And you're saying as far as you in this case, that wasn't important.

R: It wasn't. I don't think it was.

I: So tell me why that wasn't important.

R: Yeah, that's a good question. I think some of that was purely of my level of experience that I maybe did not know enough to place value in that distinction, but it also at the time with her physical deficits being what they were. They were more significant, it was more significant for the burden of care compared to their cognitive deficits and because her family recognized her

physical deficits as being more of an impairment. Her cognitive deficits were significant and severe, but her family did not recognize that as a major problem. Can you give me some examples of her physical and her cognitive deficits. So from a physical perspective she was total assist for everything. The family was very hopeful that she would walk again and in my clinical judgement at the time I didn't think she would. She had very little movement on one side of her body. So on the right side if I'm remembering correctly but so from my perspective she was not going to be ambulatory and was more likely going to need use the wheelchair as her primary mode of locomotion probably indefinitely at that time from a cognitive perspective, she had zero safety awareness. She was communicating, but in a limited way at that point. She wasn't fully verbal, although I think she was verbalizing at that point. She wasn't oriented at all. Trying to remember if she was on a diet at that point or not and I don't distinctly remember, but from the family's perspective. She was {PT NAME} she was still {PT NAME} the person that she was. They felt like she was communicating effectively and they felt like they had a connection with her and were communicating with her. So from that perspective it was kind of a non factor to them and like the physical aspect was really the factor to them. So for her that distinction of minimally conscious versus fully conscious didn't seem to place into as much as like what the long term plan was going to be for care and prognosis and expectation for her because the family was sort okay with the cognitive status. I don't think they really, at least at that time, truly recognized the severity of her cognitive deficits. It was more about the physical piece to them and that was sort of the piece in some ways that I was in charge from the physical therapy side of things. Even though we know the cognitive aspects impacts all of that. So whether or not she made significant cognitive gains kind of didn't matter to them at that point because from their perspective she was who she was. So it didn't seem as important to make that distinction because

it was really all about the physical care that she was gonna need and the physical deficits that were going to be present because it seemed like her motor control was just so impaired and there was just not going to be a significant improvement in that. Did that answer your question?

I: Yeah, so then she does actually end up becoming fully conscious?

R: Yep.

I: And at what did you know, okay yes, she's fully conscious?

R: I don't know if I could remember like the switch or flip or anything, but there was a point where she was fully communicating in a very clear and straightforward way where she was very purposeful about her participation or lack of participation in activities. So those were, that was definitely the time where it was and I guess where I could get her to follow my instructions for the physical tasks that I was trying to get her to participate in. So we were still kind of plugging along this path of can she be ambulatory or not and it required a lot of work and a lot of physical assists, but they was a point where you could kind of tell her what you needed her to do and she couldn't always do it because of the motor impairments, but yet she was attempting. So at that point she was definitely clearly conscious.

I: And communication with family?

R: Was challenging because the family really recognized or didn't recognize the family's perspective on her was that she was her. She was fully {PT NAME} she was fully cognitively intact, they I mean they weren't, they knew she wasn't, that there were problems and deficits. They weren't confused about the fact that she needed 24 hour support from a cognitive status, but they really identified her as the person she always was at that point even though she wasn't 100% communicating she wasn't communicating in the exact normal way she had before. They

recognized her as her still and they were not willing to accept anything less than 100% and they were incredibly optimistic which as I look back on, to their credit, I think probably helped her along the way, but it was challenging at the time because from a clinical perspective, we felt like we needed to prepare them for the fact that their daughter was not going to return to 100% full capacity compared to where she was at prior to her injury. So there were some heated conversations. It was a workman's comp case so that added in some layers of complexity because you know workman's comp is really looking to us to make a distinction about what was the most appropriate thing and what was the best recommendation for her and yet the family was not willing to accept anything less than 100% at the time. So there was definitely some animosity in the situation between what family expectations and what we were saying as a clinical team about what reasonable expectation were. And yet even in the context of that they were just nice people and so even though they disagreed with us and about our recommendations and a lot about her what her actual outcome was going to be. They were always willing to engage with us in a mostly in a reasonable way and in a positive environment and really wanted just be engaged and helpful in anything that had to do with her care, so even though there was kind of this contentious disagreement about what the ultimate outcome was likely to be, they were just really thankful and wanted to participate and thankful for the care you were providing. It was kind of an interesting mix of being at odds with somebody and yet being able to work with them in a tough situation.

I: Anything else about her?

R: No, it's just ironic to see where she came because she made all kinds of gains. She's really doing well from a physical perspective much beyond my initial expectations were. So, it was actually a really good learning case for me because I thought I knew a lot at that point in my

career and it was a good reminder to me of the things we don't always know. And I think a really great example that really drove home to me in the context of severe brain injury how the trajectory for recovery can really be much longer than I sort of initially thought in my understanding at the time was.

I: So what did she look like when she left?

R: When she left she was still not walking, in any kind of functional household capacity. So she was using a wheelchair for primary mode of locomotion. She was doing more. She was certainly doing better with assisted walking when she left, but she wasn't going to be able to do anything like stairs and that was kind of the big issue because her bedroom in her home was on the second story. So, they installed the chair lift which was my biggest concern because she was still pretty restless in her safety, awareness was poor and her motor control was still poor. So I was very concerned about the safety of getting her up and down those stairs every day. But her family was in it the whole way so. I don't think I gave them enough credit at the time either about what they were capable of because they were capable probably a lot more than I recognized. So they made it work even though, she had to have two wheelchairs. She had a wheelchair downstairs. She had a wheelchair upstairs. I think she was in, I think she left being able to use a manual standard chair part of time and a manual tilt part of the time too. So she made enough gains that she could kind of sit with some amount of safety in a manual chair and I think we were even working on manual wheelchair mobility with her by the time she left.

I: Okay, we're going to do one more.

R: Okay.

I: Tell me about a time about a frustrating experience.

R: I can tell you my most frustrating experience and like the worst case ever that I would say maybe is what ultimately burned me out and probably led to me being more open to leaving brain injury as a full time status, but it was the same patient who had a significant decline. This is past the point of when the decline had happened. And the mother was the primary caregiver and it was a tough situation. I don't know where the father was. I don't remember the details of that, but it was basically mom was the primary caregiver. They were from out of town. They were from Florida, so she had zero social support at all here with her. She was also the…the patient had a child as well. I can't remember if it was a son or a daughter. So grandma was essentially going to be the primary caregiver now for her daughter and then her grandchild. So she had a lot on her plate for sure. She was there by herself and in fairness her daughter had come to us looking much more minimally conscious and by the time events sort of ensued you know she was definitely much more in a vegetative state. So it was a rough situation for her from a coping standpoint, but one of the big things was we were getting to the point where we were talking about discharge and mom was trying to figure out how to take her home. And I think they had gotten like a medical helicopter or medevac to get her up from Florida to us and maybe they paid out of pocket for that. I'm not sure, but so they were getting to the point of going home and she was wanting to fly her home commercially and at this point we're talking about a completely dependent individual who is in a what I would have called at the time, a vegetative state. And we were very concerned about the feasibleness of this plan, especially considering since mom was going to do this by herself. We were using a hoyer to transfer her. So nursing was using a hoyer, so I take that back. I, in therapy, was doing total assists dependent like squat, pivot, transfers with her to continue to try and assess whether or not she could participate in transfers, but it wasn't the kind of thing that I would ever have recommended for ongoing, to be like the

main transfer at home. So mom wanted to take her home and she wanted take her to another facility back in Florida and she wanted to fly her commercially. I was trying to figure out how that was gonna work and I didn't want her to do this transfer because I didn't think it was safe and I didn't know enough about the set up or the how you would kind of manage that when you are flying commercially at the time. So I can very distinctly remember she was working with another PT in our therapy gym and he had asked me in advance like what do you want me to do with them today. I said could you work on hoyer transfer training because that wasn't something we had addressed much yet. She might have even been initially like a max assist transfer and then after this decline it was really a total. So anyways, we hadn't addressed hoyer transfer training, so I had asked him to work on that with mom in the gym and I was in the gym working with another patient and she kind of refused to work on it with him and I was in the gym at the time and I was like trying to talk to her and be like hey you know just try this with him because what I was trying to do I reached out to some different contacts within RIC and also gotten some different contacts with some major airlines to try and find out more, like how would you manage this like what kind of assistance would she have. How would the transportation work? So I was trying to gather information about these things so that I could actually talk to her and really come up with a plan for could she do this transfer or not. Like would she have the help or not and so she was trying to talk to me about this, but I was working with another patient and she was working with another PT and I kind of said you know I'll talk to you about it later and I think her perception of that was that I was brushing her off like I'm not going to deal with that with you and then within two or three days of that we had family meeting scheduled to talk about discharge planning and our recommendations and she really wasn't accepting kind of where the patient was at at this point. So it was really supposed to be a time for the team to meet with her

and communicate and there were all kinds of red flags going into this meeting. She was demanding to talk to different supervisors and other people up the chain, but then was saying fine I'll just talk to everyone in this meeting and in that meeting she just exploded and lit into me. Basically, kind of attacked me personally, saying that I was not allowing her to care for her daughter, that I was dismissing her as a woman or being old and not being able to provide this care and basically that I was refusing to teach her anything that she needed to do with her daughter to take her home. It was an incredibly frustrating experience because what she didn't recognize was that in the background I was actually trying to do all of these things for her, so it was incredibly frustrating and she was pretty personal in her attack which made it frustrating too because as healthcare providers we go to work every day and we try and help people and we're really trying to do good and to sort of have someone very personally attack you and say that you are really doing the opposite of that was really frustrating because I'd worked with this patient for a long time. I'd worked really hard with this patient. I had been frustrated about the fact that she had gone backwards too. So, to really be personally attacked like that was like really upsetting to me and really frustrating. I'd gone out of my way and I guess I hadn't talked to her enough about it, but I had gone out of my way and talked to a lot of different people to try and figure out what can we do to help this woman fly commercially. She made it clear it was kind of a financial issue and so you know you can empathize with that. So, I really was trying to go out of my way and it felt, now you're just kind of kicking me in the face for all this hard work I've done and yet you aren't recognizing what the different barriers are and how hard I am trying to help you. So, it was super frustrating with how personal she was with the attack and how aggressive she was with it. And yet, didn't want anyone else involved so it was like feeling kind of trapped in this situation of like there's no good outcome and I can't fix your daughter and I

know that's really what you're upset about, but trying to help you in the best way that I can and yet you're just like attacking me in your grief which I knew, but it was very frustrating to work that hard with somebody and have the family really um not like not express gratitude because you don't, in our work, you don't always expect that either, but not recognize that you have tried hard for them. So, and it was frustrating just because she was doing better when she came to us and then the fact that she left really having lost ground was frustrating so it was overall and incredibly frustrating case to work on. Yeah, it was super frustrating. That was probably the worst brain injury experience that I have was working with that patient.

I: So, what happened when she left?

R: I don't 100% know. My understanding was that she went down to neurorestorative down in Florida and we didn't hear much after that about what her functional outcome was or not. She did end up flying commercially, I believe. And she made it so. What I found out later was actually airlines are decently supportive of flying somebody who is dependent. I mean, I still, I mean it's not the kind of thing I would ever encourage somebody to do because it's still a lot of work, but they will assist lifting somebody in and out of an airplane seat. So I know they ended up flying commercially and I know they got down to neurorestorative and beyond that, that's all I know about what happened with them.

I: Anything else about her, her care or her case that you remember?

R: The biggest other thing with her case was that she had so much spasticity and so we were trying to manage it with different bracing and orthotics. And I just remember, oh my god it was just a nightmare because she four different splints we were trying to use with her, but she didn't really seem to tolerate them well, all at once. Mom, I can't remember if it was that she didn't

tolerate them well or if mom felt she didn’t tolerate them well, but so we had just this crazy complicated bracing schedule and we were trying to work really closely with nursing staff to make sure everybody understood of like how to get the braces on correctly and because mom was pretty concrete about her understanding of how things needed to happen and if you told her about it one way, it needed to be that one way, even though we knew there was some amount of flexibility in certain things. I just remember between nursing and OT and PT really we ultimately had to come up with a 24 hour schedule that literally hour by hour laid out everything that was going to happen in her day. So we really had to work closely with nursing and OT and everybody to get everybody kind of communicating and collaborating to make sure we were like addressing what the patient needed in a way that the mom could understand and feel like her daughter was being well cared for. So it was just a lot of work.

I: Can you describe what the spasticity look like? (3:22?)

R: So it was a lot of tonic postures, so I don’t remember exactly, but I want to say, I’m pretty sure her lowers went into an extension pattern and we, we had this like wild brace, where she had to wear gym shoes because I want to say she internally rotated so the lowers were in extension, I think an internal rotation and so we had this brace that had like a bar between two gym shoes essentially on her feet to keep her externally rotated, but it would be these sort of like sustained postures that she would get stuck in and was losing range of motion because of that. And it was very hard to position her and flex and change the position of her joints with her spasticity and how significant it was. It also I think had her head was, I think her cervical spine was flexed and rotated to one side which made positioning her in the wheelchair incredibly challenging. She would go into, I don’t really want to say spasms because it was really more of these like tone patterns where she would kind of slowly move into this like tonic posture where she would sort

of creep out of the position in her wheelchair and get stuck behind her headrest which you know admittedly did look awful and wasn't good for her, but her mom would get very distressed when we would find her in these positions and we would do our best to try and get her wheelchair set up in a way that would support her head and prevent this from happening, but she would flex forward her neck and so she could get around the headrest and she'd get stuck then behind it, but it was like sort of this very slow tonic muscle contraction that would happen, but then would be very rigid and hard to mover her out of.

I: Anything else about her or any other patients, anything I haven't asked you that you want to share about this population?

R: No, I don't know. I think the most fun thing with this population is when you do get those surprise moments, like the first time they do something like wow I really think that was real, like they did that because they wanted to because it made sense. Those were the fun ones. And when they would emerge it was such a cool think to see and like so hopeful with their families. And so kind of rewarding to see that like person come back, even though sometime the person didn't exactly come back, you know you would see personality changes, but it would be really fun to see them emerge and develop some of the skills again that their families were hopeful for. It can be really fun from that perspective. The ones who didn't were really, they were the hard ones. They were, It was just so tough because you hope for them to make gains and their families obviously hope for them to make gains and many of them just didn't at the end of the day.

I: Okay, you're all set. Thank you.

R: Yeah, you're welcome.

Interview 8 Transcription

I= Interviewer

R= Respondent

I: …so it's your choice to participate in the interview if I ask you a question you are not comfortable with you can tell me that and we can move on and you can always choose to end the interview, okay? Umm so is it okay with you if we get started?

R: Yes

I: Okay. Alright, so the first thing I want you to do I want you to think about a time you were surprised by the way a patient um performed when you initially saw them.

R: Like my very first eval with them

I: Your first encounter, yup, the first encounter so you walked into the room and the way they presented was surprising to you based on what you were expecting.

R: Umm I'd probably say it would be my first emerging consciousness patient

I: Okay

R: just because it was the first experience I had

I: yup

R: ummm the mother was bedside with him and he was laying in bed not responding to commands but his extremities were all kinda moving involuntarily. Umm and yeah just seeing how much motion I guess he was completing without really

I: Okay

R: being aware.

I: Okay, so how was that different from what you were expecting? When you walked in what were you expecting to see?

R: Umm, I think I was expecting to see just more or less movement I should say.

I: OK

R: Uh, I think the mother's response was appropriate and everything um but I guess my initial thought of an emerging consciousness patient was that they would just be laying in bed and um

I: OK

R: Uh, be relatively still.

I: Ok, so was there anything when you reviewed the chart before you went in or anything like that was there anything in the chart that made you think that that person wouldn't be moving around or anything or was it just your own personal expectation?

R: No, just my own personal expectation.

I: OK, alright. So then if you think about either this patient or another patient that you treated, can you think of a situation where throughout that whole duration of you treating them you were surprised by something that they, either how they presented or something that they did?

R: Yeah, I think that um a lot of times I'm the most surprised after coming back after a weekend.

I: Ok

R: Um, I’ve had a coupel patients that were in a Mcs state and then there wasone guy that Friday I left and he was completely dependent with a ceiling lift and then come Monday we were starting to ambulate and walk with him. So I think just the speed of that emergence

I: mmhmm

R: just kinda surprised me

I: right, right, okay. So tell me, give me some specific examples about the how he was on Friday with the ceiling lift and then how he was on Monday

R: So on Friday, he was he would occasionally smile to joke or anything like that at that point he hadn’t been up out of bed at all. He just transferred from another facility and he would help assist with like rolling he would reach for you on occasion. Sometimes he wouldn’t respond, his eyes were in and out on being closed and open. So it was really variable. Something I could get him to do and somethings there was no response to. And then when I cam on Friday he was much more alert in his interactions he was responding approp more appropriately, laughing and then also following more commands and then just his strength. And on Friday he couldn’t really follow the commands for a manual muscle test or anything formally. Then on Monday, he was following commands to roll over to sit up even. Wasn’t feeling lightheaded so then we progressed him to stand and then from standing he progressed to walk in his room a little bit.

I: Wow wow

R: Yeah, quite the transition.

I: So how long was it from the time on Friday that he wasn't doing anything to the time on Monday when he was walking in his room? How long of a period of that? Was that all on Monday?

R: Yup yup

I: Wow that's amazing

R: Mmhmmm.

I: Okay, now can you think about um a situation where you were treating patient like this and you felt stuck?

R: stuck in what sense

I: Um either you felt stuck that the person wasn't doing anything or maybe you felt stuck in your treatment approach or just like over time

R: Over time, yeah so I had a patient he was in our emerging consciousness program and he was making some gains but still wasn't consistently following commands so he was here for 12 weeks and for the majority of that time he was kinda at a similar level and um or it was just very variable. One day he seemed to be occasionally responding or doing things more consistently while other days he was doing nothing and was just getting through. So I think with him, that was probably the most prolonged patient I've had that has been in one state.

I: So can you give me some examples of things that he would do one day but maybe not another

R: Yeah, we would for example we would be laying down on the mat table and if you asked him to lift his butt up he would be able to do a bridging exercise and do like 5 to 10 of them in a row. Or if we had him in the tilt table he would actively grab a ball from us and throw it whereas the

next day you would ask him to do the same thing so things we have seen him do in the past and he either wouldn't do it or this is the same patient whos arms are moving all over the place and it just wasn't as purposeful in our sessions.

I: Umm, okay. Can you think of any other examples?

R: Ummm, I think like somedays he would be sitting if we sat edge of mat we'd do it sort of supported by a therapist behind him and he'd be more still. He'd still be fully supported um where as other days, he would be kind of posturing back and not not as actively engaged with the person in front of him.

I: Oh, okay

R: I'm trying to think what other things we had him doing. I thin a lot with him was more of the remaining still versus the posturing because even in the tilt table he would be kinda moving forward with his trunk quite a bit. Um yeah.

I: So when you were, I'm assuming that his family was involved in some way?

R: mmhmm

I: So when you were communicating those kind of inconsistent um responses to his family, how would you communicate that to them?

R: I mean I think in the moment because their family was always present in the session

I: Okay

R: So they got to see it day to day. So I think in the moment we would kinda validate where if he did do something new where he was following the command to lift his butt up off the mat. Um, we would tell them that that's progress and then also emphasize that we are looking for the

consistency of day to day. And so I think um, with them the big thing was if they didn't see it the enxt day they wyould try super hard to get him involved. Um

I: Okay

R: so kinda explaining to them the kinda I call it the rollercoaster rheab that is not always consistent depending on sleep, medication, all that kinda stuff.

I: Mmmhmm

R: Um, so yeah. Taking it kinda day by day, telling them what it is we are looking for in regards to command following the responses to different stimuli and what them specifically we gave them a copy of the test the CRSR and the DOCS scales that we look at and um kinda guided them because they've seen us do it so many times and just in a ways that they could kinda do it in their room when they had some downtime.

I: Okay, alright. Good. Okay, so, when you were feeling stuck, you know the inconsistent performance. Can you tell me what kinds of things like did you do things differently to try to get him to respond more consistently was there anything that you did differently to see

R: Yeah, I think one thing would be if we would since you work with them so frequently see which things they respond the best to. So if sitting edge of mat was getting difficult for a week because like he would posture into extension and everything we resorted more frequently to the tilt table to work on the upright posture um at some points we just kinda regrouped as a time like what other positions. So we would get home prone lying on his stomach to see if he reacted differently and that position. Um, sometimes if he wasn't really responding to our voice or our commads we would have his mom come in and try to get his family you know the familiar voices and faces more involved. Or get pictures from their room. So yeah, family related.

I: Yes, alrights. Now I want you to tell me about one of your most memorable experiences treating a patient with severe TBI that started out in a disordered state of consciousness.

R: I think one of the most memorable, um, was, one of my EC patients he I can't remember how long he was here but um, he was in the vegetative state the only thing we could get him to do was raise his arm. He was opening his eyes um and then as soon as we put him back down he closed his eyes and that was about all we could get from him.

I: Uhh.

R: So, one day he was very into music and um, just music in general and so he was kinda starting to say a word here and there and then we put on one of his favorite bands and I could see him mouthing the words.

I: Ohhhh cool

R: to the song. So I think that was such uh inspiring moment to

I: Right, right.

R: see that one he had the memory of the lyrics but two once we got him something he enjoyed and was familiar with he really picked up on it.

I: So once you saw that, what happened after that? Did

R: Then, we used that a lot during treatment sessions.

I: Absolutely

R: We were kind of able to talk to his family more. So, they were able to tell us what bands he was interested in and we'd pick out the ones that were even more of his favorites.

I: Sure

R: And then we'd have music on a lot when we were doing the tilt table or sitting and yeah I think that just kinda sparked that. And then throughout his whole treatment once he emerged we'd do more focused things like he played the ukulele so trying to strum that and

I: Yeah, okay. So you said when you started working with him he was in a vegetative state and he was doing ver little and then he did eventually emerge into full consciousness. SO can you um can you sort of describe that um

R: process

I: Can you describe that whole process

R: yeah, yeah. So when he first got here um, he came from the he was in the ICU for almost a month I think at another facility. He came here and right away we began the tilt table and sitting edge of mat and just general range of motion. Um, the tilt table with him was kind of a big thing cause he his blood pressures and vitals were kind of going all over the board when we would raise him up so sort of acclimating him to upright posture was key. Um, and then, doing sitting edge of mat we were really just trying to get him to open his eyes at that point his eyes were really closed. And then eventually as the days were going on, he held his head up more so sitting edge of mat we wwere fully supporting his trunk and that was even holding his head up so we were paying attention to how long he was able to do that or how long he was even able to last sitting. So doing the DOCS and the Coma recovery scale continued to kinda just slowly respond to different stimulus um while there was more noise or the tactile cues or responding to verbal commands like wiggle your toes.

I: Can you give me a specific example?

R: yeah

I: Pick one of the stimuli and how he responded?

R: I was going to say the first thing I think was when we put an ice cube on his big toe and he wiggled his toe and that was the first sign in his lower extremities that I remember. And then it wasn't it was a couple weeks after that probably, again it was a weekend I went home um and then ON Monday when I came back his family was like you have to see what he did. So they told him to kick his leg straight and then he was able to do a long arc quad and kick his leg straight. SO then once he would get that motor return we would practice like kicking in the sitting position and then everything and once he was following commands we would do laying down and more exercises to work on his strength that we would get assisting him when we needed to. Because his left side returned a lot later than his right side.

I: Okay

R: Uh, and then once he was alert enough to really follow commands better we practiced using the easy stand which is the sit to stand device and then just standing edge of mat with two people. Um, and then from there we progressed and went in to the parallel bars working on transfers and just consistency with the set up of a chair and to train his family with that. Um, and then that's when kind of rec therapy started bringing him outside to get more fresh air. He's a very big outdoorsy loves the water kinda guy. And then, once he we were kind walking in the parallel bars it took three people to walk him in the bars and then we'd um go down to two people. Go down to one person. We put him in the exo device which is the exoskeleton which is the walking device and then from there it was a lot of working on um we worked on his strength obviously and agility kind of drills to get his thought processing a little quicker and to work on

reciprocal patterns and working with his legs and arms. Um, and then his left arm was a little later to reutn as well so we did a lot of functional tasks with like picking up balls and tryuing to put it through a hoop to try to work on his accuracy.

I: So tell me what you mean by reciprocal patterning because I don't know

R: Yeah, so if he was. If his attention, he was really good at if you said toe tap, put a toe onto the and then do the other leg. He'd be good if you're oding one at a time but then if you work on just doing both at the same time but opposite directions, um, that's the best way to kind of get back to the walking. The walking is reciprocal patterning too.

I: So when he left here

R: Mmhmm

I: What was he doing

R: So he actually he was here for a long time. He is actually still here on P-TRIP, our poly transitional rehab program. Yup, so he is from out east um but his family wanted to kinda keep the care consistent since they'd been here so long. So he's downstairs kinda working on his independence with he lacks some initiation and took forever to get dressed and to eat. So he's trying to kinda work on reintegrating in those areas but also participating in rec therapy. He was involved in our kayaking program so he's still doing that. It's transitioned from the pool since the winter to outdoors and yeah.

I: So h'es doing really well. He's functioning with assistance.

R: Yup, yeah.

I: Okay, awesome. Um, okay now I want you to tell me if you can think of another it might be the same patient but can you tell me about your most rewarding experience?

R: It was probably the same patient.

I: Same, patient. Yeah. Okay, so then let's do a different one then?

R: Okay

I: Why don't you tell me about a time when you had a frustrating experience treating a patient at in this

R: yeah. I think um my first emerging consciousness patient was one that um did not fully emerge um he is the one I was telling you inconsistently kinda following brief commands on some days and then nothing on the majority of the days. So I think that was frustrating, kind of one trying to explain to the family um the expected outcomes because they had unrealistic, no matter what we said they were like "nope he's coming home. We're going to get uh a ceiling track and he's going to be walking with us." And I think that was the hardest as a team to kind of address that with family, um and I think its still difficult for that family I'd say but yeah.

I: Can you give me some examples of um how you navigated that situation with the family? Like things that you either showed them or communicated with them to help them

R: yeah, so um. I think that it was a lot of education about um caregiver health and risks and um in regards to transferring um. Cause they were persistent on once he gets home we're going to transfer him by ourselves instead of using a lift which was recommended. Um, so I think it was extensive amounts of that. Um, caregiver persistent on doing the pivot transfer so during our session we did try it figuring it'd be safer to do it here than while we had people to assist um.

And then educating her on lifting mechanics um especially when just doing general range of motion or rolling the amount of repetitive forces that that's going to cause and then talking in team rounds we had a lot of discussions about it. Family psychology was in there. Um, so they were able to give us some input on how they best respond and everything so education in regards to lifting mechanics, equipment available that we can issue them. Um, why certain things wouldn't be appropriate at this time. Um, yeah. That's the best I got.

I: Okay, so can you think, can you think of some behaviors that he may have exhibited during therapy when you were doing therapy with him that maybe were misinterpreted by the family? And can you describe uh maybe how they interpreted or the behavior and how they interpreted it and how you handled it?

R: Um, I think the biggest thing with them was at his prior facility they the therapist would have him up in a harness system walking in the treadmill with max assist of three therapists. Um, and when he came here we did not focus on walking at all because at that point he wasn't following commands it would have been very dangerous. Um, and so I think sitting down and explaining to them that that's more of a reflexive pattern whether than his intentional mind doing it. That took a lot of education which at first they were very receptive to and then I think as some of the weeks went along and they saw that he wasn't really changing they got more persistent on he needs to be standing, he needs to be walking. And again, having to reiterate that that's not the safest option and that there is not the carry over from day to day with him. Um, so I think that was really hard and then another thing would be anytime he did follow a command I think that gave him higher hopes than what we were sesing in the clinic. And I think re-iterating the consistency was kind of the key thing that we're looking for from day to day or like he would occasionally if you had like a comedy show thing on he would like smile and laugh. So I think that they got so

excited right away that that was something new that that was change, that that's emotion he was showing and that he's there and yeah. So those are some of the examples with him.

I: That's great. And then, um, can you think of any other patients that you've treated that were either um you know any of the situations that we've already talked about. Surprising or frustrating memorable.

R: Umm, thinking of one of my girls that was in the emerging consciousness program I think the biggest or most memorable moement for her with me was um when we were sitting edge of mat and we were asking her questions. She wasn't really talking much at this point but it was written in her notes that she wrote something and that she liked to draw and that she was writing on occasion at the last place. So with her we weren't really getting too much out of her so then we gave her paper and a pen and then we would ask her questions and she was able to write her name and then um when we asked her where she is she wrote Minnesota. And so that was really exciting that we could find a way that she could communicate. Um, rather than us just asking her questions and not being able to vocalize and stuff.

I: So did that then progress or did she kinda stay at that same level?

R: NO, it progressed. At first it was kind of an easy way to ask for yes no or orientation questions but it wasn't consistent so it gave us a better understanding of where she was at cognitively in regards to like if she had siblings like the little information that we knew and then so eventually we can kinda see gradual um changes in her answers being more accurate um the thing with her thought was that once she got used to more writing and once she could sorta talk she resorted to the writing so we had to find a way to sort of wean the writing off and make her

use her vocalizations. Um, so that was a difficult transition and I think it was a little frustrating for her at that time to. [laughter from I] SO yeah that's how we

I: Can you describe how she would exhibit that frustration or

R: Umm, I think at times when she would say something um and it wasn't not in context and if at any point we'd be confused or re-ask her like "Did you mean that?" She would just give us like a quizzical look like she didn't know what she said. And then um, she would try again and it still didn't make sense so I think anytime she got like uh try again kinda statement she'd get frustrated in a sensea nd it was mostly just facial expressions and that's kinda when she started talking a little less I think and kinda exhibiting that behavior.

I: Okay, alright. Um, Okay, so those are really all my questions but can you think of anything else about this population that you wish I would've asked you? Or that you um would like to share as far as you know how they change or um anything like that?

R: I'm trying to think. Not that I can think of off the top of my head.

I: Okay, you gave me some really good examples…

R: Okay

I: …and I liked your I liked um your reference to the rollercoaster of rehab because especially with this population I feel like that's the

R: Yeah. I think its interesting because there is a lot of throughout the whole process it's not just the constant incline ther eis a lot fo medical issues that come up for instance one of my patients her shunt was infected so she had to go to the ICU for a little while and hten coming back up its

like are we going to start at at where we were or are we declining and just seeing that progression come back or not at that point.

I: Right. Right right. Yeah, very good. Well that’s all I have. Thank you.

R: Thank you.

I: Thank you so much.

Clinical Interview 9

I: Interviewer

R: Responder

R: Just in general like what

I: Yep, you walked into the room when you were surprised how they presented, you were surprised by their trajectory, anything.

R: Oh sure, we had a young woman a couple years ago who had really intense dysautonomic symptoms. So, each we walked in a room and she was having really, increased heart rate and blood pressure and she's in a very abnormal position, posturing her were the only thing on the bed basically just her head and rest was off the bed.

I: So, what was surprising about that?

R: I think it's pretty…just that positioning…seeing somebody in such a kind of abnormal position. I think was…

I: So, that's not something that you would typically see with these patients in disordered consciousness?

R: No, I said I work in different roles in brain injury. It's the only time I've ever seen that. And just this life threatening symptom that she had. It just kind of came out of nowhere.

I: And then how did she progress from there? Did symptoms resolve? Did they get better? Did they get worse?

R: {Tape inaudible } of those symptoms throughout her course to leave we try some really invasive interventions and some medications and those did improve to the point where she could stay in the bed or a wheelchair.

I: Can you describe some of the interventions or give me examples of some of the medications that you used?

R: Well she required multiple trips to the ICU. I don't know exactly what all they did. What we did was like propanol or IV fluids. She responded to, I think she ended up getting a baclofen intrathecal pump that seemed appropriate…oxycodone and lorazepam and a lot of medications.

I: A lot of medications? Did she respond as you expected her to?

R: It's hard to know what to expect, so I don't know what we expected. We hoped she'd get better and she certainly didn't get better.

I: She did not improve?

R: I mean she improved from the symptoms, so she could go to a place where she could sit in a wheelchair.

I: So, she went from being in the bed, being abnormally postured to being able to be in a chair comfortably or positioned better in the bed. Is that what I'm understanding?

R: Yeah, I would not say comfortably because we have no way of knowing what kind of comfort she might have had, but to the point where she could sit in a chair with adaptations, but that was available and to be transferred. I think she could be transferred from the bed to the chair and from the chair to the bed when she would not have been able to with the type of posturing.

I: So, let's try and think about a different patient that you've had now, same situation, first year out of injury, disordered state of consciousness and tell me about your most memorable experience treating one of these patients?

R: A young man, a couple months ago, when I went into his room for the first time I was covering so I didn't know him well, but he was on his way to surgery for the procedure and he looked right at me and said bye. So, I thought he was awake and then after that everyone else I talked to said that he had, he was not doing anything purposefully or anything at all. I said, I pretty sure he said bye to me, like I'm pretty sure, and a few weeks later he did emerge and that was pretty interesting that he did have that one moment for me where he looked right at me and said something appropriate. So, that was interesting.

I: So, when you had talked to other people and you said that you had experienced this and they said we haven't seen this, he hasn't been doing any of this, how did you kind of navigate that situation?

R: Well, I kind of because then he went to surgery and it was a small procedure but I think the anesthesia did affect him. He was already so impaired and recovering from so much so then it did take him a little while to even stop being sedated from his surgery so I was kind of worrying more about those things and there was no prognosis and other discussions at that point because he was so acutely ill. I didn't really challenge the team, but I did say I think he said something to me and of they'd all known him for weeks and this was my first time with him, so a couple weeks later when he did emerge, I told you guys he said something, it was more of a, kind of a personal (??6:10).

I: Sure, did that change how other people started to treat him? Or did that change his treatment plan in anyway, do you think?

R: No, no because the team they're very senior people and they, they were positioning me as someone actually just coming into the situation to tell the family to stop, to tell them to just stop right there because he had such a prognosis that they were sure was a certain way…no I wouldn't have done that anyway just because I'm just coming into the situation, but certainly if he got sicker, that would have been what we would've done and I would have followed the team. Although, that wouldn't have happened because his family didnt want that.

I: I want you to tell me about one of your most rewarding experiences treating a patient with disordered consciousness.

R: I think working in the system here, it's been really great to have the ability to discuss these issues with other teams, so when we do have someone say I'll go back to that first young woman, she would go to ICU and then we would kind of educate the medical teams about what brain injury is and what disordered consciousness is, really opening their eyes to the fact that people can emerge and it's not

such a guarantee that it's a bad situation. I think that's been a nice ability to go around and talk about these issues in a way that is based on a patient, but that is these are a set of ethical issuesthat people have their own set of opinions about, but then when they actually meet someone they can seeit can kind of change how they think. It's a good thing to be a part of.

I: Can you give me some examples of how you describe the concept of emergence?

R: Well, we follow the instruments and so we can't really say they've emerged until the team says that at the meeting. I say they participant and consistency.

I: So, from a medical perspective. What kinds of things are you looking for when you're evaluating if somebody has emerged?

R: I'm just trying to see what kind of responses they have and of course we're in there, in our rounds for a couple minutes here or there. I try to watch when they're doing their…the clinical rescale, but I don't always get to see the whole thing. So, just trying to see them at different points in the day for a little bit here and there and seeing what they're doing, even of course doing different things. We have people who have something hooked up to their foot that they're doing and we have other people who are doing different things with their hands, eyes. Just trying to look for that something.

I: So, can you think of like a specific patient or a specific example. You said some patients will do things with their hands or their feet or their eyes, can you give me specific examples of things that you've seen.

R: Yeah, we have a gentleman here now who, his main things, he was tapping his left foot. It became clear that he was purposefully tapping his left foot and we could kind of harness that for communication. Seemed reliable to do that. So, when you first go in, you see boy he's really moving his foot a lot, but then realizing that was something we could use and we had other people who maybe that really wasn't what they were doing was a foot or a toe and then he's able to move it more movements that we could see, but with other people they have a little bit smaller movements we picked up like something of sounds or some type so when he move that toe or you can (10:55??)

I: So, with the foot tapping how did you know that it was purposeful?

R: Well in the beginning we didn't really know when you talk to the team and say he's tapping a lot. The nurses I think, nurses who spend more time with them kind of got that right away that seems to be, you know, if I do something that he seems to pleasurable like mouthcare or something then he'll stop tapping or he's tapping more when he's wet or something like that so, they kind of knew that connection right.

I: So, it sounds like a lot of these examples that you've been giving me are things that you've seen and you've also been talking to other members of the team. So, it seems like a pretty crucial piece to this puzzle is that communication. Can you talk to me a little bit about that communication?

R: Yeah, I think here we have good informal communication that we do all the time, you know when we see each other and then we have these formal rounds, weekly or every other week with other meetings kind of scheduled in….see patients so kind of have like a similar perspective that we all have and …some people who respond better than one person or another and make that we all know what we're doing with each other too..that's really important what I've done in other places we're not, certainly nothing

like this, but met people who are in these states and other places and we just kind of going on what you think or I think you need or I think this happened, butyou don't really have that sense (12:52??)

I: And you also had mentioned that one of these other patients, the CRS? Can you give me some examples of how you use that?

R: Well, usually they do it…I think it's every week most of the time they do that, they do that and then they also do the disorders of consciousness scale I think DOC and JFK…and they do them I think it's every week so PT and OT do mostly the first one and then speech also is involved and maybe it depends on what's going on, but it's about every week and then they give the scores…and I think it's important know the scores, it's important to know what they're doing. So, I don't actually do the tests myself, although I try to watch when I can. The cool thing Is that I think um, It's just a number you know and although it great numbering and really hard to get it is really still just that one point in time so the night nurses are saying, yeah but he does this I think if they could consider that how much probably don't do any of the actually formal tasks they tend to have really great insight into what's going on so. And then family sometimes they have ideas that don't match what we're seeing on our formal testing, but a lot times they're right too so trying to get everybody. I think it's our role to kind of take all that information and just know what's going and we're not making any set decision on it, but it's kind of the piece of our

I: So, the CRS specifically does that influence your decision-making in any way as far as your treatment plan, your discharge plan or?

R: Yeah, it does in that because you know there's cut off officially they've emerged earlier. That can change what we do in terms of how long they stay because if they have emerged of course I'm not going to base it on one point in time, But if they have emerged or it looks like they're improving on a scale, is that objective could extend their length of stay here to either see where things go or not, if that score stay low and it's consistently low and we're also not getting information from other people that's contradicting that thenprobably not extend their stay. Would it change what we do medication-wise? Possibly, if there are things that are really changing. We look for an infection and look for some reason why slowing down. if we do start a medication, it's good to have some objective evidence that helped or not and sometimes that could be objective evidence. It's often not sensitive enoughto pick those things up, but I think it can be helpful. The most thing…the tracking…is to keep track if they have, if they are getting better or get to the point where its time to do rehab after that. It all about just the way of seeing (???)

I: Yeah, great. So, anything else that you want to share with me about any of these patients that we've already talked about before we move on?

R: No, I think that's enough.

I: It's good? Okay. I want you to tell me about a time you had a frustrating experience with, either with the patient itself or with the family or with the case?

R: Yeah, You know one of the things I cover for when the inpatient attendings are outSo, now that's really what I do so a lot of time when people are here already and they've had sort of their relationships with the team and I kind of come in, which is always is really great because a new person for them and they tell me things I guess what I'm getting at is there's a young man, I think he was here last year, but he, his mother for whatever reason was really challenging for the staff. She was very, he was married

with four children…he must have been in his late 30’s I don’t know, but his mother was just became more personable when he got here and was mostly involved…the staff was spending like two nurses to get him out of bed, requiring a lot of assistance and his mother wanted to take him home alone and this kind of thing and so it was a frustrating in that we felt that his care was not going to be once it got to the point that he was going to go home with her, there was some frustration about is this gonna be safe and then I think for me the one day I got on and then she started telling me I was so bad. She was gonna take him home and all the stuff that was going to happen like that week. That was kind of frustrating because I just met him and maybe all this stuff, but then she wasn’t really telling me…It wasn’t exactly true because she didn’t have the house ready that week so at least my frustrations were I think I understand usually come from a place of hurt or whatever, but she was kind of trying to get me on her side and then it just it doesn’t work because we have such a good team and so trying to step back and be there with the whole team I think it went okay, it was the attempt to split which I always find difficult.

I: So, then it kind of come back to the whole team communication piece again?

R: Right, right team and making sure that we’re also…we don’t have the luxury of then saying oh she lied to us let’s cut her off or whatever right because she’s still gonna do so trying to figure out a way to have the compassion and appropriate response and um, also making sure that his needs are met and still not saying it’s a bigissue, but his you know, I guess especially myself who sort of comes by and assists without leaving, but I guess when people are that invested in that emotional about things she saw me as someone who would be different from the rest of the team….its a hard situation but it worked out within a day or two I pulled her back, but I with the team and I’m with you too but this is my team.

I: So, I want you to think about a time when you felt stuck with a patient.

R: I suppose that first young woman that I was talking about with the posturing. We were stuck for a little while before we could get that baclofen pump placed and mostly because the neurosurgery team, we kind of didn’t believe that she was a person who would be a good candidate for that so that they really wanted me to do some testing to really demonstrate that it was gonna help because they were kind of not really eager to put it in the first place and every time I tried catch her on the testing schedule probably 3 or 4 times and she got a UTI or she got sent back to the ICU. So, it probably took me 2 months just to get the test that they wanted and then they finally did put it down, that was kind of weird. Just between kind of waiting

I: Can you describe for me the posturing a little bit? Break it down layman’s terms what it looks like.

R: Well, the tip of the back of the head is on the bed and maybe the heels are on the bed And that’s really it so it’s kind of arched. Those are the worst ... I guess sometimes your elbows might have been on the bed too, but generally not, no cause her arms were in such a…her elbows were twisted out at her shoulder so that they couldn’t be on the bed either way, so it was really just her elbows I mean just the back of her heel and the back of head.

I: And then after the pump and after the interventions, what did she look like?

R: You know much smoother, she would still have abnormal posture like her arms where It would still go out alot…her tone was pretty high in her hands, but and her feet were still, you know, like her toes were pointed up in a flexion position but she would at least be able to at her hips and at her core to be flexed

so she could sit up get on the edge of the bed. And she wasn't constantly in that posture it was these parts [responder pointing to body part??] and it was her back there were certain times it was worse.

I: Can you give me some examples?

R: Like initially when she first came, well a couple of weeks after she got here and then a couple times when she had infections in between Seemed like when she was involved in a, likeher family would fight in the room andshe would get a lot worse too

I: Interesting.

R:[mumbling]

I: So, you think she was aware of what was going on around her then or?

R: We didn't know. We didn't know. She would giggle a lot and then she would cry a lot and she would Sometimes her heart rate would go up

I: So, were her emotional responses appropriate or random?

R: Well, we didn't spend a lot of time trying to figure that out because??? Exactly, um, nurses really thought that she was responding to certain things they did or certain music they'd put on and she would cry or laugh. Her family certainly thought she laughed more at things she used to think were funny. She just like sort of start laughing. We tried to give her medications for that

I: Sure.

R: I mean her family liked better if she would laugh, so, I think we kind of started titrating the medications to stop the crying if we could, but I don't know it didn't seem to make a differene

I: Okay.

R: But I wasn't in all the time.

I: Yeah, so when you have families that are extremely involved and they're with the patient all the time and they are seeing things that you're maybe not seeing….how do you take that information…then take the information that you have and work that into your treatment?

R: Yeah, it's really interesting one right[incoherent mumbling] His wife would say that even if she wasn't here that she could hear him talking to her and she got here she would hear him talking to her and mom would say he moved I saw him move when I asked him to move and no one else saw it, you know, but they were really that connected with him. It's hard because you don't want to say, like, no way this is happening, but you know you don't want to also we just want to take that information and then talk with the family about it. I like to just talk to them more about what is it you seeing and do it outside of the room and most them the patient doesn't necessarily hear that and that's an issureand family would think they hear that and then we just talk to them, this is what we're seeing, in a really kind way, and a you know betterwe always say if their gonna do something their gonna do it for you, you have that connection that none of us have and then as we get to know them better, we have that connection too, a little bit and now this is what we're seeing. I just think it's really important to be on their side no matter what. For us all to be on the same side for us to feel like the family is with us because it's not us against them in a program I mean there are times when we have to do that right. This is what I see, this

is what's happening, but also I think some much is unknown I'm not talking about a complete spinal cord. I talking about brain injury who knowns what's going to happen, you don't. So, we don't have data. We don't know. We're still learning. You just kind of take it. I think sometimes when we have these clashes or when nurses say oh she's making this up or she's lying. The therapists get upset, so I think just my personality or the way, plus I'm only covering and you know I'm just covering for a couple weeks I just try to take everyone's side, Yeah, I believe you, yeah, it's hard to work with this family or I know it's hard to ??? the family says I hate that speech therapist cause she hates me well the speech therapist does hate her I've seen that and I know what she's saying. So, just trying to sort kind of calm everyone down and empower everyone because I need the wife to be watching and I need the speech therapists to be at their best so I'm not trying to put anybody down, but also I mean our therapists have been here a long time, but Ive been working with brain injury a long time you can really be surprised at the things that should never happen, that happen good and bad which are way better when it happens so I don't want to tell the family member that what they're saying is impossible, but its in point…I use words like we have to hope for the best and I'm going to be prepare for the worst because I have to do that, I have to know where he's gonna go, if he's going to be in a nursing home, I have to get that ready. I know you don't want that, I get that hope and do everything you can over there and we're gonna something what we have to do over here. Do you mind if I get this really quick?

I: No go ahead. (End of interview part 1)

I: So, you just mentioned about patients that you don't expect something to happen and then all of a sudden it happens. Can you give me an example of something that you've seen like that?

R: We had, you're sitting there, you're on rounds I mean you're walking on someone's bedside and they wave to you. That they haven't done anything, their scores have been really low they haven't done anything and they just like emerge right then. How did that happen? It just happened. They start waving and then they start talking. Generally, it's a little more gradual than that, but it's back to who hasn't been doing anything all of sudden starts doing something. I've got other people not here, but where they've had a big, a crazy ischemic area on their scan that goes away, just goes away. Where did it go? How does that possibly happen, but it happens, you know so, it doesn't happen much, but it does happen until we know more we gotta keep our minds open.

I: Anything else that I haven't asked you about this patient population or the work that you do with these patients that you want share?

R: Maybe just how hard the team works, you know, in terms of like the skin care and the positioning and the mouthcare and the, I mean there are people on our unit who don't get pneumonia, they don't get tubfeeds never get clogged they always have good bowel and bladder programs in a way that we have nurses and they're able to keep all these bodily functions in such great shape all the time. It's amazing. I think also like our way that we support the families is pretty amazing sort of.

I: And how important is that the basic care stuff the oral care, bowel and bladder, supporting the families, how important is that for rehab?

R: Without that, they patients would all die. I mean they would just die. They would get a UTI and they would all die which is what happens when they got to nursing homes. They didn't get good trach care, the trach gets clogged, they would die. It's not, I don't think there's any question. It doesn't matter but I

was an ICU nurse for 10 years and working in trauma, these nurses work as hard as the same nurse on those things if not harder and they have more patients and its day in and day out you know you have someone who's heart rate is through the roof and they have to get IV fluids and your managing of IV fluids and the tubes, you know I think especially with the bowel stuff, you know, it can be so hard to get to turn over to get them on a good bowel program the tube feedings they have it all to [incoherent mumbling]

I: Okay, great. Anything else that you want to share?

R: No.

Clinician Interview 10 (NOT VERBATIM – QUICK SUMMARY)

I: Interviewer

R: Responder

I: {Read standard instruction/consent}

R: Yes

I: think about DOC patients, tell me about rewarding experience

R: Taken care of plenty of DOC patients, having a patient unable to participate, as they continue to slowly emerge, just little minute things seem so huge, having them actually fully wake up and start participating in therapy, how rewarding for their family, often have other ortho injuries that affect their course

I: you talked about little changes, give me examples

R: opening eyes, thumbs up, moving parts of body, that nonverbal stuff, eyes lightened up, like they are laughing to things you are saying but their body isn't always following along, sometimes you can't quantify if they are in there necessarily, but they are showing personality without showing personality, they don't have to talk to radiate

I: can you think of one specific patient who started showing some of these things and talk to me about him or her

R: Young man, injured overseas, blast injury, shrapnel, ortho injuries, started doing subtle things like thumbs up, wasn't able to talk or see, sporadic with movements, he was here for a significant course, he was very young, active family, a lot of friends, continued to progress to waling, minimal talking, comes back to visit, lives with his dad, able to talk some now, to see that he is still able to live somewhat of a new norm, happy life, goes out with wounded warriors guys, has support adjusting to new norm.

I: Important of new normal is important, can you elaborate what that term means

R: often devastating injuries in this population, sometimes new normal is dealing with pain issues, family dynamics, may have to quit jobs and become caregiver which was not anticipated falls into new normalcy, now relying on caregiver or assistive technology, they were independent but now not independently able to do things and have to rely on assistance whether a device or a person, adjusting to needing a handicap vehicle or handicap accessible house, changes what you have to make work for a new lifestyle.

I: want to go back to the pervious gentleman who was blind and comes back, if you can remember what he looked like when he came in and take me through his progression to when you discharged him

R: he didn't do much of anything when he came in, had significant wounds from surgeries, pale and weak just from the traumatic events, he didn't much of anything for a good period of time, but as he started to, he had a peg, trach, craniectomy, wounds, his face was partially, he was missing part of his brow, he didn't do much and pretty much was dependent on staff, required special wheelchair to

support his body, unable to hold his head up. With continued time in therapy he started to progress, his medical status stabilized his respiratory status, secretions stared to decrease, started to tolerate feeding better, seemed like once a lot of acute medical stuff started to stabilize he started to progress, little things like moving fingers, started to do subtle things, unclear if it was in response to things with time continued to show consistent responses.

I: Can you give me some examples

R: Thumbs up and thumbs down to answer questions. Determined over time he had significant aphasia, still to this day has limited verbal output, but spontaneous phases now. At the time unsure if related to the trach or . As he continued to progress standing frame transfers, to stand pivot transfers to walking. Progressing diet from tube feeding to oral intake, progress in take diet. One of his sides was pretty affected with weakness and tone. We determined over time with further testing, specialized neuro testing he was blind. Saw shadows if anything, could not rely on vision during therapy, required tactile and hand over hand assist. Discharged home with family, able to walk with assistance, one person holding on to him, required a left AFO, gets botox to arm and leg, splinting, but able to walk.

I: Anything else about this patient

R: No, we've been lucky enough to have him come back for 2 week short stay, reassess, caregiver needs, specialty appointments, dietary follow up to get these things worked on. Some things plateaued some progress, about 10 years later

I: This is an inpatient stay for a couple weeks?

R: For a couple weeks

I: How often

R: Once a year, maybe less. The particular stay needs specific goals for inpatient rehab. About once a year. Yearly re-eval. Usually coordinated with outpatient team to coordinate outpatient appointments to decrease burden on caregiver as well.

I: Change gears, tell me about a frustrating experience

R: really severely injured, young, young wife, 2 young kids, sent here from another state, from Walter Reed, he had, being treated with hard core antibiotics, he had C-diff, he came with C-diff, it was very very challenging because therapy every time we moved him he would be incontinent. Trying to clean him up and keep his skin good. It was frustrating for the family because they wanted him in therapy but he was missing therapy for reasons out of our control. He had reacted to medication and got Red man syndrome. They had gone to dinner and the med had started before they left and they were just starting to get comfortable leaning away and he reacted. It felt like a setback. There was a lot of blame, not necessarily appropriate. We were getting the brunt of it. The family dynamic can be very frustrating, you get the inappropriate blame piece, you try to communicate as much as possible. They try to split providers and the manipulations that occur. Not all families are stable then you add this severe stress. It can be very difficult. There is potential for compassion fatigue, it can be tough at times, its important to identify those things and how to take care of yourself. With that particular case, I've seen similar, but with a different particular thing.

I: How does that impact what you are able to do to meet rehab goals and progressing the patient?

R: Sometimes they get in the way, if they didn't spend so much time telling us what we need to do. We are still doing what we need to do. The therapists are really good about managing the patient, working with the patient, and provider, and team to work on what is best for the patient. When the family tries to tear us down we have a consistent response. Communication is necessary.

I: Can you describe C-diff for the non-clinicians.

R: C-diff is a toxin in your colon, antibiotics will, the good bacteria, so its normally in your colon, the antibiotics break down the good bacteria, releases toxin, causes significant diarrhea, inflammation in the colon, it can get so bad it causes bleeding in the colon, mainly causes diarrhea, considered an infection, Good bacteria is now bad and releases toxins. There is no warning for the diarrhea. Few different treatment options, it takes time, 6 weeks or longer to recover. It weakens them, they feel ill, can cause nausea and vomiting. It really affects the patient.

I: You mentioned Red man syndrome. Can you describe that

R: He was on vancomycin. Basically, like an adverse reaction to the particular medication. Cause a full rash, face is bright red, itchy, inflammatory response to the med. Itchy and red. For a patient who can't talk or communicate it's a big deal. We caught it pretty early, but it's a matter of why is this happening.

I: With the family being manipulative and placing blame, how did you manage that?

R: We do a primary nursing model, we start in early stage on rehab unit. We were able to identify for the week ahead which nurse would have him, make sure there was always a primary nurse so the communication was consistent, just because it was difficult to be in there if you didn't know what the plan was. We were pretty lucky to have at least 3-5 nurses consistently with him on different shifts. Charge nurse would be checking in, we do a pretty good handoff.

I: anything else about this patient or case

R: Happy ending is they come back to visit and are appreciative and stay at the fisher house and cook meals and visit other families. They are ultimately thankful for what you did, its just in the moment.

I: What you say this patient had a good outcome in the end?

R: He had a good outcome still requires, he also ended up being blind, he can walk independently, living from what the family says a happy full life. Wife was unable to return to work, they do a lot of advocacy stuff and caregiver support stuff and veteran clinics for adaptive sports. I think they are making the most of their new norm which is nice to see.

I: Tell me about a time when you were surprised by one of these patients?
R: recently had a younger male veteran, had a pretty significant injury, bilateral craniectomy, peg, trach, dependent, neuro storming, so many medical things going on, and this kept going on and on and on, and we felt like we kept having to manage the medical pieces. From a team standpoint and the tests they do to track recovery, they were bad, prognostic indicators were really poor. We were constantly managing acute things. Things were very poor. The provider had a strong inkling because he had bilateral craniectomies, he had syndrome of trephined – pressure related thing. Hard to really say for sure, but she wanted to move forward with cranioplasty and once he had the cranioplasty he made almost a full

recovery. From being, it was still slow, but over the course of the next couple months he was able to walk, talk, and respond. Working on upper level cognition, he's back in college, planning to be a firefighter. To go from poor prognostic indicators to living a full life again.

I: did you have any indication this type of recovery would happen?

R: We always remain hopeful. With the doctor's concerns with the syndrome of trephined, I was slightly skeptical. I don't think recovery can happen until those things are stabilized, Hopeful but didn't expect him to do that well, to be able to return to full independence, I didn't expect that.

I: How did the recovery start, what did you see first?

R: started moving his hands, starting tracking, the storming started to decrease, respiratory stuff started to improve, more alertness of environment, arms were first then legs. Started to get more restless. Seemed like he started talking, it was slow, one word things. He would want to do things, you could tell by his body. He slowly got strong and stronger in therapies. Body just started working together. Just slowly gradually improved. Able to get off tube feeding which is a slow course as they do swallowing evals, no longer aspirating, eventually independent on the unit, balance continued to improve. From a cognition standpoint initially needed supervision, then able to manage own schedule, ask for meds, get dressed. Had to battle normalcy things like I want to stay in bed until 9 and not start therapy at 8 after breakfast. Slow and gradual but consistent as they medical pieces improved (got rid of trach, peg, cranioplasty).

I: do you remember what his length of stay was? What kind of timeframe are we talking about?

R: like 3 months before he had his cranioplasty I can't say for sure then maybe a few months after cranioplasty that he discharged, probably about 5 moths, our EC program is about 5 weeks so once they emerge a new clock starts and the team determines what we're thinking. I want to say at least 5 months maybe 6.

I: what happens if someone is admitted to the emerge consciousness program (EC) and they don't emerge? Do you see that often?

R: We do see it. Its…its hard. The acceptance, maybe they need more time. There is a lot of struggle for family too. As much as the team tries to give prognostic indicators, its not the greatest. Often then end up in a nursing home, sometimes family takes them home and I can even image. Nursing is 24 hours. For a family to care give for some of these patients that require trach suctioning and it's a lot. I can't imagine how they do it sometimes. But it does happen, it definitely does happen. Some patient will emerge but functionally they will not be independent again, whether its time from injury. Or they emerge but because all the other medical factors affect them like HO forms on the bones, or the bones are weak. It definitely does happen. Often times, the EC program they are the worst of the worst, but yeah it does happen.

I: Can you think of one of those patients and describe what he or she looked like?

R: Early on in my rehab, we had a young, he was probably 20 something, he was in a MVA, very debilited, very thin, mom was support system , not much ohther family. Mom wasn't here a lot, she worked. He was here for 12 weeks and didn't improve. At that time though he had a craniectomy and his flap was sunken. They waited typically 6 months after trauma for cranioplasty, I think the research

has changed since, but I think that affected his recovery. Years down the road we heard he did wake up but not in our care. After cranioplasty in subacute care, likely in a nursing home. He didn't wake up in our care. He needed potentially more time. It was tough. There was a lot of pleading, he needs more time. Yes, your right but it's difficult. The acceptance of like as much as we want to tell you he will get better we cant always necessarily say that, we have to go off the data that supports our clinical decisions.

I: What are some of those tools you use?

R: The therapy team uses the Come recovery scale JFK, each discipline uses specific discipline specific things. Obviously, they may not meet the specific things, we track it. It seems like your tracking a lot of non-verbal things. You are with these patients so closely. The ones that neuro storm, I have a monitor on them and their pulse elevates maybe they are wet, or need to cough or something is bothering them. He can't tell me but his body is responding with his pulse or blood pressure elevating, just discomfort subtle things like move my hand or leg. The patient doesn't necessarily need a pulse ox on but it helps, like caring for a newborn but a lot bigger.

I: Anything else I didn't ask you or that you want to share about anyone we already talked about?

R: I don't think so.

I: We are all set thank you so much.

Clinician Interview 11

I: Interviewer

R: Respondent

I: …..You're okay with going forward?

R: Yes.

I: So the first thing I want you do is I want you to think about a patient that you've worked with in the past that was in a disordered state of consciousness so vegetative or minimally conscious that you were surprised. Something about them surprised you when you first met them. Your first encounter with that patient was surprising to you.

R: I can't think of any honestly. I just think of all the patients that I've worked with that I guess I know other than just the surprise at when I first started working in the field and just realizing you know that someone can be so impaired and yet they can still come back from that injury. So, I think the surprise factor was just in my early education of learning. That this is just kind of what the body does when it shuts down and goes inside itself and it starts to heal and then you can come back from that.

I: So, can you think of an example, can you think of a specific case where maybe not your first encounter, but you were expecting a patient to do one thing maybe based on team conference information or whatever and then when you actually worked with a patient they did something completely different or that you weren't expecting to see?

R: Yes. I think I've seen people, so like, I get to do things like animal assisted therapy. So, I bring dogs into the hospital and I've seen people perform better when they have a motivating factor that might not be present or available in other disciplines and so if they have something like a dog that motivates them they're performance is better. I've seen people just being able to track looking from side to side,

reaching out sometimes to reach to touch, moving fingers when we didn't really expect them to or even just smiling when they see the dog walk in the room and we haven't seen any kind of an emotional expression, so with things like dogs and music.

I: So, can you think of one, can you think of one particular example of a patient that you brought a dog into the room and can you describe that interaction with the patient and the dog and what you saw?

R: So, the patient was sitting facing the door and when we walked into the room with the dog, the patient's head was down at first and he wasn't making any eye contact or making an effort to raise his head and when the dog came in we had to cue him to look and then he raised his head and his eyes widened and he started to smile and then when the dog came closer to him he leaned in towards the dog more and when we put his hand on the dog's head we saw him moving his fingers as if he was trying to scratch. He wasn't able at that point to reach purposefully to do it, but when we put his hand in place, he moved his fingers. His sustained attention was longer in a session where if I was trying to do any other type of activity, I wouldn't necessarily get much participation, but when the dog was there I could get him to really focus for ten to fifteen minutes.

I: Okay, interesting. Good. Can you think of an example of how like what you saw with a patient when you used music, since you mentioned music as well?

R: Yeah, so, it was a patient that had not verbalized, but was he was demonstrating that he was alert and he was using hand gestures to say yes and no and to indicate what his preferences were, but he hadn't been able to verbalize. I was working with an intern and he was a younger guy and he was we were just kind of joking around with the patient and my intern was talking about being in a talent show and how he had lip synched this song, "Ice, Ice, Baby" by Vanilla Ice and so my intern would start by he would so the words…this is embarrassing (laughter)…so the words the song goes, "Stop, collaborate, and listen" right? And so my intern went stop, collaborate, and he stopped and the patient mouthed the

word “listen” we didn’t hear anything at that time, but as we continued on with the song, he would finish the sentence and gradually we started to actually hear him verbalize the right word. So, we had tried everything, including songs that he, his wife said he liked this type of music. He didn’t respond to those, but it was a song that he would have known as a young teenage like 12 or 13 years old. And so somehow it stirred something different and because he was kind of, very close in age to my intern, my intern was a male and they were similar ages and backgrounds probably and so just this song and because it’s such an iconic song and everybody knows it and he responded. And I don’t know if I would have gotten the same response from him if it would have been me, but because it was another “dude” you know and then just to be able to finish the sentence versus trying to play it and have him sing along or something. I don’t think that would have accomplished the same thing either.

I: Right, but you said then he eventually progressed to singing more of it?

R: Yes, and that was the very first and it just so happened that the doctor was nearby so we grabbed her and we pulled her in and my poor intern was really embarrassed because then he had to sing in front of the doctor, but yeah it was just stunning to see the change just because of that song and then he did eventually go on to, I mean he is completely verbal now.

I: Oh, that’s exciting. That’s awesome. Can you tell me, can you think about a patient in this population that you’ve worked with that you felt stuck with? Like, you know, for whatever reason you just felt like you were stuck.

R: Yeah, I guess the people that don’t really emerge. The ones that I don’t see any response from. I mean most of the people that I’ve worked with, I can get something. Whether, it’s just eye contact or some type of movement that indicates that they are aware, but I have worked with I would say two people where I just didn’t get anything where I felt like they weren’t really even aware that I was there

and I would try movement, music, touch, any kind of stimuli to get some kind of response and I just didn’t get anything. So, yeah, I just didn’t really have any tools to go any further with them.

I: So, when you think of someone emerging, when you said that those people that don’t emerge or they don’t respond, when you think about emergence, in your mind what does that mean? Describe to me a person who emerges.

R: Well, I feel like it’s usually a pretty long and slow process and they go from kind of a vacant stare with no recognition and no following or tracking movements and usually the first thing we see is there is some sort of eye contact, some sort of effort to follow an object or just pulling away if you touch them and they pull away or if you put your hand in their hand and they respond in some way with a hand movement. Usually, those are king of the first signs that we start, that I start to notice. I’ve seen people sometimes that won’t respond to me, but when a family member comes, then it’s a whole different story. I feel like the family being there is a critical element to their recovery. Not only that, but I feel like the families, I’m probably getting off track, but the families I rely on the families to tell me what the patient likes and what they don’t like and that helps me try to motivate the patient and get their attention more and to put pictures up in the patient’s room and have people’s names and stuff like that so we can use those pictures to get the patient to track, follow, or to smile. So, I can put music on that they like and that will soothe them maybe so they start responding to small things and sometimes it takes a really long time. I’ve got a patient right now that just, it just happened really fast. When I first saw him, I thought it was going to be a really long lingering state of minimal consciousness and it just really quickly progressed and he’s walking and talking and it’s been a month.

I: So, I’m going to stop you for a second because I want to capitalize on something quick. So, you said that when you first saw him, you expected it to be a long lingering process. So, what about that first impression made you think that?

R: I think that because he didn’t really show me any sign that he was responding to my questions or my presence in the room. I think it’s the eyes. I look at the eyes and if the eyes are showing signs of recognition, if the eyes are moving in a way that looks purposeful versus just kind of you know random movement rolling around and not really seeming to have…there’s just something about the eyes whether there’s a presence or not and I feel like that presence.

I: I know that’s not very tangible. I know it’s kind of a clinical gut instinct to, but can you try to describe that to me like what about their eyes makes you think they’re present or…

R: When I think of the people that I didn’t feel that they were present at the time, I felt like they’re eyes were, they were open very far, so they just had their eyelids kind of half-mast and there’s and it was just sort of an empty stare I guess. I didn’t feel like it was looking, it just felt like, it wasn’t moving, their eyes they’re not moving really and it just looks through and not at and a hollowness kind of. There’s like a hollowness that the eyes aren’t, there is nothing resonating I guess.

I: That’s a good description. I like that description. Okay, I’m going to take you back again because you said a lot of things that have really triggered some questions for me. So, the person you were talking about that you said when you first saw them, you thought it was going to be a long process…can you describe maybe one activity that you did with them that first time and how they responded to it?

R: Yes, so, I’m a very non-traditional sort of out of the box therapist and sometimes what these young males respond to is not necessarily a clinically standard and appropriate type of approach, but there’s a tv show called, “Jackass” where these guys do ridiculous things and oftentimes they’re just gross and inappropriate and in every way unacceptable behavior, but I get a better response from “Jackass” than I do almost anything and so I put this young in a room, so, the first thing that I saw in his response was there was no one in the room and I had it on the computer and I had to position him so I thought that his good side was facing the screen. The first thing was that I noticed that he was watching the screen

and not just sitting there you know just unaware. He was focusing on the screen and he smiled at an appropriate time so he recognized that this was the moment that was funny and he smiled at the right time and so that was my first I guess sign that he was starting to emerge. The other thing that I feel important is being in social group interaction and I think that oftentimes you know these folks are seen bedside by one clinician maybe two and there is no laughter, there's no energy, and they do not have that human connection and so I make a point of bringing those people into a group setting even if the people are playing a game and they can't participate in that game, they hear the voices and they hear the laughter and there's an energy that moves in the room, that I feel like I see people respond sometimes to those group situations when I don't see that happening in a one-to-one bedside in their room. So even though they aren't able to participate, just the experience of being surrounded by people and laughter and just sounds of every day interaction and it's not in the cafeteria or in a, it's a social energy that they respond to.

I: Those are all good examples. The other thing I want you to tell me about is you mentioned that sometimes you can't get a response out of someone, but when you bring a familiar person or family member and they'll get a better response. Can you think of a specific example with a patient where you were having a hard time getting a response, but you saw a better response with the family?

R: Well, I guess I don't know if it's a "better" response…

I: Or a "different" response.

R: They start to cry when their family member arrives. If they've been alone for a long time and they are not really showing much awareness or interaction with me or other clinical or other individuals that are working with them, the family member comes in the room for the first time and the patient starts to cry. So, it's not, you don't want to make them cry, but at least there's that recognition. It's like finally the

person is here and it’s such a relief to them and even though they are not responding in other ways, there’s a recognition and a release of emotion.

I: Now, I want you to think about one of your most memorable experiences treating a patient that started out in a disorded state of consciousness and tell me about that.

R: I worked with a young man who, his mother was from Guatemala and so he and his wife were both Latino. Mom was born and Guatemala and lived much of her life in Guatemala before she came to the united states and through conversation with and this is a man who the descriptive information that I got from rounds and from the paperwork really was almost like worst case scenario. It felt like we’re going through the motions because we owe it to him and we have to, but our expectations were very minimal for his outcome. So, I’ve never gotten the sense from the team that I work with that, it’s almost futile, but we’re gonna do our best, but that was sense I had with this man and so his mom and his wife were bedside constantly and I’d developed a good rapport with them and we started talking about traditional healing practices because mom had relatives in Guatemala that were, they were Shamans. Mom early on had been exposed to this indigenous practices of healing and so had relatives that were actively utilizing that in her culture and the patient had also been exposed to that spiritual practice of healing, but also had a Christian belief system as well and the wife was sort of like, she wasn’t really sure what she thought about it, but she was open to it and very willing to explore different options and this man had been with us for months probably. When the conversation occurred with mom talking about I wish I could bring my cousin from Guatemala to come and do a healing ceremony and so it just so happens that I know people in the twin cities that are indigenous healing spiritual healers and so I asked mom if she wanted me to contact them and get information and give her phone numbers or contact whatever. So, I made sure that the people were still actively in the field still, seeing people. Gave mom and wife the contact information and let them kind of initiate whether or not they wanted to see or have the person come here or what they wanted to do with it. So, it ended up that there was sort of a group that came.

It was actually a woman who didn’t even speak English. She was here in town for this trauma institute where she was doing a lecture on healing trauma in indigenous cultural ways and so she was here and two apprentices. So, they came in and probably broke a whole bunch of rules in our protocol with what they should or shouldn’t do. They had different plants and different, they put out a table cloth and they had all these little statues and little shells with pebbles and a holy water bottle. There was a candle that was lit, that wasn’t, you know, don’t record that (laughter).

I: I won’t tell anyone (laughter).

R: So, I participated just in the sense that I was present to make sure that no protocols were really broken or the patient didn’t have any…I wanted to make sure there was no safety things and so I was present during the time. The first time they came, it was just the patient and his wife and it went probably about an hour and a half. There was singing, there was praying, there was shaking of rattles and drums and things like that. There was two people working with the patient and then two people that worked with his wife. They did breathing work with the wife to release emotional stuff and they did some massage. There was prayers in the Christian tradition and prayers in the Mayan tradition. Overall, it was a very emotional and amazing experience and the patient had been here for months and had no real response that we could see he was changing. So, immediately after that experience he kind of went into this even deeper sleep, it was like he was knocked out for three days and on the third day when he woke up, he was present. His eyes had changed. He was tracking and showing responsiveness and he just went on this remarkable recovery process that nobody here can explain it. People talk about it and nobody has an explanation. People say it was, he was a miracle. I don’t know how much everybody on our team knows about this thing that occurred. I made a comment, I documented it in the chart, and I talked to a few people and said we had traditional healers that came in, but I didn’t really share the extent of what I felt in that room…what was happening. I don’t know that people made a connection that there was a very strong spiritual experience for this patient and there’s no doubt in my

mind that something, a miracle happened for him because as clinicians I just don't think we had the tools to bring him back from whatever place he was in. He had another visit, maybe two weeks later, where his mom was there and not his wife. So, his mom was able to experience and they were surprised when they came back and saw that he was actually aware and alert at that point, but his wife has, she's from Chicago, and she has come back to the twin cities to stay in touch with this indigenous, the local woman who does the practice, just for whatever spiritual healing she needs. I got a phone call from this guy last week…

I: The patient?

R: The patient called me last week to tell me he wants to be a motivational speaker and he is working, he's independent in every way except I don't know that he is driving yet, but it's less than a year since his injury.

I: His injury? That he called you and?

R: Well within six months he was home. I think his injury was Memorial Day weekend. He was home before Christmas and came back here with the intention of participating in our extended rehab (8:21??) unit. When he got back here, he was already beyond what they were really able to do with him. He was not challenged by the program. He said, "I can do better on my own at home." He stayed less than 30 days went home, got a job, was actively participating in every, he was coaching the kids' football team. They went this spring to Guatemala, the family went to Guatemala and traveled together. He climbed a pyramid. He was running backwards and forwards up the mountain, according to his wife.

I: That's amazing.

R: Yeah, considering her was our worst-case scenario patient where we maybe expect that they might regain some level of function and he's functioning on a level that no one can explain. He called me last

week and said, "{NAME}, how do I be a motivational speaker? I want to help other people. I want to talk to other veterans and people that have been injured the same way as I have." I can't explain it. So, that was probably my, I've had a lot of amazing patients that come back and share their stories, but that one was…

I: That's neat. That's the rewarding part of your job to, well it's all rewarding, but when you hear that stuff. I know you have to go, so I want to ask you just one more question. Now, can you think of a frustrating experience that you've had with this patient population? So, someone…

R: So, I had a patient that I had a really great rapport with and he was doing really really well and just really had a great attitude and just was super motivated, playing ping pong…he was doing great. We had an outing planned to go out to a museum. Did all our planning and set our goals and talked about what his expectations for this outing and as we were leaving, as we were walking out to the parking lot. I became a little bit concerned about his movement. It was a little clumsy and a little slow. I was observing as, it mostly happened when we were heading out to the parking lot, when we were in the museum we were moving slowly enough I guess that he was moving just fine. Anyway, on the way back walking back from the parking lot, I felt like I needed to help him to balan-, I had to jump in a couple of times because I thought he's going to stumble. And he yelled at me and said, "I'm fine, I can walk, you don't need to do that." We got back and I said the nurses there's something wrong. They rushed into his room and all these tests and everything determined that…and they called…I don't know rapid response maybe and he had a bleed because I was the person who told the nurses that this behavior was occurring and that's what he associates me with. That he believed that I was the reason that he ended up staying…that he had a relapse. It was like somehow he felt like it was my fault. He didn't understand why, but he knew that {NAME} is the one who all of sudden, now all of these doctors are looking at me and if she would have just kept her mouth shut, it would've been fine.

I: Oh no.

R: And he didn't realize that if he would've kept bleeding… So, from that point he wanted nothing to do with me. I actually had to ask one of colleagues to trade off with me because he was fine with everybody else, but I was the person that caused that whole setback and yeah, you know, I don't hold it against him, but he wouldn't have anything to do with me because I was the bad guy. Yeah, that was probably my most challenging setback.

I: Have you ever had a frustrating experience with a, working with a patient that was still in a vegetative or minimally conscious state?

R: Frustrating in the sense I guess, I don't know what to do with them. I get frustrated not with them, with my lack of tools, I guess. There have been two people since I've worked here, where we've actually had to use a posey bed, like a vail I think it's called, a vail bed because they're just too impulsive that you know, even when we have a sitter, someone one-to-one sitting in the room, the person will try to get out of bed and the nurse can't get there in time and so we've had to use, on two occasions, these vail beds. Those are the people, they're just so restless and they want to move, but they can't and they don't want you to hold on to them because you are making them lose their balance or whatever and just because I sense their frustration I guess. That's really hard for me to have to have somebody you, you have to confine them for their own safety and limit their movement, either holding on to them all the time or strapping them into this chair where they can't get up. It's a safety thing and they don't understand it. Yeah, you can just see the anguish, they get angry, so that's really hard for me I think.

I: You've shared some really awesome stories with me. You gave me some great descriptions too and I appreciate that. Was there anything else that you can think of working with vegetative and minimally conscious patients that I didn't ask you about, that you think that I should know or that you want to share with me?

R: I feel like the family is really key and just a personal approach you know, if we don’t know what motivates them, we might miss something because they’re just not interested and if you can get their attention, if you can find out what they like an somehow use what they like to get to them, I feel like that makes a huge difference, but those are things that I said already I guess I can’t think of anything else.

I: Well then that’s it. I really appreciate that you did this. Thank you.

Interview 12 Transcription

I= Interviewer

R= Respondent

I: ….I do need to tell you that this is completely voluntary, so if you decide that you do not want to continue with the interview you have the right to do that and you participating in the interview is your consent. So yeah, you can answer any questions. You can tell me that you do not want to. Do you have any questions right now about how this is going to go? So you're okay to continue?

R: I am.

I: So the first that I want you to do is I want you to think about a patient that you've worked with that's been in a vegetative or minimally conscious state when they came here and think about a patient that you were surprised when you had your first encounter with them.

R: With the patient?

I: With the patient.

R: Not the family?

I: Not the family. Can you think of..?

R: Like even just first meeting them?

I: The first meeting of them, you expected one thing and maybe you were surprised by what you saw.

R: I think just, so coming into I been here at the xx for seven years and I hadn't worked with patients in a rehab compacity still in a coma until then. So, um I think so since then, oftentimes patients in a

vegetative state have a similar presentation, so they're not responding they're not necessarily not making any eye contact when you enter the room. So I would say probably just meeting with the first patient in a vegetative state would be when I was surprised. And it's mostly just taking that all in. I think young people in particular just to see them, just in bed, not moving and not engaged with their environment. The first time you see that can be surprising, but I think, I feel very comfortable moving forward on being in the presence of patients that are in a coma. I always talk with them as if they are hearing me because I don't feel like we ever really know for sure. So introducing myself just as I would if they were awake and looking at me when I enter the room and explaining why I'm there and what I'm doing.

I: Can you think of, so maybe thinking back on the first patient that you saw like that. Can you give some examples and you did give a couple examples, but can you give some specific examples of things that person was or wasn't doing that was surprising to you?

R: Yeah, I think sometimes just seeing the equipment that's attached can surprising. You know you've got the I mean if they've got fractures, they've got splints, we get people with bad motorcycle accidents. They've got kind of equipment all over the place. They have a neck brace on. They have the trach with the dome. You know they're just kind of, it's a lot hooked up to them. And then them just being in bed not responding any of those things that are going on in their environment. And then if there's things as a social worker, I'm not the medical provider, so if there's some IV pole going off or something like that, you know, that not something I'm comfortable managing or dealing with and I go get someone else.

I: Has there ever been a patient that maybe in a team conference or something like that was just described in a certain way and then when you went to interact with the patient and the family you were surprised by what, maybe it was different than what you were expecting based on the description that you had been given.

R: I think as far as when the patients are emerging and sometimes we see patients where that's moving pretty quickly. So maybe the first day they got here, they had flown in. They were sedated. They were really kind of conked out. Then a week later, I might go in there and notice that when I walk into the room they actually appear to move their head in my direction as they heard me enter or responded to my voice. So I think it's more been that they're I've been surprised that they are more responsive or alert versus if I know that they are in a coma, then I may expect them to just be lying there and not interacting.

I: Can you think of someone who maybe during their whole course of their stay here was surprising to you?

R: Yeah. I think some of our patients that like have emerged and as the social worker, I'm not seeing them every day like therapy and the nursing team is. And so coming in maybe weekly to see them there's always new things happening and that could be exciting and certainly kind of rewarding and encouraging. Just as it is to their family. I think it is to our staff too. Someone who isn't seeing those daily changes, it's always exciting to come in and see them more responsive or up in their chair for the first time, is always a really exciting thing.

I: Can you think of specific patient and kind of describe that?

R: Yeah I have one patient that we worked with really closely for many, many months and who was very slow to emerge from their coma, but and had bumps along the way that set them back as well, but those kinds of examples happened a number of times throughout his stay. I think I remember the first time he was up in his chair. He had been fit for specialty chair and just how much more comfortable and more like himself when he's up and out of bed and positioned in way…it appeared that he was taking his medication a little more frequently. And then there were other times where he was maybe out in the hallway with in a wheelchair maybe on his way to an appointment and I could hear like calling out or

verbalizations. So from a patient that we hadn't heard anything before, now was making attempts to make communication. And like seeing him smile at me and interact for the first time was also kind of memorable in terms of again that becoming more aware of his environment and alert and he was a pretty charming young man and I think pre his injury. So and had a big beautiful smile that looked a lot more like the pictures that you see on the wall than the patient we'd seen for two or three months in the bed.

I: So you saw that change? You saw something change in his? Can you describe that?

R: I mean he was like I said even as he wasn't able to verbally communicate, he would acknowledge the presence of other people in the environment and I think he had just a really big smile big teeth and would light up when people came into the room or said his name or kind of encouraging him which in my role as the social worker a lot of little stuff I'm working with the family on, but when I'm visiting the patient it's usually just kind of to be supportive and encourage them and point out like "wow, last week when I saw you, you didn't, you were still in your bed and now you're up in your chair." Just kind of dialoguing to them what I'm noticing as changes. And if I heard it either in the team meeting or from the family member that "oh they started giving a thumbs up." You know, then I'll try to ask them to do some of those behaviors that I've heard they're able to do. "Can you give me a thumbs up and let me know that you see that I'm here?" That kind of thing.

I: Okay, I'm going to take you back for a second there's two things that you said that I want you kind of expand on. So you were talking about the patient who had been in the bed and got up in the chair and was taking in his environment more. Can you describe that to me? Like give me some examples of how you knew he was taking in his environment more.

R: Yeah, my perception that they're taking in their environment more is attempts at visual tracking. So like noticing that there's either a movement in the room or a change in light if we open the curtains or

those kinds of things. And then I think even head turns to sounds and different voice. Recognizing that they are hearing me speaking to them or making attempts to acknowledge that I'm there by turning their head or their vision. I think physical responses. This young man eventually got to the point where he would do a fist bump when someone came in the room as a greeting. So I think those, that's my sense of when they're more, that they're interacting with their environment.

I: Yup, those are good examples and then the other thing I'd like you to just talk about a little bit is the bumps along the way. Can you describe what some of those bumps were?

R: Yeah. So this particular patient had I think complications both with infection and respiratory distress. So he had been on a pretty nice steady course and was sort of on a track of slowly interacting and emerging and interacting with the environment and then he had a pretty major respiratory episode and then ended up back in the intensive care unit. And actually back on the ventilator. So he had had his trach removed by that point so they no longer had that access and that had been fairly prolonged process of in discussions with his mom and the team to get to the point of removing that trach and then he kind of went back on it after, so having to be reventilated. And then he had developed like ARDS or the really compromised respiratory function in the ICU so I participated in the family meeting with the ICU staff and our rehab doctor and his mom about really deciding what she wanted to do with the severity of his brain injury, now compromised with the possibility that he might not be able to weaned from the vent a second time. And so, that's why that case stands out remarkably like miraclely, he did he stabilized over the next few days and ended up being able to be weaned from the vent. He did have to be retrached. After a period of additional time in the ICU, he did make it back to the rehab unit. His level of alertness and interaction with the environment had kind of slipped back to many weeks prior. So it kind of felt like it was restarting again, but we slowly saw him return to making eye contact and (26:05?) and he using some thumbs up, thumbs down kind of (inaudible).

I: So did he eventually progress to the point that he was before that infection?

R: He did. It took a long time, I would say on the rehab unit, we unfortunately, while he was still on acute rehab. He didn't, he probably just made it back to the best he did before that respiratory episode. So and then we had to make the, you know the kind of, continued throughout that process of having a difficult conversations with his mother about where he would need his care because he was so weary. He was completely dependent in all areas of his care. So working with her on…what she was adamant about and what ended up being a temporary long-term care facility stay an eventual building of an accessible home and the support to bring him home. And then as returned as an outpatient, over months we did, I observed him and his mom would make a point of stopping by on our unit or even my office to kind of show us the new things he was able to do and so watched him to continue to actually be able to form words and he advanced to be able to eat, uh, diet and feed himself with just some assist. I think he got to the point where he could move his feet a little bit and move himself in his chair some. Overtime he did get past that level that we saw prior to the respiratory event.

I: Well I want you to think about a different patient, maybe or a time when you were working with a patient that started out vegetative or minimally conscious and you felt stuck with for whatever reason.

R: We had another young woman probably similar age to that young man who her's was more of an anoxic injury versus his was a traumatic from a motorcycle, motor vehicle accident. Her's was from an anoxic event. I can't remember what the cause was, but she found down in her home not responsive and they determined that she had an anoxic injury that was causing her to be in her vegetative state. She came to us from Arizona where she had been living to Minnesota where she was closer to her mother and sister who lived in Iowa and so they would visit often. But her situation, she did progress to being able to make some eye contact and seemed to respond to the sound more often of those familiar to her so like her mom, her sister when they visited would make more of an effort to look towards them,

but didn’t make much progress beyond that. She was remained on a peg tube and did end up breathing on her own so we removed the trach, but feeling stuck there because her family didn’t have the resources to be able to bring her home. They wanted very badly to bring her home and her mom was a retired nurse and so she had sort of the skillset to be able to do that, but she just physically couldn’t provide the around the clock care and didn’t live in an accessible environment it wasn’t a place where she could get out of her current home and her sister was very committed to wanting to bring her home as well, but had two children of her own and a job that she needed to continue to work. And so just navigating that with them and feeling stuck that there wasn’t a different option. The other thing in terms of funding and availability, she didn’t have the right funding to be able to go to a lesser level of care like a group home or something like that either. With Iowa Medicaid, you need to be in the state to get approved and even that there’s some stuff that have to be done to be able to get the right funding for more community based setting. So we did have to work towards a nursing home discharge for her and get the facility and the local VA to agree that they would take her without a funding sources knowing that her Medicaid would be approved at some point.

I: Can you think about your memorable experience working with a patient that’s at least started out in a vegetative or minimally conscious state when they got here?

R: I think the first patient that I talked about is probably the most memorable for me. In terms of details, I have another patient who is more recent in my work history. A young man, he was active duty, he was he came to us from New Mexico, he came from far away, I guess that’s what I communicating there. Again because his family lived in the Dakotas, one of our bordering states and he too came to us in a coma and emerged more quickly in terms of waking up and following commands and interacting with the environment, but he too had some setbacks. He developed an infection in his brain and had a couple weeks in the ICU, so he too was kind of on the track. The team, the family, everyone was kind of feeling excited and energized. We were seeing him waking up, changes, this is gonna be permanent and then he

developed this infection so all of sudden you're seeing rapid neuro changes and he was placed in the ICU here at the medical center. I think during that course he was treated with a long course of antibiotics and had to have a drain in place, so he wasn't able to come back to the rehab unit. And so that was kind of the delay in his care. I maintained communication with the ICU team in a minimal amount, but continued to check in with his dad who was here bedside, kind of see what kind of questions he had and reassured him that once he gets his course of stuff was happening that we would, our efforts would be to bring him back to the rehab unit and see where we left off. And he came back and bounced back very quickly after that. That was long stint though and it was contingent on his antibiotics and things like that, him coming back. And when he got back, he did continue to make pretty rapid gains on awareness and being awake, tolerating therapy with his pain meds.

I: Can you think of something like again, an example of something you saw him do that was memorable to you?

R: He'll always stand out to me with his savvy use, very early on in his recovery, with his iphone or his smartphone. So even though he was verbally making his needs known very well, and communicate a full sort of statement when he got a hold of his phone, his dad was reporting to me these conversations back and forth that he is having or text messages. His skills with his use of his one hand because he had the one hand was hemiparetic So he could get, keep the phone in his hand and he could very easily send out messages and that was a whole different sort of piece that required discussion with his family and our team around his use of the social media and those sorts of things because he was putting information out there due to his lack of insight that could make him vulnerable such things that weren't necessarily accurate information and then his dad was in charge of sort of having to monitor…

I: Kind of being the filter for that?

R: That was really a memorable case in terms of these very young people who that is just such a natural, second nature for them.

I: Different right? At least for me, I don’t know that I would do that. Okay, so this might feel similar, but can you think of a rewarding experience working with someone in this state?

R: Yeah, I think all of those three patients really, I mean working with this population from the social work lenses is very emotionally taxing like with the team and with family, but tends to be very rewarding as well because I feel like these are patients that their families were really kind of had been given the news that they might never wake up and so any changes or improvements that happen. I think their support system is so just elated about and I think is inspiring to the team and all three of them did make progress. One at the end there, the most progress of the three in terms of just continuing to meet all of his goals and progress through more independence, so I think back to your original question was…rewarding?

I: Um, rewarding.

R: Yeah, so I think just being a part of that and be able to help. For me, my role tends to be a lot with those patient with the family, but as the patient is becoming more involved to rewarding in terms of helping them to participate and the next steps in the planning for their care after the rehab they have here, I think is very rewarding. And even those patients that don’t emerge so much, the gratitude that I experience in my role from their family about helping them through this very difficult time and what I’ve heard multiple times before is that I didn’t know what to ask, I didn’t know what I even did and you were there to tell me you know recent experience probably should apply for disability or we should look into these services closer to home or we should start talking now about what are the plan A,B, and C that you would like to see happen depending on the course of your loved one’s care.

I: So, talking with families…can you think of about maybe a situation where you were feeling stuck because the person maybe wasn't progressing and how you communicated that kind of emotion or news with the family?

R: Umm yeah, I mean we knew that early on in particular that tends a really piece so what I've found really useful is to really make early on good connections with the family and build rapport and check in. So they are comfortable with me and my intentions when I am coming back to them with information from the team about whether it's lack of progress or sort of setbacks and what that might mean in terms of their length of stay in our program or what we need to plan for in the future.

I: Can you think of a specific family that you maybe had to talk them about maybe lack of progress?

R: Those are the, back to that young woman, I had a lot of conversations with her family about how, although we've seen small improvements in her eye contact and those sorts of things that there wasn't strong evidence from what we could see so far and at that stage in her recovery that she was going fully emerge from her coma and be able to get back home with them in a capacity where she could care for any of her basic needs on her own. The anticipation was that, we can't predict the future and everyone's injury is different, but based on what we had seen so far we expected that she was going to require a lot of care and assistance and be restricted to wheelchair and continue to have a catheter or require packing and tube feedings and those sorts of things.

I: And how would you describe her level of functioning at that time? Like what was she able to do? Was she responding to things in her environment at all? Or?

R: I feel like sort of the max area for her was just some simple command following like maybe open and close your hand, or lower your chin or something like that and so eye contact which she never got to a point where she was verbally communicating anything and yet I think her family felt like she was understanding more than she could communicate. And I think that's a hard area to, as a team, to

respond to because we don't know either. It's obvious what's being communicated out and lack of ability to do that, but trusting and knowing whether she was receiving information and like there some times where her family was communicating something very emotional and sentimental and she would have a tear drip down her face which led them to believe and I would say myself as well to some sense that she was understanding more than we would typically think or at the moment even and that's the part to I think to communicate to families is that that with brain recovery like that it could be so variable based on their level of fatigue and alertness and that sort of thing.

I: Yeah, those are challenging conversations to have. Can you think of a frustrating experience that you've had?

R: These patients because of their level of need or services after they leave here tend to have lots of frustrating roadblocks and the third patient that I had talked about who had emerged nicely, there was frustration there because we were working with the um the Air Force, that he was still active duty at that time and needing to find…we didn't know for sure how long his Tricare coverage under his Air Force service was going to last. We didn't know based on the circumstances of his injuries if he would be eligible for VA care after if he would potentially going to be chaptered out of the Air Force which would mean that he would then not be eligible for VA coverage and then it would have been (inaudible 8:35) I think that we just sort of determined that he would ended up needing to be on Medicaid in the state that he was living in. So there were just a lot of frustrating dynamics there in terms of not being able to get straight answers to create a easy plan for the family and then we hit roadblocks as well in terms of when I did find a facility that could take both Tricare and VA and he could potentially could stay there all that fell through, you know, really trying to make sure that there wasn't going to be a sudden change that needed to be happened. So there were a lot of frustrating pieces in that one. In the end, it ended up being very rewarding. He got to a facility with just an enormous amount of time required, in terms of research and advocating and calls and phone conferences and all that stuff, but we got him to a neuro

rehab center where he could continue in more of a community based setting which is really what he needed. He really maxed out at what he could progress in a structured hospital setting and he needed sort of that more home-like environment to really push himself forward. So we did get him to a neuro rehab facility in another state and found out that he later continued to progress nicely and has even discharged to a lesser level of care.

I: So can you think of uh, has there ever been a situation where it was frustrating because maybe what the team was seeing as far as behavioral responses go and what the family was seeing was different?

R: Yeah.

I: Can you describe that?

R: Yeah, I think there's unique frustrations through my social work lenses. I recognized where the family is coming from and why it would be hard to kind of receive that information or trust it or accept it and be ready to move to the next steps based on what that information is. So that, so I think there certainly is frustration when you feel like you can't explain the situation in any other way by any other team member to help them just move to that stage of their grief and acceptance which would allow them to participate in discussions around next level of care and not only what they want, but what would their loved one have wanted in this circumstance in this situation.

I: And can you think of any, can you think of any specific again any specific behaviors or anything that the team was interpreting one way and the family was interpreting another way?

R: Yeah, I mean I think we certainly have patients where the family member feels that they that there's more going on in terms of the brain function of the individual than the team does and that (?5:24) at movement that the team may think is just automatic and not volitional that the family is really hanging their heart on as being signs that they are responding and that they are coming along more quickly than

the team observes. And I think our team does a nice job of explaining what we think that might be, you know, the physiology of, that the patient, people are responding physically, but it may not be exactly to the stimulus that's taking place.

I: Yeah, that's challenging. Are there any other questions or is there anything else that you want to tell me about working with this population that I haven't asked you?

R: I think from the perspective of the social worker and the case manager on the team and I don't know that I think this is a reflective, I think this is somewhat unique to the unit and the population we work with here partly because like for me my caseload is much smaller than if I was in an acute rehab center in another hospital which is what I did before this. I feel like we have a really significant role in that piece about building that rapport with the family and helping better understand what's going on and what to prepare for and being able to see when there is that discrepancy between what the team is seeing and where the family is at in terms of their understanding and awareness and engaging in (?3:35) team that we are having more conversations that I'd like to have the family observe you in session would you explain what you're doing and why and what that means because as I'm talking with them and explaining I don't see it and I don't see that they're making the connection. So thinking as a team about what are the ways that we could engage the family to be able to do that. And then I think just really helping the team to as best as I can help them understand where that family member's at in their stage of acceptance and grief and loss around this devastating injury and helping the team maybe understand when we're seeing some fairly abrasive or angry behaviors from a family member or unrealistic expectations. I've felt like I was turning blue in the face communicating to the team that I really did strongly feel that his mom had a reasonable expectation and an understanding of the severity of his deficits, but despite that her sole mission was going to be to advocate and for whatever help and service and funding source and resources her son could get. That was like her new job and which you saw was her focus and it wasn't because she didn't have really a good in terms of her interest in bringing

him home. It wasn’t that she didn’t think that there were going to be hiccups or that there would be an emergency and she would need to respond to it. She was well aware she didn’t wide support network beyond herself and trying to communicate that to the team that despite all of those things she still wanted to move forward with getting her son home and that she was prepared to deal with what came along.

I: Yeah, I feel like these families learn to become advocates very quickly.

R: Because a lot of families have said, if my loved one was written off from the minute the neurosurgeon saw him. I can’t tell you how many times I’ve had patients say the neurosurgeon told me my son would never wake up or do anything; would never even look me in the eye. And I’ve had other family members who even if their loved one has improved who have those conversations about, I don’t know if I did the right thing by keeping them alive I don’t know. My son would have never wanted to live like this. I’m grateful that he’s still here, but as the person who had to make that decision in that moment; I don’t know that he would want this. He looks like he’s suffering or looks like he’s in pain. And so having those kinds of conversations it’s always really hard.

I: Yeah, it is hard. So much we don’t know, you know, still that we don’t know.

R: Yeah, a very fascinating population like I said very, I’m grateful that we have the capacity to have two patients emerging from coma in our unit at a given time.

I: Yeah, it’s intense. Yeah it’s an intense population.

Clinical Interview 13

I: Interviewer

R: Responder

I: …do we have your consent to continue?

R: Yes.

I: As I mentioned, we're talking about those patients who are in the disordered state of consciousness and I want you to think about what was your most rewarding experience treating one of these patients?

R: Well for me because I don't frequently do a lot of work with them while they're in that state, it's sort of more with the families. So, it's as they emerge, then I'm more engaged with them so. I think it's just being able to actually work with them versus just being able to provide family support.

I: So, can you I guess, this is a unique situation because we've mostly been working with clinicians who have been, you know, start to finish, so maybe if you could take me through a technical case if you start with the family right away, if you come in midway kind of give me a little bit of background and understanding of your role with these patients.

R: So, frequently the moment consult comes in from psychology we'll go in and want to sort of just a case, like a chart review. We're always involved in rounds every week and supporting the team, but frequently we also get consult at the same time for family. So, from the very

beginning we're working with family when patients are in state where they're actually able to engage in psychology services. So, it's different, so I'm not actually doing some of the like assessments that they're doing in their speech, OT like the coaching and things like that. And so, we oftentimes we have family members that are close by that either observing or engaged very closely with the staff, observing kind of all of their therapies or most of them and so frequently are engaged with us, kind of reinforcing education that they receive from the therapist, trying to help them understand the difference sometimes between what may be reflexive response versus something that is more indicative of engagement and sort of them actually being alert and participating in a meaningful way. So, that's where I think oftentimes the struggle is for family and also just offering support because a lot of family members get that and understand and at the same time are still trying to remain hopeful while deal with grieving at the same time {Inaudible 3:46} and also provide support around things like being away from family for prolonged periods of time and then being away from home and sometimes putting their careers on hold and those kind of things.

I: So, the one thing I want to zone in on first that you talked about how big families distinguish between reflexive responses versus the engagement. Can you give me some specific examples of different things that you've encountered with these families since, that they have seen, that they have talked to you about?

R: Sure, when patients start breathing on their own and when patients start opening their eyes that's really exciting for family members, but it also and may be indicative of progress because we them very early on so the other piece is that sometimes really trying to help them understand what it looks like as they move from and emerge I guess. They're actually tracking

things where they're actually having some sort of meaningful interaction, consistently be able to reproduce different kinds of responses to different kinds of stimuli and so sometimes I'll be…I may observe when they're doing some of those assessments and try to work with the families to address what they are seeing, what the therapists are indicating what that means and then sort of help them process how they feel about that.

I: So, when a family member comes to you and says I've seen my loved one move their hand when I walk in a room and then how do you, how do you know if that's something purposeful or it's just reflexive from your perspective.

R: So, I'm relying a lot on what the therapists are assessing for and what they're…because they're working and nursing I should say both because they're letting me know whether or not that's something that is happening in a way that's in response to commands or oftentimes there will be lots of different kinds of movements that are occurring very frequently that's very common, but isn't really necessarily purposeful, so we'll kind of talk about, usually like when a family member is talking about that I'll say, "Okay, so I'm sure you've talked with whoever the physiatrist is and, you know, what was kind of their interpretation of that movement?" and how did they feel about the feedback that they got? or when they're indicating that they've seen them, like it's really important for us to track any observations that they have along with the rest of the staff. Then just kind of assessing what they think that movement means like kind of sometimes reviewing like this is where we're at and so while that movement is occurring these are the other things that it sounds like that they're sort of looking for and when they see some consistent sort of engagement in this way with whatever, you know, specific movement they would be talking about (inaudible 7:17) visual tracking are they actually responding to

commands or are they actually going to reach towards a target or some of those kinds of things. So, maybe different to kind of move the hand versus intend to move the hand.

I: So, have you ever had a situation where a family member has been adamant, “I’m seeing this. They’re doing this. I’m seeing this, but nobody else is believing me. No one else is seeing it.” How do you manage that situation?

R: Well, I’ve had that happen. It happens frequently, but so I think what we do is we just talk about that sometimes, you know, that we do see those movements and sometimes those things do happen and so we’re continuing to track that and we’re looking for what that might mean and part of what is helpful for us is to see some kind of consistency and that that those kinds of behaviors are duplicated and so that’s what we’re watching for. We still assessing and that’s helpful for them to give us the information. So, we have had family members who have said recently {inaudible/low voice} oh wow we have someone who family was seeing a lot of things and we weren’t getting them to sort of replicate that, just when you emerge some of them started making really rapid recovery family was just reinforced, so I think we don’t try to discourage that I think we try to be supportive of any information and it’s helpful and we’re also sometimes we’re seeing that as well, but we’re not seeing that in a meaningful or purposeful way or maybe we’re not seeing it happen consistently or to consistently the same stimuli and so we’re just kind of trying to look at some important information and we’re gonna continue to watch that. Sometimes that happens at a point where those kinds of similar things have maybe have been happening over a period of time and we don’t see any change in that sort of like we continued to report that you’ve seen those same kinds of things for the last 12 weeks and so it’s kind of recognizing and providing some education on that piece as well

because sometimes it's it maybe the same as it was 12 weeks ago, but they're still saying, but I'm seeing this.

I: So, now let's jump to the patient has emerged and then you get to be involved with them as well, as the family. So, what does that patient look like? How far have they had to come for you to be able to work with them to meaningfully engage with them?

R: Well, we've done some stuff with rancho 4 patients where it's more just rest and relaxation stuff. It's more almost (cultreats?10:28) or working with staff to help identify what our triggers for different behavioral responses so rancho 4 patients are pretty agitated and when we start to recognize a pattern in some of their behaviors certain cares are a trigger we notice, so what can we do differently when we notice that approaching from one specific side can be a trigger or we're doing a lot of like (cultreatment? 10:58) and really just kind of I guess more consultation with a team of nursing at that point. Also, providing a lot of education to family at that point also. If you have somebody who kind of really stays agitated, especially if you have someone who is physically moving and is able to do a lot, but who's rancho 4 and who's not aware all of the things around them and kind of interacting with their environment in a way. They're very fearful. They're not understanding kind of what's going on and so family gets really upset to see them in that state and then if they're physically combative or that can be really stressful for family. "You never hit someone, You should never do that." Well, let's talk about the brain injury, and you know, this is actually normal stage and this is the progression we see this and we're aware that this is indicative of your significant other, child, whomever functionally trying to harm people. It's kind of providing some support around that and then with the patient it's really help them of orientation, helping them process, frequently in the

moment how they are feeling. So, one of the things is that that can vary significantly vary within a session, not just from session to session, but in day-to-day. Also, providing some support for family and the team when there's certain comments that may be made about how they're feeling and how distressing that can be and sort of just provides the normalizing for that them sometimes it makes sense to somebody who'd be really angry or upset or really frustrated and some of that is just normal part of their adjustment and so talking about that and helping them figure out ways to manage. Again remember probably doing a lot of like relaxation exercises and we're looking for strategies that we can do with them in the moment which require a lot of cueing on our part. Then letting the team know, these are the strategies that we're finding are really helpful and effective so if you notice the ABC behavior then this might be a really good way to intervene at that time. And those also evolve over time, so letting them no that is not working anymore and that's kind of actually now a trigger (laughter) so now we've shifted to this.

I: So it sounds like the team communication and dynamic is very important to these types of patients.

R: Yes, absolutely. We rely on one another for a lot of information and things can go really slow and you don't see a lot of change and then when it starts it can still be slow (laughter) or it can be really quick. We have some that you're just like wow, okay if we're not communication from day-to-day and sometimes we have patients that are making those kinds of jumps from day-to-day like we need to stay on the same page on how to best work with the patients.

I: Yeah, absolutely. So, I'm going to try and maybe hone in one particular patient and talk about their behaviors a little bit. So, think about somebody that you had a frustrating experience with.

R: While they still hadn't emerged yet?

I: Ummm….or they have.

R: Well, I think it's easy to get frustrated…I don't know (laughter). I'm trying to think. I think it can be frustrating maybe when there's inconsistencies in sort of how we address things maybe its I don't know if it's frustrated necessarily with the patient, but I think sometimes at that stage it's really just early on up until they're much more advanced. It's really hard to carry over information isn't there so I think sometimes it's more, if we're not doing some of the things we need to do because I think we're just still so responsible for setting limits at that point, educating the family, talking about rest breaks. I think it can be…so it can be frustrating in terms making sure we're all consistent with, I'm thinking of a specific patient who gets agitated really easy, but really loves to have family around, really wants family there which makes sense and that's also very comforting then trying to figure how we can have family be present, but also not stimulating all the time or talking about you know, he needs rest breaks, this is what a rest break looks like and so it's not engaging and talking with them, it's not watching the tv in the room with them, you know some of those kinds of things. So, I think it would be probably more around those issues, I guess (laughter).

I: How about a patient you were surprised by?

R: Well, just recently we had a patient who I talked about sometimes you can see that once somebody emerges it can be there or there can still be a very slow process or maybe a sort of

steady process, but we had a couple admissions at the same time and saw one patient really start to make some gains and we thought oh, this gonna be, this is the trajectory that this individual is going to be on and then we had arguably a much younger patient who completely surprised us and just started once he emerged it was like everyday everything looked very different and just keeping up with him was challenging. So, it's like well wait how is he interacting today and kind of what he's tracking and it seemed like that happened the change if you looked at the notes two weeks later there's no way you would have argued with that. This is the same person. You would've been like, almost unbelievable.

I: Can you give me some examples of things that you were seeing in the beginning and those things that changed?

R: So, from him sort of basically just being able to start tracking visually tracking and being able to just kind of give a yes/no response, some inconsistency there in his kinds of responses. His orientation didn't know from day-to-day where he was and why he was there and what was happening. Then over, again if you looked at two weeks later, which is a short period of time, being oriented to where he is, why he's here and carrying over some details of what he's been doing in his therapies, being able to express more complex feelings, just sort of those rote responses weren't there, like fine, those were like when you started asking like getting more than just a one word response. I mean physically, being able to get up, walk, so those kinds of things. So, having to be reminded what weight bearing restrictions were, so that you know, you don't injury yourself further.

I: So, the other patient that you mentioned, that came in at the same time, you said they looked like they were on a trajectory…

R: Yeah, we thought because there were a couple of changes. It was a slow start so maybe then went back and had there cranioplasty had the skull flap replaced and then came back and had what we thought were kind of couple of good days. More alert and certainly doing some more vocalizing and able to answer some questions about his historical information accurately and then thinking that we were going to see some other kind of carryover from earlier say maybe 10 minutes…and that just didn't change.

I: So, he kind of hit the…

R: Yeah, I mean so he looked different and then we thought, oh this is going to sustain or start to move and hopefully continue to make gains, and really just sort of stayed there and then had some variability where he just had a streak of bad days where he wasn't really able…I mean he just wasn't alert, he wasn't able to engage, so fatigued and then we'd get hopeful again and say oh, he's very alert today and he can consistently answer questions and those kinds of things, give one-word responses, those kinds of things.

I: So, when they came in were they pretty similar as far as their diagnosis, their prognosis? Should they have been on the same trajectory those patients?

R: The injuries were…I don't think they'd be on the same trajectory. Not necessarily because of the injury, but because of the again argued with the one patient who started to make really big gains was young, a young man and in his early 20's versus someone who's in his mid 60's. That's gonna look different. Substance abuse history over the course of a lifetime on the brain,

contributing to, you know what a 65 year old brain looks like and now a brain injury so I think those factors definitely make them not someone we would necessarily think, you know, you're gonna respond the same, but I think it was more so some of those behavioral changes from before he left and had the bone flap replaced then what we thought the first couple of days, wow, this looks very different than prior to that. That's not unusual after a cranioplasty sometimes we just see sort of like somebody has medical issues too that are complicating and once those are resolved then we start to see maybe some more change. So, just those couple of days maybe look like maybe something and then that didn't link.

I: Anything else about two patients that you want to share?

R: No.

I: What about one of your most memorable experiences treating at least cases?

R: Well, for me. I think I've had when patients start engaging on a different level, so because I'm a psychologist. So, it's not my kind of interaction with them, is different in the beginning really sort of delayed so I may know a lot about them and I know a lot about everything that's going on throughout their treatment, but I haven't been someone who's been seeing them every day and maybe even the family is more familiar with me than the patient is. So, I think once we're able to look at how we can, I feel like once we're sort of treating the whole person, that's when it's rewarding for me and that's when I think for me I've had several patients that once I can actually start to manage sort of the emotional adjustment piece and even just if that's prolonged hospitalization we may not even be at a stage where we can manage what about the rest of, you know, what's gonna happen as you leave inpatient rehab. So, I think for me it's just

kind of rewarding to be able to provide the kind of support and be sort of in the role that I feel is why I'm doing the work, so that's what's rewarding for me. It's rewarding to work with the families too providing support, but I see those as very different. Usually, if I'm really engaged with the family then it's not someone that I would see the patient. Oftentimes, there's a different psychologist who then if I'd really been working closely with the family on a lot of their adjustment issues sometimes we may look at whether or not it would make more sense for another psychologist to see the patient so, it's not, I feel like it's not that common, but it happens.

I: Interesting, so I want to go back touch on some of the emotional responses that you mentioned because that's not a piece that a lot of the clinicians ever touch on.

R: No, nor do they want to. No, I'm just kidding.

I: But, even when we go back and we talk about the patient and we talk about their progress and their trajectory and where they've been. You know everything, it's the physical and the cognitive and the medical and all that and that emotional piece doesn't get mentioned for one reason or another. So, I'm interested in your perspective is there a typical emotional response, is there a typical pattern of responses as somebody progresses and emerges?

R: I don't think there's a lot of consistency in that until we get past sort of the agitation. I think there's a lot of frustration. I mean there's the agitation and sort of the fearfulness early on and I think the team talks about what they're seeing a lot and the try to really tie that to these kinds of behaviors or these kinds of interactions or when I do this and I get this response I think this is what I'm see and so we talk about that in rounds, but as far as once patients are starting to deal

more with, they're actually frustrated or awareness is a big piece, really hard. If a patient isn't aware of or insightful about deficits that can be really stressful for them. "I don't understand why I have to stay here." Especially, when you have someone who physically is doing much better than they are cognitively and just can't make that connection. So, I think at point then we're like, kind of within sessions how to manage the frustration looking at belief strategies. I think it's more kind of frustration, irritability early on and then with more awareness and insight that's when you start to see a change and a shift to increases in depression and anxiety. So, that's when some of those kinds of pieces start to become more at play and aware of how different in functioning and how this and starting to predict how this might now affect different roles that prior to my injury, you know, I was a key player in all these different ways and I had roles: a parent and a child, a, whether they're active duty, as somebody who was a servicemember, or somebody who was working a career, roles like am I going to be able to do that. And so that (mend shifts to ??29:00) insight on predicting on how this might impact my ability to do that or do it the way I originally thought I was going to be able to do it. I may still be able to do that, but it may look differently and so, that's when you start to see the mood depression and anxiety which is a pretty appropriate response and then we look at how can we help normalize that, but then also look at what are some strategies to manage it?

I: So, then how challenging is it then when you've got these patients who've got significant memory impairments, when you're trying to…

R: It's really challenging because when you, sometimes you have patients who have from day-to-day don't remember…well some of my patients may not remember me. It takes a while for them…or they recognize me and that they don't necessarily have a negative reaction to me, but

they're not really sure what I do or what my purpose is, so, I'm almost every session sort of reintroducing myself talking about what my role is, so that can be challenging in terms of helping them, like coping skills, so then it's like really putting together when we talk about behavior plans well when you have a patient who's able to follow a behavior plan. The patient is responsible for that. Really, in my role, in patients who are cognitively impaired at that level, it's really looking at developing a behavior plan for the team. How do we intervene? What are the strategies we're using to manage some of these things? What are the cues that we agree upon helping identify how the patient, you know, what's their memory sort of back up plan, if they're writing and if they're able to do that, making sure they're doing or having them summarize, having them do their own handwriting so they recognize…well this isn't mine, I didn't do this, you're telling me I did, but I don't know. So, does that look familiar? Yeah. So, so maybe we've talked about this, maybe we could talk a little more about it and normalizing also how upsetting that could be when they're like so I've seen you how many times and I've told you a lot of information about my life and I don't remember that and you hear to tell me about…that can kinda can be very distressing. So, I think that's challenging in those ways and I think it's challenging because the patient themselves isn't able to carry over any of maybe the strategies, making sure that, you asked about frustration at one point…making sure that we normalize for the team, like this is where they're at in terms of their memory or carryover of information. Our team does a lot, so they're pretty familiar with that, but sometimes any of us can get sort of frustrated within the moment and need those reminders. Sort of like this is really just a reflection upon they're not able to remember and utilize their strategies, so how can we help them in those moments? What are the things that we can offer? What questions

can we ask those so that we might be able to get them to make that connection on their own and if they don’t, how long do we allow that to go before we sort of present options and so that they don’t get so frustrated that they can’t do it.

I: Anything else about this patient population that you want to share with me from your perspective?

R: Nope, I don’t think so. I think it’s challenging right? Because I think you’re doing a lot of work and trying to manage patients and I think that there’s a lot of responsibility on the team and family and what are we seeing, what’s going on, what’s happening from day-to-day? The patient can’t tell you that, so you have to be consistently watching. I think that’s challenging, but…that’s about it I guess.

I: Alright, we are all set. Thank you so much.

Interview 14 Transcription

I= Interviewer

R= Respondent

I: And you telling me to continue is your consent to proceed?

R: Yes

I: Do you have any questions before we get started?

R: No

I: If I ask you a question you do not feel comfortable answering you can tell me to skip it.

R: Okay

I: So the first thing I want you to think about is a patient that you have worked with, started out in one of those states of disorder consciousness, that you were surprised by your initial contact with. So maybe you had an expectation in your mind of what you were going to see and when you went into the room it was surprising.

R: Okay, let me think. I think it was surprising because there's some patients in a minimal state of consciousness that, I was always under the impression that there would be no type of response whatsoever, but sometimes there is some tracking of the eyes or you can see little movements of trying to do a thumbs up or a thumbs down. I think that was the most surprising for me because there is still not in an awake state, however, there's still communicating in a way. Also, I think the amount of movement was different because when you think of that right away if you haven't worked with a patient like that before you think of just laying steady not moving at all, but sometimes they're kicking legs over

the side of the bed and trying to you know roll over and moving their head around and looking around even if they are not processing so that was different too, surprising.

I: Okay, so can you think of one specific patient that you worked with that maybe you heard what they were doing and then when you went in there it was completely different?

R: There's one his name was XXX and he was from awhile ago, but he just when I got the report and we heard he was coming I figured he wouldn't be doing anything of any kind as far as movement. He was actually really active. However, I don't think he ever got to a point of able to really communicate in a meaningful way. That was one of the more unfortunate cases.

I: Okay, so when you say he was really active describe that for me.

R: He moved his legs around frequently and kind of if I remember correctly it's almost like he would move his head around, his neck and things like that.

I: Alright, great. So then can you think of a patient that was on the unit that during their duration of their stay here was surprising to you? Something happened that was really surprising.

R: There's two patients that I can think of that, I'm going to say one is the number one example. He was one that like emerged from while he was staying with us and when he first got to us he was in a really terrible shape if you will, really poor respiratory status and things like that. Then he wasn't much of anything when he came not even opening his eyes, not at all communicating and then he ended up, I mean this is fast forwarding quite a ways, but like walking off of our unit know who his family was, he was able to talk to us. I mean he's an amazing story. So that was really surprising that he had such an amazing recovery because he honestly was in really poor shape.

I: So when you think about someone who has emerged, what does that mean to you? When you use those words what does that mean, describe that?

R: To me and I’m not sure if this the most medically, technically…

I: We’re not looking for technicality.

R: But, for me it’s when there’s signs of communication and whether that’s tracking with a finger and eyes or answering to commands, if you’re moving somethings. Sometimes you’ll say thumbs up and you’ll see just a little flicker, but you know they’re trying to do it. Obviously using their voice would be emerged for me and just communication, that’s the biggest one for me.

I: So now I want you to think about a patient that you felt stuck with when they were on the unit.

R: Like as far as, like man I don’t know what to do?

I: Yeah

R: I would say back with that first patient I was mentioning, XXX, I felt that was with him just because he did communicate so minimally or not really much at all, so I was pretty new to the unit when I had received him as a primary patient. So I just learned so much of, like predicting needs and basing needs off of vital signs and things like that because I couldn’t get anything from him using nonverbal pain scales things like that to just try predict maybe what’s going on and if he’s comfortable and looking for maybe signs of what makes him more comfortable.

I: Can you give me an example? Give me an example of a sign that he was giving that you that you interpreted it as him needing something?

R: A lot of times if he was in pain or uncomfortable, I’ve seen this with other guys in the same situation, which can present much like other people who are in pain, a lot times like sweaty or getting red in the face or just not going to sleep like if you’re working in an evening shift after something like that and if they’re just still sitting there awake it’s kind of like okay what can we do to make him more comfortable, okay reposition him to try to see if there is something that makes him more comfortable. So yeah being

awake or getting sweaty, red in the face, you can tell like those two things…I'm in pain or I'm uncomfortable.

I: And then on flip side, how are you able to tell that you have resolved that problem. Like what kind of behaviors are you looking for?

R: Right! Yeah, I guess it's just the opposite of those things. If they're able to fall asleep, then that means that they are more comfortable. Not really looking red in the face or like they are sweaty or uncomfortable which can happen with some TBIs anyways, but so yeah so just watching out for those types of things.

I: Okay. So when there was a situation that you felt stuck like if there was a patient that you didn't know what to do with, can you describe how you communicated that with the rest of the team?

R: I guess when I felt stuck, initially I really relied on my other co-workers that had more experience than me like my manager and just other co-workers and peers of mine and just say "You know hey can you come take a look at so and so I don't really think he's comfortable, I mean do you think he looks uncomfortable?" You know because at first I didn't really know what the signs were so I would just ask others and that's how I learned those things. You know, oh yeah I think he doesn't look like he's very comfy and let's maybe try this and check back in 5 or 10 minutes to see if it's changed. So that's just kind of how I learned it.

I: Perfect. Alright. So then moving away from stuck, tell me about your most memorable experience working with a patient like this?

R: I think one of our patients we have right now…there's so many…

I: Just try pick one and we can talk about another one too.

R: Yeah, I think…one of our patients…just having them answer a question by squeezing my hand I thought was really amazing because sometimes at first you don't, otherwise you're not sure if some movement are purposeful or not, but then once you know like wow that was a purposeful thing if you ask them your name or are you married to so and so or whatever. That's always really exciting too when you're getting a family member involved is your wife named this to see the excitement for them. It's pretty cool.

I: Can you think of another specific example that was really memorable to you?

R: I can't really think of another one off the top of my head right now. Sorry.

I: No, no you have to apologize. You're doing great. Okay, how about rewarding? Can you tell about the most rewarding experience that you've had?

R: Rewarding. I think working with, there's a patient named X. He was a young guy. He was 21 I think. Before his acts and things he was just a really active happy go lucky guy, loved the ladies like all this kind of stuff and so any who. He came to us for the emerging conscious program and I remember I mean there was no communication with him at all and he ended up, he left in a wheelchair, but he was talking with his family, but he wanted so bad to be independent with like his activities of daily living and I worked with him about brushing his teeth and not myself to do it for him and having him do it himself. He was able to do all those things when he left and I was just so happy for him. I mean happy for everybody, but especially a young guy like that. You know who has so much ahead of him and he wants to maybe find a relationship. So it's like you want him to be able to exert those independent abilities. It was pretty cool.

I: Yeah that is cool. Can you describe what he was like when he first came compared to when he left and he was able to brush his teeth and do some of those things independently?

R: He had no communication at all. He was not able to verbalize follow commands. I remember they thought he had a spinal injury because he wasn’t moving his lower extremities at all. However, he did move his head around a lot because even one of those talking things because I actually went with him to the U of M because they had to conscious sedation to do an MRI and at the time we had an MRI out in a trailer and they didn’t want to do sedation here because they tried doing it before and he would not sit still for anything, but that was his state. He was kind of wiggly guy up top, like I said the lower extremities came later and luckily, he didn’t have a spinal cord injury, but he was just not moving those at all.

I: And then so eventually he started moving them and the eventually he walked.

R: He was walking in physical therapy a little bit if I remember correctly, but he was not walking elsewhere with anybody else. He was able to do squat pivots in and out of bed with us, nursing staff, but he ended up using a wheelchair to get out of here.

I: So what about a frustrating experience? Can you think of a frustrating experience that you’ve had this with patient population?

R: And we’re just thinking of specifically just the emerging conscious patients?

I: Yeah so at least. Yes.

R: I guess I can just say in general, it can be frustrating as a caregiver to not know what to do because there are sometimes like that patient X when I would take came of him in overnight shift, he was one that I would go in his room and he would just be laying there like eyes wide and I would try everything I could to see if I could help him go to sleep in some of way and even give him pain medication, reposition, like essential oils, like all of it and nothing really seemed to work so it, and not in a bad way, but I guess it was just frustrating, “I’m like gosh, how can I make this patient comfortable because

everything that I do isn't working." Is he hot? Is he cold? Should I put a fan on him? Should I do this? I would try all sorts of combinations and for him specifically overnight, I'd go in there and he'd still be just, you know, and maybe he was sleep, but I don't think so. He looked uncomfortable. And I think, I honestly wonder sometimes too if they are just equally as frustrated in their mind I mean not being able to verbalize what they want.

I: Have you ever observed any behavior that makes you think that? Like can you describe something about those patients that makes you think that they might be frustrated even though they can't communicate that?

R: I feel sometimes when…I just can't help, but think it sometimes when they're looking at me, especially if their eyes are open. We've kind of been going through a cycle of trying to make them feel better and more comfortable, and nothing seems to be working, I just can't help but think that that's what they're thinking when they're looking at me. You know like "oh I wish I could tell you." I have seen tears before as well. I consider that a sign of frustration on their behalf or feeling sad so.

I: Can you think of specific time that that happened? When you saw the tears? Like what was the context?

R: I'm trying to think. I'm pretty sure the guy I'm thinking about, usually, has to do with him, is like when we would talk about family, like his wife. Like oh she's here, she's coming, or this. And

I: And then you would see the tears come down?

R: Yeah.

I: Can you think of any other experiences that you've had with those patients that were any of those things that we've talked about? Surprising, rewarding, frustrating?

R: I mean I think the other rewarding thing is that because you're kind of going through as we've already talked about like frustration, and this a that. It's kind of a bumpy road for everybody. I think for, as a nurse, to see them come through and work themselves out of this state is really rewarding and you know because there's definitely times where you wonder and you think because every case is so different. There's times where you wonder and think is this gonna, is this guy gonna pull through? You know, you really kind of don't know and you always stay hopeful, but it's really rewarding to know your patience and your effort and continuing education and your all of it, hard work has paid off and helped this patient.

I: So I like your reference to the bumpy road, so describe that to me like what with this patient population, what do you mean by that?

R: It kind of seems that the emerging consciousness patients that we get generally have a long medical road in front of them as far as we'll transfer them off the unit because they're, we can't maintain their blood pressure or they have some funky labs that come back like their potassium. A lot of times too they come with a craniectomy and then they'll go back into the OR and get that plasty done. So they're very, compared to so of the other types of patients we take care of on the unit that aren't in the ec program, they really, they have a long medical road ahead of them. If that makes any sense.

I: Yeah it does.

R: A lot of, we usually do not assign too many patients to a nurse taking care of a patient like that, just because they're just more complex, as their bodies are trying to figure out what it is supposed to be doing.

I: So I have one more thing I want you to elaborate on. I like how you described working themselves out of this state.

R: Yeah, that’s my impression of it I guess.

I: Yeah, so elaborate on that. I like the way you said that. So I want you to just talk about it a little bit.

R: I don’t know. I guess how I envision it in my mind is that they got into this state by an accident or however and their body was completely thrown off by that and their brain was injured and so now I think what I’ve learned is that the brain takes a lot of time to heal and it needs a lot of time to rest and recuperate and so that’s the biggest thing I’ve learned up here. And so I think we try to provide that environment for them, you know low stimulation and all of that so that their brain can just rest and heal and get what it needs. I think like even the rest of the body because a lot of times it’s usually other injuries as well to the rest of the body besides the head so it’s just helping them rest take care let their body kind of take care of it you know with obviously assistance and medications and things like that, but yeah it takes a long time.

I: Yeah, you’re right. It’s a long road. Okay, is there anything else that I didn’t ask you about that you think is important for me to know about working with this population or seeing changes with them?

R: I think the most important part which is something I tell my friends and family is that it’s not like you see in the movies. It’s not where the patient will just all of sudden wake up and see their significant other and be like “oh what happened and how long have I been out?” “Oh I was in an accident.” You know it’s because most times their not going to remember that or they never do. It’s a very long slow process that comes in stages. It’s not just a boom one day, I’m up talking, walking, it’s just not how it goes. It really is a slow progression and one thing comes at a time, so that’s the one thing.

I: That’s a good way to describe it, yeah in stages.

R: And everybody’s different. No one case is ever the same. It could take somebody one month or can take somebody four months. There definitely hits a point where, but that’s different for everybody.

I: Well I don’t have any other questions for you unless you have something else that you want to tell me about as far as working with this patient population.

R: No.

I: Thank you, so much.

Clinician Interview 16

I: Interviewer

R: Respondent

I: …..you said that you’re okay with answering this questions?

R: Yes.

I: Okay, so what I want you to think about first is I want you to tell me a time when you were working with a patient in a vegetative or minimally conscious state and you were surprised by your first encounter with them.

R: Okay, so surprised in terms of positive or negative?

I: It doesn’t matter if it’s positive or negative, but what you were expecting upon entering the room when you first saw them was not maybe what you saw.

R: In terms of description do you want…

I: Whatever you can describe so if you can think of a specific patient, just describe what was surprising about that encounter.

R: Okay.

I: I know it’s a different way of

R: (Laughter) It’s vague.

I: (Laughter) I know, it’s vague on purpose.

R: So, there’s lots of different examples. One specific example would be a patient that we had taken care of in the past had more of a mild to moderate traumatic brain injury and then returned to us several years later with more severe TBI with minimally conscious state. So surprised at how much worse he looked and how much worse he looked even then based on chart review just with his medical complexity and his clinical presentation. He just need a lot of, better nursing care, so he wasn’t in very good shape at all. So kind of surprised by how bad he looked at that time. So that would be one example.

I: So medically from like a (skin? 30:29) perspective, those types of things?

R: Yeah, medically and neurologically I guess too.

I: Okay. So can you describe a few, if you can think about it, can you describe maybe neurobehaviorally what you expecting to see when you walked in and what you actually saw. Like how it was different.

R: I think that I was expecting him to be a little bit more responsive and he wasn’t so.

I: So he wasn’t responding to like if you gave him a command to follow, did you have a, was there any response at all? Or was it…

R: Um yeah, I think he was, if I remember correctly, this is several years ago. He was responding to some, attempting to respond to some commands, but not consistently. His responses were pretty variable.

I: So can you think of, sticking with the idea of surprise, can you think of patient that you worked with that at least came in in a vegetative or a minimally conscious state that throughout their stay at some point was surprising to you.

R: I think sometimes the change is so slow over time that it’s not like an immediate responsiveness change that surprises you. So it’s kind of slow over time, but definitely several I’m just thinking of one more recently because I’ve been covering so that’s in my head now. These are older examples. So on bedside exam day-to-day does not always show a lot of responsiveness and appears very impaired, but so with therapy on occasion we’ll have improved command following and yes/no responses and now considering for power mobility, so definitely very shocked at that at the changes that he made. I was not expecting.

I: That’s neat, those are the good stories to tell. Yeah?

R: Yeah

I: Okay, can you tell me about a patient that you might have worked with where you felt stuck.

R: Okay. So I’m just clarifying stuck in terms of treatment planning? Or?

I: So it could be treatment planning or they seemed stuck in there progression or…

R: Okay, or are you speaking more of like placement and things like that?

I: No, not placement, no. Thinking about really more the patient and the neurobehaviorally maybe?

R: Yes, so especially if patients aren't improving or changing, you definitely kind of begin to run out of ideas in terms of any interventions you can do to try and improve their functioning and so definitely there's patients I can think of.

I: Can think of a specific example? I know it's been a few years since you've been in inpatient, but can you think of a specific patient that was like that and describe maybe what, why did you feel stuck with that particular person?

R: One example would be a patient in a vegetative state, so not really showing any purposeful activity or responses at all, very severe injury and autonomic dysregulation and it was developing significant (? 25:41) classification everywhere so, but still medically complicated so very limited interventions you could offer and he was here for our 12 week emerging consciousness program. So just showing no change at all in terms of level of responsiveness and ongoing medical complications. So just feeling like stuck in terms of not able to provide any interventions that are going to help this situation.

I: So how did you navigate that situation either with your rehab team or even with the family?

R: With the rehab team we have a pretty strong team that follows our emerging consciousness protocol, so I think everybody was pretty much on the same page with where he was at in terms of his complexity and his lack of improvement. So that was, it was what it was. There wasn't a lot of team intervention that needed to happen there, everybody still worked together. I think just tried to deal with medical barriers that might be limiting his progress. In terms of the family, that particular case had a more complex family dynamic. So, a young spouse and then mom and dad who were preferably involved. So we were working with all of

them, but spouse was here, I would say more than other family and so I think we met with them. We do frequent meetings with family. We do family collaboration meetings early on where we talk about brain injury education and prognosis and then we do frequent meetings throughout to talk about where they're at and any progress or lack of progress and things like that. I think we were maintaining pretty constant communication throughout. I would say that there is always some misperception with how the family feels the patient is doing versus how the team feels they're doing. So I just thing we continued to provide that education for them in terms of where they're at and prognosis and then eventually got to a point of talking about end of life things and decisions and kind of those decisions that they may want to consider going forward. So I think that's difficult, but I think with close collaboration with the family in a positive way it was…we were able to navigate that, but certainly helps to have social work support so closely involved and family psychology involved and have everyone working together.

I: Absolutely. Okay now let's talk about a memorable experience that you had working with this patient population.

R: So I had, do you want us just to focus on just disorders of consciousness or TBI in general.

I: Disorders of consciousness, yeah, and I know, I realize that sometimes people come in in a disorder of consciousness and then they progress and that's okay, but I want you to think of a memorable experience that at least when the person came here they were in a vegetative state or minimally conscious state, yes.

R: Okay. Yeah, so there would be several I would say. One is a younger man who came from Iowa after a sledding accident so he was severely injured and enrolled in our disorders of consciousness program or emerging consciousness program. And emerged, we had to be creative with deciphering his emergence because of some underlying apraxia and aphasia that may have been there, but he emerged and I continued to improve had some agitation and eventually now is able to live independently.

I: Oh wow! That's amazing. That's awesome.

R: Yeah, but it's been over, that was probable eight years ago. So it's been over a long course of time that things. So with a lot of impairments and permanent deficits, but able to live independently, not work, but yeah.

I: So I like how you mentioned deciphering his emergence. I know that's the challenge sometimes. Can you describe that a little bit more so

R: In terms of that patient?

I: Yes, yes.

R: Yeah, so we're usually looking for them to show some kind of consistent either yes/no responses or following commands or using an object in a functional way. Something objective like that, but you know many times if there is other sensory impairments hearing or vision or if there is some aphasia or apraxia that can be hard so I think we're still looking for those basic things to be accomplished to show that they are emerging, but just that in each discipline they are seeing that in consistent way. So I would probably be stretching my memory to try and

remember what we did, but I think just showing that with speech, with OT, and PT that they are showing those consistent responses throughout.

I: And I know you can't remember the specific details, but can you give me kind of a broad picture of what maybe you were seeing with him that was maybe muddying the waters so it was hard to tell if maybe he had emerged or not?

R: I think because there was a language piece at that point that in terms of following some commands or answering yes/no questions there was not always consistent or it was kind of questionable what if he was emerged or not and I think as he continued to progress he kept improving and so then we were able to be able to some of those things or he was able to do some more automatic behaviors in terms of functional object use that showed he was using an object purposefully and objectively and intentionally. So using more of those parameters.

I: So it wasn't an "Aha" that he had emerged, it was more of a gradual determination over time?

R: Yeah.

I: Alright. Okay, and then what about a rewarding experience or do you have another memorable experience? Do you have any more memorable experiences that you want to share?

R: Yeah, I mean. I think we have a patient, I still follow in the outpatient setting that was one of our emerging consciousness patient, so probably from 2012 and he again was admitted to our emerging consciousness program and he's another one who has had a slow improvement over

time, so he was fairly, was not improving during his inpatient stay, during a 12 week stay. So kind of remained in more of a vegetative state in that time of discharge from us and trach and peg (full cares?? 15:45) with pretty significant seizure disorder. So he was able to discharge home with mom with a lot of support and then he has been followed at home and in our outpatient clinic and over time he's improved to, he can feed himself. He can talk, communicate, and has improved function. So he has been rewarding in the sense that he has developed a lot of improvements in terms of function.

I: And you said that when he left here, when he left the rehab unit here, he was still in a vegetative state?

R: Yeah.

I: Yeah, that's incredible.

R: It's sometimes a slow, subtle, cumulative changes over time that kind of then add up to just these slow functional gains over time.

I: Right and how long do you think the period of time was from when he left here until he got to the point that he was able to communicate and feed himself?

R: He was with us for a while just with all the medical complications so probably at one point during his hospitalization he decompensated it was in the ICU when we were talking about end of life. So to think back on that was kind of just interesting. He went to a nursing home first, that didn't go well. He came back to us. We did caregiver training, then discharged home with mom. Then he got his trach out, so it's all these things that just added up with changes, so it

was probably like six months to a year that we started getting the trach out and then ongoing medical stability. So as he became more medically stable that’s when you started seeing more of the functional changes. Removing the trach and his respiratory system…

I: Sure. Sure. And living at home probably helped too

R: Yeah

I: Yeah. Okay, can you think of a frustrating experience? Again, and I’m not thinking of like a discharge planning frustrating experience, but like either whether it was frustrating trying to figure out what this person was able to do.

R: You know I think sometimes there’s a lot of frustration when someone’s not improving and that can be, not necessarily frustrating, it can just be a little bit disheartening, I think for a team when you’re working with someone for so long and they’re not improving. In terms of frustration, I think sometimes there is when family is at a totally different place than the team, like very different.

I: Like with expectations?

R: Yeah. And so pushing the team to do things that aren’t appropriate or having those expectations and then many times because their idea of how the patient is doing and their expectations are so different than reality, then they get frustrated and angry with the team too. So sometimes there’s that anger that can be directed at the team when someone is not improving.

I: Can you think of a, and you don’t have to get into the family dynamics, can you think of a patient like that that was not improving and there was maybe a disconnect between the behaviors that the or how the team was interpreting behaviors and how the family was?

R: Yeah, there’s a couple. One was of again a patient admitted to the emerging consciousness program I think after a fall. So had a severe TBI frontal craniectomy and was still a lot of cerebral edema was definitely vegetative state. He couldn’t open his eyes so it was hard to always tell if it was the coma or vegetative state because he had lid weakness and swelling. So he was, did not make any changes at all. The prognosis was very poor and family just had a lot of misperceptions about thinking he was having responses, that were not there. And then just, you know feeling frustrated with the team as we’re providing that information or trying to provide that information or discharge planning because you know many times family feel that you are giving up on them when you start to do that. I think that was a frustrating case for the team and probably for the family too just because there such a disconnect there. And no ability to, just a family that was in complete denial and so there’s just no ability to provide education.

I: Can you think of some specific responses that he was having that the family maybe interpreted differently than…?

R: No, he really didn’t have any. I think if, thinking back of, you know, if he did have a spasm or you know something like that it would be like a purposeful movement or maybe they felt like he smiled or you know like it was facial grimace or something like that or a grunt that maybe was a cough.

I: And they would interpret that as purposeful behavior?

R: Yeah. Another example was just a pretty severe patient was here for our emerging consciousness program. Went to a group home. He did emerge. He was complicated because he was blind and had severe hearing loss, but could follow commands and answer simple questions, but unreliably. We did a lot of cueing which is kind of answer yes/no, but not reliable all the time. But was able to do some transferring, so he had some functioning, but needed 24 hour care. A lot of physical and cognitive behavioral deficits and so he continued to work with us in the outpatient setting and so that was a really frustrating case for the team because she was pushing, mom was pushing the team with goals of running a marathon, or just very high level tasks, returning to college, things like that. And the team would just not know where to go with those responses because she was not, just in denial of where he was at currently in the prognosis.

I: I would like you to go back just for a second and talking again about the emergence into consciousness. We have our definition or whatever. When you see a patient emerge, when you consider a patient emerged, can you describe that to me? Don't tell me the definition, but describe that, like what your, what you see when you know that someone has emerged into consciousness.

R: I mean I think it's more of a gradual thing. I don't feel like one day you walk in and they are emerged because we're doing serial daily exams on the person and nursing is getting a 24/7 view. You have all this information that you are gathering all the time so in my experience I would say it's more of that taking all of that input in and it's not kind of a black and white thing. So what I would typically like to see when I'm following patients is you know that they are beginning to show some localized and purposeful activity. We might start to see that first, that

some sort of intentional motor cognitive behavior and then that that’s consistent. You’re seeing that consistently and then that that’s kind of building into even more than that. Either following a command, like a yes/no or whether that’s nodding or thumbs up or thumbs down. So something consistent. Following a command or being I guess in terms of that purposeful movement, we’d just be building on that in terms of being able to manipulate an object or throw a ball or something like that.

I: Can you think of someone recently that you’ve seen that that you were watching over time and eventually decided that they had emerged into consciousness and how you made that decision?

R: Well I haven’t done inpatient for the last two years so I’m not doing that program anymore, but let me think back to cover…with the last couple of…like I said I think it’s more gradual and I think the team, you’re relying on your team a lot to do some of that serial assessment and so taking input from them in terms of objective things they’re testing that when they’ve done this consistently for several days in a row then we’ll document it.

I: When you’ve decided that they have emerged.

R: Yeah.

I: Okay.

R: Yeah, so I would say for the most part that’s usually yes/no consistently or following a command.

I: Okay. Alright. Excellent. So those are really all my questions, but is there anything else that you wish I had asked about working with this population or that you would like to share with me about your experiences working with the population?

R: I don't think so. No. I think I was anticipating more language that we use with family.

I: Ohhhh. Right. Okay.

R: Around emergence was my interpretation of what the study was about.

I: Sure. I mean, so what, so we didn't talk about that though, when you're talking to a family about that kind of progression how do you explain that to them?

R: Yeah, so that's what I was reading a little bit about Michelle had sent was kind of studying how clinicians communicate with family about emerging consciousness.

I: And that is partially what we're doing, but tell me about that then. Tell me. Yeah, so tell me about how you do communicate that.

R: Yeah, you know I think I like to use some of the language I mentioned to you and really trying to be open to families input in terms of what they are seeing and never downplaying or denying that, but also just trying to encourage, or repeating to them many times that we like to see consistency and what we're looking for is these kind of purposeful behaviors of intentionally interacting with your environment and in that those are good signs and so we want to keep looking for that consistency with that and building on it. So using more of that type of language.

I: And then when you have a family who isn't understanding, how do you, do you have any language that you use or do you have any methods that you use to try to help them to get in line with what the team is seeing?

R: It depends. I think the family's readiness to accept that and so, what I learned over time was that you can't push that process and force it because they get angry and they lose a lot of trust and faith in the team. So you have to kind of accept what they are telling you and say well the team and you know just kind of reiterate the same things. Also, if there are any physiologic reasons for the response there are seeing could be spasticity, spasm, or grimacing, things that pain could be accounting for some of that, just making sure that we're talking about those things and how they can appear as responses, but be more physiologic or reflexive things that are happening and not necessarily purposeful.

I: Those are hard conversations to have right?

R: Yeah.

I: Well that's all the questions that I have.

Clinical Interview 17

I: Interviewer

R: Responder

I: …and you're willing to participate?

R: I am willing to participate.

I: Awesome. So, the first thing I want to is, I want you to tell me about a time when you were surprised by the first encounter you had with a patient that was either vegetative or minimally conscious?

R: Alright, well this was several years ago. I was working at a private sector hospital and we were doing coma stimulation back then and there was a young woman who had been in a motorcycle accident. I had seen her on Friday and she was in a vege- similar to a vegetative state her she had eye opening and sleep cycles, but she was not really responding maybe she had some startle responses, but when I came back in Monday morning, she came to my office sitting upright in a wheelchair and she was responsive and that was one of my earliest memories because it was so shocking and it's so not what you expect to see, but it's something that I will remember.

I: Yes, forever right?

R: Yes.

I: And it's so exciting when that happens.

R: Yes.

I: That’s a great example. Can you think of an example where you had expected to see something based on either what had been reported to you by the team or maybe what you reviewed in the chart and when you went in for the first time to see that person you saw something completely different?

R: Yes, that happens frequently where I will read a chart and I will read about the patient really doing all these things and then when I see them for the first time in this new setting, they’re really not doing all those things and it just I think takes a while to be able to figure out how to draw those responses out of patients and sometimes they’re responding when family members are present and not oftentimes patients will respond more or initially with family members and familiar voices and whatnot and to therapists. You just don’t get as much. That happens more than the other way around when I walk in I’m seeing all these things where it said they won’t respond.

I: Right. So, it happens more often that you that it says that there were things that you were seeing things, but when you walk in, you don’t see them right?

R: Right.

I: So, can you think of a specific patient? Can you describe a specific patient like that where what you read in the chart was one thing, tell me what you expected to see and then tell me what you saw?

R: Right. This is a patient who had a severe TBI two and a half years ago and we had seen him here about a year ago for some, in the EC program, for I don’t know how long, but he was minimally responsive at that time. I would say he was minimally responsive, but not responding much. He came back for a two-week respite stay. He’s here now as a matter of a fact and he had

been receiving great stimulation from his parents at home and getting some speech therapy in his home and I had heard how much progress he'd made and he's coming back now for to work on maybe finding an AAC system like event computerized sort of system. He had been differentiating between objects and pointing and grasping. So, I expected to see more than I did when I first went in to see him. For the first few sessions, he really didn't do much with me at all. It took me awhile as I continued to work with him I started seeing inconsistent responses, but they were really very inconsistent and…

I: Can you give me a specific example? Just think of one thing that you asked him to do that he didn't do or…? And how he responded.

R: Right, and I responded I actually called the home therapist with him in the home and she was having him showing him two objects or holding up two objects and say, "Grab the orange one or grab the green one" and he would do that for her. I have not been able to get him to do that for me. He will grasp and release on the left, but he doesn't for me so.

I: When you ask him to do it, there's nothing? Or is there a response at all?

R: Not, no, he doesn't do it. He'll usually like grasp the one on the left, the item on the left because he's got a big right field.

I: Oh, okay.

R: So, that's one example. I'm trying to think of some others. Often, I think, not often, frequently patients will respond to their parents and I try to reproduce that and it just doesn't happen with me. I can't think of a specific example. I know a lot of family members will think that there's a yes/no eye blink and they'll say they're communicating with me by blinking. And I just can't get it, I can't tell.

I: That’s frustrating. The eye blinks, yeah, I know.

R: Yeah.

I: Can you think of, now think about a patient that you worked with that at least came in in a vegetative or minimally conscious state that during like the duration of their stay, something about their recovery surprised you.

R: Okay, yeah. What’s coming to mind are the recent patients that I had.

I: Sure, yeah. That’s easier to remember, right? Whatever works.

R: I had a younger gentleman who had a bifrontal craniotomy and he was in a minimally conscious state, but after he had his flaps put back in, he started perking up. He emerged from and I can’t say that, you know, if it’s from that or but he came in, he was so impaired for so long. He actually emerged and was walking a talking when he left. He actually went to Hines.

I: Ohhhh, that’s awesome. That’s awesome. Okay, so thinking about before the craniotomy, describe to me what he was doing.

R: Like what…

I: Can you think of a typical therapy session or something and describe some of the things he was responding to?

R: Yeah, he was following directions very irregularly and very inconsistently. He was nonverbal. He was not taking any PO at that point. So, it was a lot of getting, working on visual tracking and scanning and I did a lot of co-treatment with physical therapy so they would be doing some physical things and I would sit in front of him and try to engage him.

I: And then so, after he had the craniotomy, then describe, I know there’s a progression, but maybe describe the first couple sessions after he had the craniotomy. What kinds of changes did you see?

R: Well, he was starting to follow directions more consistently.

I: Can you give me an example?

R: Like, turn your head or kick your foot. He would start kicking his foot, kicking a ball. He would start like pushing things away, if I would come up and do some oral, stem and so a lot more consistent with the localized responses which then kind of went, he emerged then. We just progressed right along.

I: Awesome. Okay, we talk about emerged a lot. People emerged from minimally conscious state or they emerge into consciousness. So, describe that with him to me. How did you know? How do you know that he emerged?

R: Well, I can’t specifically remember that, but we use the JFK scale and so we want them to be able to use two objects functionally or respond yes or no consistently and reliably to those six questions. I have a feeling with him it was the object use.

I: Okay, Okay, but you can’t remember?

R: I can’t remember.

I: That’s okay.

R: I’m sorry.

I: No, don’t apologize. So, those were good examples, now I want you to go to a different situation. I want you to think of a patient that you worked with, that you felt stuck.

R: Oh. That happens, normally my experience with that is with the older people. The one that I'm thinking of was stuck in not a vegetative state, but a minimally conscious state and he stayed for our 12-week program and so it was pretty much the same so I would go in and my goal was to try and improve consistency of response. So, pretty much I would go in and everyday go through my set of things that I was doing just to work on that consistency and there really wasn't any change and so then it's just a lot of educating the family.

I: So, talk about, tell me about how you communicated that lack of change then to family. How did you describe that to them? How do you talk about that?

R: Right. I like to have families observe and I will let them know this is what, these are the things that I'm looking for. I talk about localized versus generalized responses, so I give a lot of education about that and specifically if it's a visual scanning goal we'll talk about how well they're scanning. I would sometimes use percentages with family members if they want to know specifics, but I'll break it down into the sensory modality area and just talk about, you know, how much of those responses were generalized, how much were localized. I think with a lot of education family members can see themselves.

I: Can you think of an example of patient that you were feeling stuck like that and you were able to help the family to see that? Or they started to understand those responses better?

R: A specific…a specific?

I: If you can. If you can think of a specific patient that you were stuck with and maybe how that navigation with the family went.

R: Right here and now, I can't think of a specific patient, but I can think of kind of my particular approach.

I: So, you can describe that. If you can’t think of a specific patient, then describe the approach. I mean you did that a little bit.

R: So, ask the question again. I’m sorry.

I: No, that’s okay.

R: Differentiate that last question from before.

I: No, I know. So, what I was asking you about was you were talking about how the family sometimes takes them awhile and some education to understand that there is no response there, right? The quality of the response. So, I was asking you to give me a specific example of a patient that you felt stuck with and how you navigated that with the family.

R: Sometimes, I will actually have the try to illicit some responses so that…I’ll say to them, you know, they’ll tell me they’re getting responses and what I say to them is, that’s great can you kind of write down or let me know what you are seeing? So, I’ll have them keep a journal or list of the responses that they’re getting so that I can work on recreating those responses with the goal of increasing consistency with being able to do that with a number of people.

I: I like that. That’s a good idea.

R: Because a lot of times patients will response better. I like to start with what the family is getting.

I: Sure, because they’re there the most.

R: They’re there the most. Absolutely, they’re the ones that know the most about their loved one.

I: Now, I want you to switch and tell me the most memorable experience you have working with a patient like this who at least came in again, started in a vegetative or a minimally conscious state.

R: I always have…I have so many great memories because the most exciting thing is to see a patient go from a vegetative state to emerged. I mean that is just so exciting, right? And so, some of mine are about, the patient has their first real meal which is always really exciting. Or even their first little snack.

I: Can you think of a specific patient like that and describe that?

R: Yeah, I can and the patient, it was a pureed or pudding, chocolate pudding. I had him choose which one and he was able to indicate chocolate pudding and he ate it and his mother was in the room crying. That was a very memorable experience.

I: That's awesome. Yeah. Can you think of another? Either another eating example or just another memorable experience with treating this patient population? Whether it's a small moment in time or over a duration?

R: Well, one of my patients that, this was a long time ago, and it was here he started to functionally communicate by using his tongue. He was very very motorically impaired, but he had tongue protrusion and lateralization so, we, the only way we could get a yes/no response was using his tongue. It started just with yes/no…right…right for yes and left for no and he did that, but it progressed to using like a low-level communication alphabet board with different symbols along the different axis so he would do two mouth movements and then you'd have to find what letter he used...

I: Oh wow. Okay.

R: For what he was talking about. That was a memorable experience.

I: That's amazing.

R: That's never happened before. That was my one and only.

I: Right. Yeah, I don't think I've ever had that, that is amazing. So, along the same lines, your most rewarding experience working with someone who came in a vegetative or minimally conscious state.

R: Well, I had one young, very young guy probably 20 years ago. He eventually improved to the point where he could take classes at the college level. Another guy, I was thinking about wrote a book about his experience recovering from brain injury.

I: Wow. Okay.

R: He later came and brought me a copy of that book.

I: Ohhh, that's awesome with, um, did he sign it for you.

R: He did, yep.

I: That's amazing. I like to hear those stories. I do. Okay, so now I want you, now we're gonna go back to more frustrating… I want you to think about a frustrating experience that you've had.

R: I get a little frustrated with the 12 weeks is a long time to work with a patient who is not really changing, but right now I have a gentleman who is so variable. There are a few days, like occasionally he'll have great days where he's doing a lot, but it's been the next day very minimal. So, just so the length of stays sometimes frustrates me when there's not any progress. I'm doing the same thing every day and it's hard for the families to see that. That nothing's really changing, so, you can see the frustration building with the family and so in general.

I: So, can you describe what this patient that you are seeing now, with the variability and performance, can you think of maybe one or two things that you do in therapy and describe that variability, like what you see one day versus the next?

R: This particular patient is (retrofied??? 7:13).

I: Oh, okay.

R: But I've had other patients that have been very, like just can't pin point a specific one.

I: Can you think of another example of any of those experiences that we've talked about. So, another example of a patient you worked with that you felt stuck, that was memorable, that was rewarding, that you can describe to me?

R: I'm thinking…there have been so many.

I: I know it's hard, right?

R: I had one gentleman that was cortically blind and that made things very difficult and because there were limitation as to what we could do with him.

I: Can you think of some of the things you did in therapy and how he responded to them?

R: We did a lot of just auditory things and I think there was anxiousness on his part which made it difficult. So, we initially start with just ringing bells and is he local-- turning his head to localizing even though he can't, he can't see and then progressing to yes/no questions and simple commands.

I: With the yes/no questions, did you use thumbs up/thumbs down or what kind of system did you use for him to answer yes and no?

R: It was nodding.

I: Nodding, okay. Oh, okay. And then for commands can you think of maybe a couple of commands that he, that you would give him consistently and how he responded to them. I don’t know if he was following commands right away or…

R: I can’t remember that specific detail, but another challenge with this population is usually the patients are very motorically impaired, so, trying to work with, well luckily, we have a rehab engineer here so we can work closely with him and try to see if switches or something like that could be beneficial to patients. I used a toby with one patient. He was motorically impaired and we were doing eye gaze, working with that. It was really very difficult because it requires a lot of attention and concentration. So, that wasn’t really that successful, so, part of the frustration then too comes to the limitations that the patients come in with.

I: Yeah, so when you mentioned that, it jogged a question in my mind…So how do you, can you think of a patient that you worked with that was motorically really involved that you thought had emerged and how did you decide that then.

R: And, you can get patients that are emerged, but are aphasic and (Tan???3:02). So, that’s really a good question. My colleague was telling me the story about one her patients who she decided he was emerged because he was playing tic, tac, toe, with her and putting, spontaneously he could do that…

I: Do the X’s and O’s?

R: Yeah.

I: Okay.

R: But he wasn't really following directions and he was apraxic I think too. So, it was hard to tell, but in my situation, I can't think of one right off hand.

I: It's hard to think of specific examples, isn't it?

R: Especially, when you're (laughter).

I: Yeah, I…so can you think of anything else either patient experience or anything else about working with this patient population that I didn't ask you, that you think I should know or that you would want to tell me about?

R: Yeah, I just like to with this population, I think it's really helpful to work closely with the families and luckily here we've got the Fisher house and a lot of times the families are right here, so I like to use the family to get as, learn as much as I can about the patients that I can… whenever I'm interacting with them. Talk about things that they're interested in or I like to use pictures that they have in the room, anything that's familiar. Like, one gentleman was a big hunter so we were using like dung as a smell or using coffee. Somebody else loved coffee, so we would try to use coffee smell or even just a little taste, if we were working on gustatory stuff. So, I think that's really important.

I: I agree and then…

R: See what's rewarding to the patient.

I: Yes, and that's also important because then that gives the family something to be involved, it gives them some involvement as well. I think that's always important.

R: I worked with a few younger guys that responded really well to my students who were young and so, my theory is whatever worked whatever gets the patients more interactive that's what I'm looking for.

I: Right, absolutely. Well good. Well, I don't think I have any other questions. You gave me some great examples and we're done. Unless, you have anything else that you want to add.

R: I don't!

I: No? Alright.

Clinical Interview 18

I: Interviewer

R: Responder

I: ……Do we have your consent to continue?

R: Yes

I: So, as I mentioned we're talking about patients who are in this disordered state of consciousness so I want you to think about any of those patients that you have worked with in the past and I want you to tell me about a time when you were surprised by one of your patients.

R: It's fun when they start to emerge or respond to behavior that they haven't been able to in the past and it shows that they're maybe starting to improve or heal or get to the next level. So, I'm working with someone now who when she came in was in a minimally conscious state and we weren't getting a lot of responses from her, eyes were open, but movement and not talking and when I handed her a pen and asked her and we've been told that she could write her name, but it didn't seem very likely and when we had tried in the past that we hadn't been able to elicit that. So, it had been a couple of weeks since she'd been here and when I tried again, things kind of clicked in and she was able to copy somethings with me it was sort of fun and we got it on paper and save it in her file.

I: So, what was surprising about that?

R: The fact that she was kind of able to respond, able to do something functional that she hadn't been able to in the past.

I: Was it something that you were expecting that she would eventually be able to do?

R: Hoping that she would eventually be able to do it, but there's no guarantee. You certainly see plenty of people that don't improve. They don't get better. So, you can always hope.

I: Did she go on to continue to make progress?

R: Yes.

I: What kinds of things was she starting to do?

R: So, she's still currently here and she so she's moved into the minimally conscious state and she moved emerged and she's walking, she's talking, she's eating, she's playing card games with me and talking me out of my (3:51??). So, she's really come a long ways and she still has a ways to go, but right now she's still making progress.

I: So, you said she moved into the minimally conscious state so, what would you have classified her when she first got here?

R: She may be minimally conscious state she may have come in minimally conscious state it's just that early on when you're trying to get to know them, we weren't getting a lot of responses from her, so the combination of just the exhaustion of travel and the new people, they where she'd been before if I remember correctly besides the writing her name I think that was about the only thing that they had that would have led me to think that she was at least minimally conscious. So, she wasn't doing much tracking her response or following commands, so it gets

a little gray when they first come that often that is response is what we’ve been hearing. Like I said, I think that’s a combination of new people getting to know someone and being tired and kind of overwhelmed by the transition.

I: Tell me about a time when you had a frustrating experience with a patient in a disordered consciousness state.

R: I don’t know how frustrated I get. It’s challenging so, we were working with someone that was rancho 2 or 3 probably more like 2 and he started to decline so he wasn’t as responsive and anything that you tried would set off shaking and yelling, screaming so it was a challenge to figure out how to keep working with him without setting him off and felt bad for him that he’s yelling and screaming and obviously scared or in pain and so the challenge was to try and figure out what’s gonna help and how can we interact. Is there a way to interact with him without setting that off? I don’t know that I get frustrated it’s just always a challenge to figure out what’s gonna help and what’s not working. Another frustration or another challenge is that you get different reports so family will often tell you what they’re doing and we haven’t been able to elicit that yet. I initially tried, you have a big snatch between what the team is able to elicit sometimes than what the family is. I usually ask them, please tell me what you noticing and what you’re seeing and tell me about that because that’s gonna be something that we’re gonna try to duplicate and get them doing more and more often. And they’re likely to respond to family first, so when your with them that can be reading body language better and stuff. So, that’s fun to elicit and try to get family’s…the challenge is when it seems much higher that what we’re doing with them or at and so we have to think of how is it an interpretation thing. Do they need more education for the family on what they’re noticing and also is there a way to

duplicate maybe, I just need to catchup, maybe I’m way behind and this person can actually do more than I give them credit for because I haven’t been able to so often we’ll ask if we can do a co-treat with the family and see if they can do the administer the stuff and go from there. But also we talk about how that just (?? 8:36) gonna happen. They may do it with sometimes they’re with the patients a lot more often and (?? 8:47).

I: I think this concept has been coming up a lot with families are seeing one thing and then we don’t necessarily see it. So, can you give me some examples from your experience of maybe pick a specific patient where family has said I am seeing XYZ some specific examples and then maybe you didn’t see it or how you would have helped.

R: So, we’re working with a gentleman who came in as a rancho 3 and he wasn’t following directions. We couldn’t get him to move much of anything and co-treating on Monday when I came in they said the family reported that he was responding, I said oh and that he was reliable with yes/no responses. So, that’s like a huge change from what we’ve been able to get on Friday and my response was that’s fantastic and show me what you were doing. He ended up having an appointment at an eye clinic so I asked his fiancé who figured this out? And had been doing it with him on the weekend to come with me. I usually go to the eye appointments and I try to make it to assist with communication and with reading signals and that kind of stuff. And so, so I had her show me what she was doing and we talked about looking at accuracy of his so it’s really easy to say yes to everything or she didn’t know how accurate, she felt like he was accurate so then I was like let’s challenge that a little bit. So, then I started asking opposites are you 25, are you 32, doing this, doing that. He was like 80-90% accurate. So, I was like I totally agree with you and that’s a huge shock I think. He always likely was able to do more of that I

mean he was understanding more than he could show us and finally got enough control of that he could (11:15?)

I: So, was he demonstrating any of those behaviors before the thumbs up were there any kind of response?

R: Not really. A little tone movement or something, but nothing consistent and it’s like you know he could be move somewhat well, but not consistently and so he jumped up a level over the weekend.

I: So, where did he go from there? How did he progress from there?

R: Well, eventually he was able to talk I think we went to AAC devices with him. Eye tracking those were hard eventually he got to the point where he could make some sounds and then words we could ask him to repeat, we’d ask him yes/no questions to clarify stuff, but a combination of talking and the foul? 12:26 Or eventually got more movement. So, he progressed that way.

I: Can you just briefly describe AAC for those who aren’t familiar?

R: So, ??? for alternative communication is another way to communicate so, we used probably like (12:47??) like a computer system and we could send output switches where you can touch the switch for yes or no, you can scan when you get to the thing you want you can hit it. What we use a lot at the lower level is eye gaze. Just look at what you want and so we can do just a trace toy a yes or a no and if they stare at it long enough then indicates a yes or no and then you can branch that to beverage choices or lights on or off or whatever else you want.

I: I want you to think about your patient and tell me what your most rewarding experience treating one of these patients has been?

R: When they emerge from a coma or a minimally conscious state and they are able to communicate and keep improving. So, we’ve got two right now that we started working with when they were rancho 3 minimally conscious and they’re both walking with help maybe not very far and they’re both talking. It’s really provides hope with the next people coming through, you know not, there’s plenty of patients that haven’t emerged and its always a disappointment for the family and then also for the team (14:22??)

I: Can you maybe pick on specific patient for me and describe what they looked like when they came in. Kind of what they’re doing behaviorally and kind of take me through their progression.

R: I’m not how to answer your question different from what we’ve been talking about.

I: Yeah, so I want you kind of start, you know when they first come in, what kinds of things are you trying, what kinds of responses or lack of responses are you getting at the very beginning and how your approach might change as they start to change as their responses start to change and what that looks like?

R: So, when we first start we’re looking at how they might respond to their environment and is there a way they communicate with them. Are we able to interact? So we’re looking at can they look at us when we call their name, can they follow us in the room, sufficiently (???) with sensation and motor responses so. I’ll introduce myself and try talking to them and see if I can get them to pay, to look at me, how long can they look at me, if I move do they move with me. Can they follow directions? If I like, I might try an ice chip and see do they open their mouth, do

they try to chew it, do they swallow, do they open for more. If we clap or make a sound do they startle, do they turn towards that sound, which are different smells and tastes. We do the disorders of consciousness also and jfk. So, I see them on my own and I also see them in co-treatment with either OT or PT. So, then I can see if we able to supports at the edge of the mat or standing table or something that's a little more alerting are they more responsive, is it easier to engage them or overwhelmed and you're getting less responses, so you can kind of figure out which combination works best. So, I usually try to do a little of both. I like to co-treat. It helps the team get all on the same page and seeing the same things and you get see how everybody do the more stimulating orienting in a different position and then also if I'm working with him alone. It's quieter. I can go slower. There's less distractions and that can sometimes help as well. So, then we're looking for are they able to interact with their environment. Are they starting to follow commands and we're looking to see if we can get them emerged, if we're noticing examples now they're in a minimally conscious state or they're now emerged from coma. And it's obviously, it's easier if you have a couple people that spot it, but people are we believe and trust each other. So, if I see something that's fine and then they can also try to do it or try to communicate. Like hey I think I got a, I think they moved their right foot when I asked them to or tracked me when I moved around the room or. I've been able to demonstrate that people have emerged from minimally conscious state by playing tic tac toe with them. So, then again, its automatic like functional, but they have to grab a pen, I have a white board, they are holding a pen and then I draw lines and if they make an X or and O and then I hand them a washcloth and I wipe it off. They're functionally using two objects. They're using a pen correctly

and they're using a washcloth and that works fine, demonstrating they're not in a coma anymore.

I: And they're aware?

R: Yep.

I: Very cool. I want to go back to the DOCs and the JFK that you're using. Can you just explain to me a little bit how you use those as part of your treatment? Do you use those diagnostically, prognostically to change your treatment plans? How does that inform you?

R: So, we use it to start with to try and get some sense of baseline of where they're at, so diagnostically. Personally, well no we would use it for prognosis too depending on what you're seeing that's happening with their responses when you're doing the different tests. So, we do the JFK once a week each discipline does it once a week and then the DOCs is also once a week. Sometimes, it's once every other week, but we tend to like to do it once week even if we don't need to do it, just to help keep track of how things are going. And that might be PT and I, with the DOCs we tend to co-treat? so we've got two people and one person that could be writing things down and two sets of eyes following they're responses.

I: Do you ever find discrepancies among the disciplines?

R: Sure, yeah which is why it is really helpful to co-treat. So, then we can talk about responses and if you are coming up with a few different score or interpretation, we can go back and look at the directions and make sure we're doing it right and we can talk about our differences and come up with a just to figure it out. I tend to be a little on the optimistic side and so I might see

things faster than other team members and they may not notice something that I’m noticing. So, it helps to communicate and talk it through and double check.

I: So, you said all three disciplines will administer the JFK?

R: Yes.

I: And then the DOCs is usually a co-treat?

R: And usually PT is involved in that one. PT and Speech; PT and OT.

I: Okay.

R: As far as back to your questions earlier where you’re like talk me through. So, we kind of talk through the initial as you see them responses. Then you can start doing some more things so, I’m looking obviously at both communication and at swallowing and once they seem to be able to respond tastes to whether that’s a little bit of a sucker, an ice chip, or something like that. Then I’m looking at signs of are they managing their secretions okay, are they not coughing, and then I might branch to applesauce or pudding again at some point decide that we can do 23:08??? Swallow study and progress from there. A lot of these patients come in with a trach and then so we are working on checking if they can tolerate 23:23?? Or capping. So, we’re pretty involved assessing that trialing edge and then progressing so if they do okay with a passing revalve for a minute then we get other, train other disciplines to have them start doing it as well. For how long, same with capping, so I often one of the people that helps initiate that and then talk to the doctors and get orders and get more people involved to be able to do that as well.

I: Can you described the passing revalve a little bit?

R: It’s a cap that goes, it’s a tube that goes in the top of the trach and it allows the person to breath in through their trach, but when they go to breath out the valve closes and they end up breathing out their mouth and that would allow them to vocalize or talk perhaps if they can do that. And also, since you have air passing through the vocal folds it improves their sensation and it’s a little easier for them to tell that they need to swallow or they might need to cough and when they do cough, it improves pressures so that they’re stronger and able to help clear their airway, so we’re trying to move pretty quick on either the passing revalve or plugging the trach. If they can, they 24:54??? status can tolerate it.

I: How do you know when somebody is ready for that?

R: How often they’re being suctioned? How much oxygen they’re on? How congested they sound? And then we can try out figure occlusions or we can wash our hands every wearing a glove?? And check and make sure that the cuff around the trach, if they have a cuff that that’s deflated. So, I usually talk to the doctors first because we try to, I talk to doctors and respiratory therapy and when we feel like we can give them that try and make sure the cuff’s deflated and I try…blood pressure and respiratory rate and oxygen saturation and if they seem like they’re tolerating it okay I would let the team know and kind of talk about what we want to try next.

I: What would be the difference then between the regular cap and passimier?

R: So the passimier valve is one-way valve so you can still breathe in through the trach and a cap, you’d have to breathe in and out your mouth tends to be just a little bit more work, so, if they’re not ready to breathe in and out their mouth yet, the passimier valve is a kind of a nice

step that’s a little bit less work. We also have the Shacony valve, you can adjust, it’s a little bit less resistance. It’s a little easier to be able to, it doesn’t need as much pressure. We’ve got these different levels of difficulty. It’s magic when it works.

I: So, I want to go back to, you talked about trying the tic tac toe and if people are able to use the pen or to use the washcloth. Name some other examples of things similar to that that you’ve tried with patients and some different responses that you’ve gotten or lack of responses.

R: Here’s where family is usually helpful they’re spending a lot of time with them and they’re trying to interact with them so they can help give your ideas. Jokes on the internet. I know. I’ve usually not initiated it, but I’ve heard from others and I’ve double checked to see if I can get the response as well, but if you’re new to something funny and the person smiles or laughs then you know they’re responding or reacting to what’s going on. One of the tastes and eating is another I think, I tell people it’s pretty hard to chew and swallow when you’re in coma so, it’s kind of sign that you’re aware of stuff and if you respond differently to a straw or a cup or a spoon and you close your lips around it and you chew things, chew ice chips and so, it’s another. Family and kids, we’ve had someone that had (28:34???) had a year old or ?? and she would come visit her day every day or his wife would come every day and so we called it the therapy baby. We’d use her. We have her crawl on him and sit next to him and hand him a ball and he was a lot more alert and responsive. Some of the time when she was around he’d get some responses from him more with her assistance.

I: Why do you think that was?

R: Cause she's cute and his daughter. So, I'm a stranger. So, she's got the familiarity factor. We would move her around the room and have her talk to him. Yeah, it was fun.

I: So, I know you've talked a lot about having family involved and that seems like it's something that's really key to a lot of these…

R: (29:37??) spending all that time with people.

I: If you've ever had a situation where a family, family has said, "well I'm seeing this," and you

R: You can't elicit it. Then I can just be honest with that. Now, I can do the show me or can we work together, can we see what he can do, what you can elicit and early, there's one case where when we tried co-training they weren't able, they were never able to get him to do that when we were around. We try to be supportive about…that doesn't mean that it wasn't happening, but it's likely overinterpreting what was going on and so by working together and watching what we were doing kind of talking through what we were looking for. That's the tough part is you trying to keep hope, but keep it realistic and I don't want to crush them, but I also was not seeing what they were seeing so I felt bad in some ways in that, you wanted him to get better and it would've been nice he been able to do what they were saying he could do. You know that it doesn't mean that it wasn't happening, it just means that I never saw it and they could never get him to do or say something…it'd been years I'm not positive like say a word and he just never got anything close.

I: I want to go back to the concept of overinterpreting and how do you explain to families a reflexive versus a purposeful response?

R: Or so more generalized, so we talk about how your body can just respond because something is happened and what we're looking for is something specific to what the particular stimulus is or what you're doing to them. So, another advantage of co-treating or having someone work with you is you can have them do something and then you can talk about what you see and what did you notice, what did they do. And how specific is that to what you did and so, it's great that they responded, I mean that's cool right there. And then here's what we're so, more specific direct response would be what we're looking for so, if they blink when you call their name, that's cool, that they did something and you see that they can do it again, but then can they, can you get them to actually look at you when you call their name. That's something that's more specific to what you're doing. So, describing what you're seeing and either what, if they're watching us and what we're doing and that we can talk through what we're seeing or if we have them try something, then we can talk about what we're seeing together. So, labeling what's going on can help clear that up and make that make more sense. It gets really tough when families can't be here as much and if there, they've got other kids they have to take care of or if they're working full-time. So, you know if they're coming in on the evenings. So, one of our patients, one was injured in the same car accident she was so her mom wasn't available. Her uncle was trying to help out, but he was working full-time. So, we set-up a kind of diary, we would each write things to her and that way communicate with everybody what was going on. Hey, I stopped by to see you and (34:02??) and you smiled or whatever. And then that way everyone would kind of see and then we also knew other things we could try. We all could try different things. And then we would also touch base. Call and talk to the on call. That kind of

stuff, but it’s harder to do. You don’t go as detailed and as much in depth with the education if they’re not there. It’s harder.

I: Anything else about any of the patients that we have already touched on about working with this population that I haven’t asked you that you want to share?

R: So, challenges are when they don’t get better or they’re getting worse and and when they stay really long, so, it helps to have a (35:09??) program and I tend to kind of fight to keep counting it down and not extend it long…I just find that really exhausting as a provider, but if you have someone here (35:28??) and they haven’t gotten better ??? you’re not helping them. That’s a challenge. So, very long stays with people not improving is really tiring and a lot of work. And (35:55??) are often resistant to make (35:58??) why I think that heads up pretty concrete now it’s 11 weeks, now it’s 10 weeks and at some point let’s start talking about where to next with the knowledge that and continuing the same, if they happen to emerge…we get to a point where we can do more to help them and we we’re on it, but yeah, so that’s tough. Discharging having to move on when you’re not helping. It’s not working. I think that’s it.

I: Okay, well we are all set. Thank you so much.

R: You’re welcome.

Clinical Interview 19

I: Interviewer

R: Responder

I: …do we have your consent to continue?

R: Yes you do!

I: Okay, so I want to start with your description of your role with these patients in a disordered state of consciousness and what kinds of things that you would typically do with them.

R: Okay, so being a recreation therapist typically the brain injury unit we would work with individuals helping them resume their interested leisure activities so for somebody who's in a state of emerging consciousness, I feel like we work quite a bit with the families that's kind of our primary concern is to help them adjust and kind of navigate what is available on the unit in terms of resources and helping the family to understand what our role would be. So, we don't typically get or we aren't as involved with our emerging consciousness patients as we are compared to those who have had strokes or brain injuries that are at a higher level of consciousness or working in a mildish state of injury where they're able to respond to physical commands and things like that. So, we typically don't see them as often. We would see them maybe three days a week maybe for a half hour session at bedside, a lot of our sessions are at bedside versus in the clinic or off the unit as depending on the person's doing we can actually maybe take the veterans in their chair outdoors with the family coming along with them and that's a really big deal because a lot of times most of what's going on is in the room, or on the unit, or just off the unit so being able to get outdoors that's a really huge component. Working with the individuals themselves a lot of times we're doing very basic minimal things, we're looking for their response to some sort of stimulus whether it's them being able to respond verbally or maybe its looking at them being able to track something, an object of some kind, sometimes its responding to or figuring out how to respond to whether or not their looking at a particular objects, so we might hold two objects up for them to look at and say, "Can you look at the…?" Whatever. Those objects a lot of times are objects that would be familiar to them so in the area or arena of sports and recreation many times we would be holding two completely different objects so it might be a fishing pole versus a golf ball and holding them on very different planes, so that they would either have to turn their head and look at one or the other, so that we could identify whether or not they were really looking at the objects. So, those are some of the things we do sometimes just responding to tactile stimuli where we might touch with a certain object. It might be kind of a massaging tool, it might be us using bubbles, you know in the room and having them track something or reach up to break a bubble, but a lot of the equipment, things that we use that are sensory oriented or even visually stimulating for them are in a lot of times as we can in the arena of sports and recreation or things that would be familiar to using themselves and I think that's what kind of separates us from of the other disciplines. They might be using balls, but they might be just simple test balls or something like that whereas we're using different types of equipment with them that might be something that they would be using or doing, so it might be a picture of a kayak or it might be, if they enjoy golfing. It might be a golf club and so that's something that the family doesn't see other therapies using typically and we work a lot, a lot of times we do co-treats with other disciplines too or we help

kind of understand so if PTs are working on different physical modalities we can use those and incorporate those into our sessions by using some of those sports balls or whatever so that's kind of fun.

I: So, I want you to try and think about a specific patient who was emerging consciousness or at that lower end and tell me about a time when you were surprised.

R: So, I can think of one individual where I was working with him. He was non-verbal at least up until that point in his rehabilitation. His parents were both in the room and this was actually within the last six months, but his parents were in the room and he hadn't spoken yet and based on all of the pictures I was seeing on the wall and what I had learned of him of his leisure interests, he really enjoyed water and so he was in the coast guard. He was very much into water sports, kayaking, swimming, surfing, wakeboarding you name it. So, I was asking him to think about a memory of travel. Something that he was doing that was really important to him in terms of travel kind of like a memorable situation and I brought a map in and I said can you tell me anything, tell me anything, I was trying to get him to verbalize and I said tell me anything about the, it was a map of the united states, one of the states that you've been to and something that you really like and he wasn't able to express himself verbally, however, I could see his eyes kind of going to the left side of the map. I said are we talking about the West Coast here or ? Well, he was looking at Hawaii and his mom and dad knew that he was looking at Hawaii and that's where he was stationed so they had said, "I bet you you're looking at Hawaii, huh?" And I said oh Hawaii oh my gosh, I think I might be going to Hawaii at the end of the year, can you tell me what's the best island? I would love to take my family there. Which island would you go to? And he said Maui. And his family was just…they started crying of course and I was like tearing up. So, it was all the disciplines combined and him getting that point in his rehab where he was crossing that threshold of being able to verbalize, but unfortunately hadn't said anything at least to his parents that come up and any of the therapists that come up to that point, so it was really exciting for them to be there and for him to say that when they were there and he just kind of started building from there and then his speech became…his expressive patterns became a little bit more often in terms of him being able to vocalize. It wasn't every single time that I would be there for from that session on, but certainly more often that he was expressing himself verbally so that was really a surprise and that's typically something I feel like there are a lot of surprises that happen in recreation therapy during our sessions. We have animal-assisted therapy dogs that come in and work with our veterans and that's something that we tend to see a little bit more reaction to. We'll place the dog, for example, next to the bed or sometimes we'll even have the veteran moved into our clinic which is just right on the unit and we can bring the volunteers and their dog in. We can actually have the dog sit next to them on chair so that they could be able to visualize them better or even reach out and pet them, but many, many times we've had somebody using their stronger dominant hand to be able to either open up their fingers to just feel the dog or we'll watch very very intently for movement in any of the fingers and when we can get a purposeful response in that or them turning over towards the dog, that's really exciting to see. A lot of times we see more expression that way too, in terms of their facial expressions a little bit broader affect we can see. So, that's kind of cool. Using those types of programs that we have to elicit some type of response.

I: Can you either elaborate or think of a specific person and talk to me a little bit more about what you see in those facial response or those reactions to the dog, you talked about head turning and…

R: Sure, yeah so and we use a lot of different modalities. So, it might be that we're positioning the dog in a room somewhere or we might have them grip some type of object. It might be a brush and we might ask them to brush the dog. And sometimes they're, those tasks are more exaggerated and sometimes we really have to give a hand over hand sort of assistance to help make that happen sometimes it's just holding a piece of food in their hand and then us saying okay we want you to feed the dogs, go ahead and open up your fingers and then someone might be able to do that. It might be for somebody who's working on verbalizing it might be that they state some kind of command like sit and a lot of times it's not very loud when they're in those very stages, but it might be loud enough for them to be able to get the dog to follow that command sit or stand or stay or okay, some of those more simple words or one word responses. We've used music also to help sort of elicit responses. I can think of one patient who had been down to a music concert at and just wasn't, just really not following commands and not in that state where we were at that next level where we were seeing anything. We were, the music was kind of loud and I was a little nervous about it being kind of loud for the veterans, but it was held in the auditorium and I noticed the veteran actually tapping his foot and so it was the foot that, that was his good strong side, but I was just like, I was like oh man we haven't seen that yet, so I guess the loud music really works. So, sometimes it's those things that they wouldn't typically be doing on a day-to-day basis even if we were playing music in the room and it was a strong beat or something, we might not see that reaction, but I know that music and the dogs and then objects that are familiar to them. Those are a lot of times, that kind of trail of things we use, we can get a little more substance.

I: That's very cool. That's not the typical stuff you see.

R: No, in your regular PT or OT or other sessions and that's what I think is kind of exciting and sort of separates us even going outside. I know for one of my veterans that was in emerging state. It was cold and that's kind of why we took him out because we were thinking he doesn't like the cold. Sometimes, it's that adverse reaction we're looking for. Like somebody who really does not like rap music and we play rap music and their eyes get really wide or something and they're just like "turn this off" or saying inside, just tell their reaction is like "I don't like it." But when we took this particular veteran outside, we bundled him up and got him outside. It was literally we got him out the front revolving doors and was like how do you like this? And he goes "in." And I was like right we're doing it. Let's go. So, yeah that was pretty solid. It was like now we know. And you know during the previous 30 minutes of that session we weren't getting much out of him. So, it was sort of like yeah, the cold on my face, I don't like it.

I: That's an interesting concept because I think people are typically like what is this person like and what can we tap into in that, it's the opposite, but it works.

R: Sometimes, it's the adverse reaction that really gets them to respond yeah and of course we never put them in situations where if they never liked dogs or were afraid of dogs, but you know it's those types of things that hot or the really, cold. The sensitivities to different things we've got I know that a lot of times we get a lot of information from families and that's why they're so critical in that state. That early state because we can't ask the veteran what are your favorite things. We have a form that's called All About Me that we ask the family to help, to fill out. We can get some information from the records. If they were at a previous hospital, but it's really, great to sit down we the veteran's family or even significant other. Somebody who provides for the overview of what the interests are and then we can kind of go from there and go okay. This, this and this or she likes this, this, and this and then we can kind of expand upon those things and sometimes we really got to get creative because sometimes people are

just not into a lot of the things we're providing. They might be into skydiving and sort of this high risk sports and oh man, you know, we're not gonna take you out on the motorcycle and we're not gonna do this, but we have, we can create pictures or we can create or do certain creative things to kind of help try and get a response from them. So, yeah that's kind of…

I: Yeah, very cool. So, let's switch gears a little and I want you to tell me a frustrating experience that you had with one of these patients.

R: Sure. So, yeah I think it's hard sometimes when you go in to see a patient and you fell like you've kind of tried everything you can and then you're just not you know getting them to respond. It might be that. I think for me a lot of times is. We try as a team to sort of schedule appointments so that the veteran has downtime in between those apartments to avoid overstimulation. And that something that we really have to work collectively as a team to do and with the family to in providing education to them that we're not doing back to back sessions. We can send over and email and say okay lets split these up so that we've got some downtime in beween. A lot of times because rec therapy is a discipline that provides programming in the evenings and on weekends, a lot of times spacing those appointments out means that we would see them in the evening or the late afternoon and sometimes by that time after having three separate therapies they are just wiped out. So, it's like I think that that's just something that comes to mind that I think it feels like I strike out a lot, like when we schedule somebody at the same time or in that block where we've got some downtime before and after you a lot of times if there in that emerging state whether it's a 3 or 4 o'clock appointment or even 5 o'clock or something you now we go on to see them and they're just out you know and the nurse will say oh man we've just laid him down he's been up all day…it's like aww, and then you just know that you're probably not going to get much out of him so, that's kind of a challenging thing you know is, it's challenging and it's not. When they get to the point that they are tolerating a little bit more therapy and maybe can have a little bit more peak alert times then being able to provide programming and therapy in the evening can be a benefit too. It's just that there is that sort of gap that you know you kind of okay there, this is going to work or it's not going to work and so. Yeah, and I've had to a lot of times reschedule my appointments like in the afternoon or whatever just to which is another luxury if I'm working a late shift for a workshift??(18:17) 8:30 pm and then I could even see them at 6 or 7 and that sometimes is when they are more alert as was the case with the gentleman we brought down to the music program. His, all of our evening programs at the VA are held at 7 o'clock and so sometimes that can be a good time because by that time they've had a chance finish all their therapies, get a good nap, get dinner in, and now they're ready for something. So, that's kind of nice thing too. Or it's kind of nice to be able to take the veterans on like a stroll around the building or something in the evening when the sun's not so hot and family can go with and that's kind of nice time to be able to spend with family, provide education, things like that too so.

I: So, what I'm wondering then if you run into this situation a lot where you know the team is saying this person is doing this, and this, and this and they're really interested and you go in and you're like I'm not seeing this.

R: Right! Yes, that happens, that happens. I think at that point where we've tried those things that we're talking about in our interdisciplinary rounds meetings and saying hey, you know, and everybody is saying I'm seeing this and aww man not getting it. A lot of times, then what I do is maybe connect with one of the other therapists and say you know can I see what you're seeing. I would love to be able to see

what you do to sort of help elicit that particular response. And to be honest with you, a lot of times that happens with the other therapists asking us as rec therapists, like oh man, like I'm not seeing that range of affect or I'm not seeing that like him turn up his lips into a smile. I'm not seeing him respond in that way whatever. And sometimes (20:00)

Clinical Interview 20

I: Interviewer

R: Responder

I: Is it okay for me to start asking you questions?

R: Yes, it is.

I: Okay, so the first thing I want you to do is to think of about a patient that when you walked into the room for the first time you're encounter surprised you. So, what you were expecting to see maybe wasn't what you saw.

R: Sure, and you know only in reference to patients in disordered--

I: So, a patient that has--yeah so can…

R: You mean like the emerging consciousness--

I: Exactly, so when you tell me your stories about these patients they may have come in in a vegetative or a minimally conscious state and then gone on to emerge, but yes so that first encounter was of a person that was in a vegetative or a minimally conscious state.

R: Okay, alright. I thought of someone.

I: Okay, so go ahead and tell me about what about that experience. So, what were you expecting and then what did you see?

R: I guess I was expecting something similar to what I saw. So, I'm trying to think of someone I was really surprised by. I think in most cases I knew the person was in this state and so I sort of prepared myself for a full range of non-responsiveness or just minimally responsive. Yeah, so I don't know--

I: Okay, so you don't have anything, you don't have any initial encounters that were--

R: So surprising.

I: Okay, so you never had a situation where information that was passed on to you prior to the admission or anything it sounded like a person was functioning at a certain level and then when you walked in to meet them they were completely different? Can you think of any situations like that? Or maybe not completely different, but you had an idea in your mind and it wasn't maybe what you thought it would be?

R: You know, not really and I think that some of that might be that I'm often coming in a little later, so I hear from the therapist what they're seeing so.

I: So, you're not one of the first people in the room?

R: Yes.

I: So, then if that's the case then instead of thinking about that initial encounter, I want you to think about a patient that came in disordered state of consciousness and then for their duration of the stay maybe you were surprised by something that happened.

R: Yeah, I don't know if it's surprised by it as much as challenging or sad and tragic or uncomfortable. Like perhaps with some like yelling out and sort of those types of behaviors.

I: So, how about instead of thinking of a situation that surprised you. Can you---I know and we're going to go through a few different descriptors like that, but instead of thinking about surprise, maybe a patient that you had a certain expectation of maybe how they would do and then they did differently. Whether that was they responded less than you thought they would maybe upon initial or your first few contacts where maybe they responded more than you…that they had a better outcome than you were expecting. Can you think of an example like that?

R: Sure. I think some of surprises how well they did when they emerged and progressed.

I: Can you think of a specific example like that and describe it to me?

R: Some of these are like a while ago. So, the person just really starting to respond and then just slowly, real gradual, but if we sort of stayed at it they were able to stay long enough that they started to be able to respond and answer and engage in therapies and just slowly make gains.

I: Can you think of some…so thinking that patient, can you think of some specific examples of maybe things that they were responding to initially or early on in their stay and how those responses changed?

R: So, early on. I feel like I can't think of examples.

I: No, no it's kind of hard sometimes to think of examples I understand that, but if you can--

R: Just sort of not responding much or just localized or just responding to moving their head by somebody coming into the room. That level and then that sort of later getting to the some of the more [rancho four?] where it's just much more challenging. Challenging for the family members. So, like mostly I work with the families in those situations and then having some

behaviors that sometimes the family just has very mixed feelings about or is happy that they're sort of responding more, but then when it's more disinhibited feeling worried and…just had a flash of somebody else that might be a good example. Just getting upset that we're going to be angry at the patient or that the patient isn't cooperative or cooperating and sort of giving it a lot, the family giving it a lot of personal attribution and not understanding that these things sort of aren't under the control of the patient yet.

I: Okay, so that's a nice example. So, I like how you talked about how the patient started out maybe turning their head to respond to someone coming into the room and then they're moving on to a rancho four? level. So, give me a description of a patient who started out in, you know, localizing and doing different things like that and they've moved into a rancho four…can you give me an example of a behavior that that patient was exhibiting that you needed to try to help the family through or…

R: I can think of a few. They're sort of different patient, but the ones that come up the most that I need to help the family with is either the disinhibited crying out or yelling or sort of throwing things or something like that or having their hands in a diaper or something and that you know could be very distressing. The patient and the mom and so sometimes that's just really hard. And now the one that I'm thinking of that as he started to respond more, he would I think it was two patients early year on like in the war who were both injured in combat and then they were…it was in the confusion state so they would…one of them would really get into protection mode and like to help him in the shower or anything like that he would…I saw it more as the PTSD or just the trauma responses coming out, but he could not regulate them and did not always necessarily match with the environment. So aggressive. Striking out, but also

would probably it was that sense of threat because the environment didn't make sense. He was responding to that sense of threat much more of relation/proximity to his combat experiences I think.

I: And that was his protection mode you're saying? The aggressiveness?

R: Yeah, yeah and the striking out at therapists not letting them…yelling and striking out. Then I think of another patient as he emerged and it was him and his mom and she was so happy when he started talking. Then he got to the phase where he just was like randomly talking about combat experiences. And that was just really hard for the family and he couldn't regulate and what was difficult was it was more distressing for family and staff, but the patient wasn't distressed himself. And so, they wanted us to, they want the psychologist to sort of make him stop or mom wanted us to sort of do PTSD treatment. I had to help her understand that this was more distressing for us than for him because he really wasn't exhibiting--distress.

I: He was talking about it as if he---

R: Right, it would just be random.

I: Yeah, that's challenging, that's hard. Okay, so tell me about. So, you mention when they emerged. And we talk about emerging from consciousness a lot. So, can you kind of tell me in your own words what…like how do you know when you're working with a patient that has emerged from consciousness.

R: Well, again I think I have the benefit of the rest of the team. Truthfully, I think I don't trust that I know I mean that I may see some more responsive behaviors, but I have this one sample

so I think I know based on I think I improvement and it seems like something's happening, but I want to see what someone who's seeing him every day is seeing and so is this just random or is this going to be consistent. So, I think I've always relied on others doing the DOCS to really try to test and communicate that consistency because often a family member, somebody will see something and feel that they should have emerged. It's not consistent.

I: That was a really great description, but I'm going to take you a little further so without going into the definition…can you describe, so you're looking for consistency, but describe some of those behaviors that you first start to see that you think are indicative of that.

R: Consistently, responding to stimuli whether it's to touch and like sort of communicating with touch or following some basic level of command to either move their eyes this way or that way or to respond to whether something's hot, cold, red. So, basic level of stimuli, but it's just more consistently being able to follow some very simple commands.

I: So, I want you to think about a time that you were working with a patient in this state that you felt stuck with? And maybe not stuck with as in you're stuck with them as a patient, but felt stuck.

R: It's interesting because when we first started talking I felt like oh, I can't remember and now as we're talking these random patients are popping into my memory. So, okay I just thought of somebody.

I: Okay, so tell me about that.

R: And again, my situation is a little different because I'm one step removed from the patient so, I'm working with the family in those situations. It's maybe me working with the family that feels.

I: So, describe that and that's a good description. So, describe that to me then. A specific example of that.

R: So, a patient that was not, I have to think if he even technically emerged, essentially stayed in that minimally conscious state. So, was not progressing and the family became quite mistrusting of the team and angry with the team because they're son and they're brother were not progressing. And then I started to deal with staff feeling stuck with family.

I: And then feeling stuck with the patient as well I'm assuming?

R: Yeah, yeah.

I: So, tell me how you navigated that situation. Like how did you describe those things to the family to help them understand.

R: I'm thinking of multiple things as once. The whole research part of this.

I: You're doing great, just describe it.

R: I'm thinking it's easier for me to describe in general how because how I do it with one…I mean in general in terms of describing it, I would talk about the brain and talking about that depending on the level of understanding from the family, talking about the brain sort waking up, and the brain healing and that things need to sort of repair and heal and connect and swelling needs to go down and depending on what's going on how that's relating to often the

medical piece either storming or whatever. And of this one particular family that I had just happen to think about they had different cultural beliefs, so in that regard. I was trying to use language that would be consistent enough with their cultural beliefs in terms of spirits or things like that. And then it varied based on the family member. The daughter was much more Americanized and I could give the more medical and then with the parents and other relatives mostly what I would do is get their perception then try to use language consistent with theirs that could…I could use.

I: Okay, so thinking about that, so can you think of maybe a response that the patient was having that they perceived differently than maybe the team did or something like that and how you tried to explain that in language like a misperception they were having maybe and how you tried to describe it in language that they could understand?

R: Again, this is sort of a specific. They had different, a different conceptualization so they would really see things as spirits and stuff. So, that either the spirits were punishing or trying to take his soul, so, if he was distressed or uncomfortable or his blood pressure went up as he became agitated they would give that a lot of meaning and so I would try to…that one I would sort of have to gradually meet them sort of in the middle about.

I: Right, so how would you, that's very interesting, so how would you--

R: That's a very specific example--

I: No, but how

R: It doesn't generalize--

I: It's okay if it doesn't generalize, but it is a great example. So, can you give me an example of how if they were looking at him agitated, restless, interpreting that as a spiritual thing…can you give me an example of how you tried to steer them to understand the medical side of it? Like we said meeting them in the middle? So, how did you describe that to them?

R: I think I would use both sets of languages sort of. And say either that sometimes I would say this is the way medicine looks at things and medicine may not have all the answers or may not be able to understand all of what might be involved, but this is what we understand is happening and this is the way that we believe we can help and this is how we see this behavior now. There could be additional ways that spirits may be impacting it, but I think that's best for the medicine man or whoever to be dealing with that we can try to deal with this part. And then a lot of it was helping them to trust us that what we were doing was not trying to harm their son.

I: Can you think of an example of where they might of perceived something therapeutic that they perceived as harmful?

R: Sometimes it would seem to cause more agitation or distress, sometimes I think they understand the whole fear when a staff would rush and check his blood pressure, that it may have a urgency, but we were still caring. Another was we had to really learn some of the rituals of the culture and join with the family so that we could learn what some of these things meant. So, like mom didn't speak much English or very very little English, but would get upset if we did certain things. So, through dad or daughter we needed to learn how she was interpreting some of our behavior so this was the mung culture so there's a believe about going through doorways

that is at that point of going through the doorways that the spirits can take your soul. So, she was afraid as we whist him away to CT or an MRI that we were putting him at risk as he went through the doorway and so we needed to learn to cover, as long as you covered his head, he was protected so we needed to really learn that so that we could do the things that from a medical point of view were rather benign.

I: But it was helpful the mother?

R: Right, in terms of the distress and in terms of not more misunderstanding and this there was just a very strong baseline mistrust of our medical system, so none of that helped. So, working closely with the daughter and a whole lot of dynamics came up and they sort turned against the daughter and so, my role in this is helping the family deal with this, but also helping staff. Staff became very, very frustrated at times sort of why can't we just deal with the daughter, why can't we make the mom leave, why do we have to have these little bracelets on him? We wouldn't let other people do that. Then there was an instance where they wanted…we worked closely to try and incorporate some of their rituals, but one of the rituals was to have a knife under the bed, under the pillow to again ward off the spirits. That became an issue with security and we didn't know…so at one point the nurse just moved the pillow…we didn't know mom had put these…

I: That is was underneath there?

R: Right.

I: Oh no.

R: So, this, we really started working with them once we discovered, like we had something to work together about.

I: So, you're somewhat of a liaison or a conduit between the family and the staff to help.

R: That's where my role is and so I'm communicating to the staff about the process of adjustment and grief and loss that the family is experiencing and how that may be expressed. And so even when I think of staff like rehab therapists feeling stuck I think our team is really good at not directing toward the patient and not even necessarily feeling that for the patient. It becomes between staff and the family. And that that's where they end up feeling stuck. They know what they can do.

I: So, clinically they can make a plan and follow it up.

R: Right, as well as they can understand they can modulate their, they have a framework for what's going on, so I think they become less frustrated with the patient and are able to see the tiniest gains as something positive or be let's there's not gains so much, so they will now likely feel a sense of sadness understanding what this may mean and think that. So, there's that. I think that sometimes they, staff will become frustrated when they feel that, I don't know that I'm…I shouldn't be seeing the patient so often. He's not making any gains.

I: That was a great situation as well. I'm going to take you know to the other end of the spectrum. I want you to tell me about your most memorable experience working with a patient and their family that came in in a disordered state of consciousness. They didn't necessarily have to leave at that point, but they came in that way.

R: It was a long journey. (laughs) Yes, I think the patient was here, this again was early, was here probably close to a year. He was here a long time. He was here a very long time. And so, of that was…I mean he was making progress, making gains. So, I think more to the, the other end, the more…I mean I see him still as an outpatient. So, he's somebody who had a long journey. He had a very tough time here. Had a very tough time. He was one I'm thinking of in terms of having some of those reactions in the shower everywhere and his rancho four? stage was agitated, violent, aggressive, behavior and very a lot of difficulty in regulation who overtime a long, long, long time continued to progress lot of family dynamics stuff. And mom ultimately sort of, an aunt and somebody became guardian. And she was good help for him he ended up living with her, but I think it was his house. She ended up living there. Just a really, really long process. And he went to our P trip.

I: Okay, tell me what P trip is again?

R: Okay, so that's our residential post-acute community reintegration. So, he was here an inpatient for a while. I can't remember if he went…he was there so many months. 8-10 months maybe. I don't remember if he went directly there or went home, but ultimately went to our post-acute community reintegration program and he…one of the most memorable experiences was we had an open house for the program and he was a patient who test all of the hospital rules. This was way early and we didn't have the hospital was set up to deal with this kind of patient and so we had really pressed the chief of staff to allow some changes like to allow us to bring him to the smoke shack or different things. We would do things to help with his agitation. His behavior caused security response and arguments between different departments where we sort of had the chief of staff, it was educating them about TBI, that this wasn't mental

health and you know that you deal with a differently and coming in with a bunch of walkie, security walkie talkies was not gonna help his agitation. So, I just remember bringing in the chief of staff to see this patient in our P-trip program where he had his own room and he had it spotless and he had his bed made perfectly. He was talking. And the chief of staff only remembered very difficult behavioral--

I: So, this was after? He had seen this person when they were in an agitated state and now this was--

R: Probably, a year later that he's still on our continuum of care here. And I cause we knew he was gonna get there.

I: So, what made you know when he was back in that agitated state?

R: When he was, in the earlier agitation I knew that if we tried to handle it in a certain way we'd have greater success. I didn't how far we'd get, but I knew we needed to address it in a certain way and then. I think maybe I had a sense that this is a person who is so determined and such a fighter sort of that it wasn't always effective, but if we could channel that or harness that or embrace and shape it into a different direction that he, that that's the drive that's going to get him further than most as long as we could get it to be more effective. So, he's probably one of the ones that…yeah.

I: Those are good stories, right? Well, I want to be conscious of time because I know it's almost 9:00 is there anything else that I didn't ask you about or anything else that you'd like to tell me about working with this population?

R: I think as we were talking and different memories of different patients came up, I think one of the hardest parts is when they don't improve in working with the family. And so, I'm going to follow-up on one of the things that was the, I thought it was someone that was surprising when I came into the room. And it was less the patient than the mom. So, the patient was non-responsive, but like the mom was just in his face and yelling and sort of on the bed and. As he started to respond, she did that more and some of it was obviously her anxiety and her anxiety and fear of losing him, but it was also…just a version of how they related in that sort of your my marine and you never stop and you never---sort of like the drill sergeant in his face and you do this and like that was shocking to me. In the beginning, that she was just in his face and for a while. So, it was less the patient and staff had, understandably had a very hard time with that.

I: So, do you think that she eventually stopped doing some of the because of his improvement or because of education or?

R: I think both, but we did provide a lot of education and then I provided individual therapy for her and it was really about grieving. Her fears and as part of the individual therapy there's a whole lot of education, but being able to get to her emotional experience and processing about trying to make sense of this and trying to accept. And she was one who actually I think was very effective in her process and her adjustments and her acceptance. That she did come to understand and accept and switch her level, her framework. So, he went back home, but he still or maybe he went to a different facility closer, but yeah, I'm not sure which I think maybe he went home and then was in a facility and he ended up passing away from infection. And she was able to deal with that. She actually called me a year later and told me how he had just passed away and expressed her gratitude just by like in the beginning it was really hard. She

was not necessarily fond of the team. It's usually when someone gets stuck in their grieving and grief and loss that a lot of the difficulties comes out. And she was able to…so that's where I'm working, that's where the surprise was in the beginning where before I was this was newer to me and to have family members right there. We didn't used to. So, to see her response was the surprise more than the patient's.

I: That's a really--thank you for sharing that perspective. I think sometimes we forget the journey that the patient and the family are on and they're parallel.

R: Parallel, but at different timing and so yeah again that's my role so it's sort of different angle. I would obviously answer these questions from a slightly different point of view.

I: And that's helpful for us so thank you for sharing that perspective. We appreciate that. Is there anything else you want to tell me?

R: No, thank you for doing this.

Clinical Interview 21

I: Interviewer

R: Responder

I: Is it okay with you to participate in this interview?

R: Yes.

I: Okay, do you have any questions for me before we get started?

R: No.

I: No, okay. So, the first thing I want you to tell me about is I want you to think of an experience where you had an interaction with a patient that came in a disordered state of consciousness, so vegetative or minimally conscious and you were surprised by that initial interaction so, what you expected to see was maybe different from what you saw. Can you think of an example like that?

R: I had a specific patient that came in with both his, both sides of his skull were removed and he was unconscious. His body was, yeah, he was putting his arms in, everything was very stiff and very scary.

I: Okay and how was that different, what did you expect to see when you…

R: It was unexpected to see the way his body would turn out; his arms would turn almost completely around…

I: Like this?

R: Yes. It just, he was storming, neuro storming.

I: Okay, so describe that to me. I understand what you're saying, but

R: So, neuro storming is, his brain, everything was firing all at once. So, his body would turn in. The limbs would turn in, he was sweating, his pulse was 160. Trying to get his, just everything was happening all at once with this patient.

I: Can you think of a situation of surprise with either the same patient or a different patient throughout that duration of the stay then here that was surprising to you?

R: It's the same patient. He, we really thought that he was not going to pull through this and that if he did pull through it, he would not have a great quality of life. He progressed over the months and actually walked out of the unit.

I: Oh, that's awesome!

R: He walked out of the unit. He calls the unit now and he works a job. He's back to where he originally was which is incredible.

I: That's really cool.

R: Very, very fun to watch.

I: Okay, can you think of patient that you worked with that you felt stuck?

R: You mean? What do you mean stuck?

I: So, have you felt like maybe they were stuck and so you just things weren't?

R: Like, he was just not going anywhere?

I: Yeah.

R: Yeah, we had a young man that, it seemed like he was coming to and then there was like a plateau. Things just stopped moving forward.

I: Can you give me some specific examples when you say that it seemed like he was coming to, like what kinds of things was he doing?

R: He would respond, squeeze your hand. He would respond with once for yes, twice for no. You could see in his face that he was there. He understood you. He was hearing you. He was just not able to vocally respond almost like he just…he's present, his mind is present, but his body just didn't do what it was supposed or what he wanted it to do.

I: Okay, so when you say that, you could tell by looking at his face that he was there, can you describe that? Like, what about his face made you think that he was there?

R: His eyes were very telling. He would follow you. Again, the blink yes once, blink once for yes, twice for no. You could see the frustration. He had facial expressions, frustration in his face you could see. He would cry. It's almost like guessing, you're guessing with him, you know. Okay, do you feel sick, are you scared, are you sad to get that answer. So, you could see with his eyes, he'd smile or frown.

I: Okay, so he was starting to do those things? He was making progress and then he kind of just stopped?

R: Right.

I: So, how did you navigate that situation or what happened then when he just stopped doing more things, when he stopped progressing?

R: You just get to that point where you encourage him to continue with what he can do. The blinking and all his facial expressions and all of that. Coaching him through that. Try to make him as comfortable as possible and just follow along the expression.

I: So, he just kind of stop progressing and that's kind of where he stayed then?

R: Yeah, he just kind of, that was baseline, that was the new baseline.

I: Okay, alright. Then did he leave rehab at that point.

R: Yeah, he did leave. And he does come back because he has appointments here. His mom brings him back.

I: Oh, okay and has he changed at all since?

R: No.

I: That you notice?

R: No, unfortunately no.

I: So, that's kind of where he stopped.

R: That's baseline.

I: Okay, alright. Do you have any other examples of feeling stuck?

R: I think it happens a lot actually and there's different levels of stuck. It's hard because we don't always see them later on and we don't know what they were before. And we try to get as

much information from family and friends as we can. We have a patient that, we know that she was very tough, very strong, very strong-willed, but she's very young. She was 18 when she got here. So, she's just a baby. We watched her go from unconscious to the shunt going in and then the infections. So, this young lady has gone through every bad thing that could happen, has happened. And now, we're at kind of a baseline where mentally we're not sure, "Is this baseline?" "Is this normal?" because she's so young.

I: So, when you say baseline like baseline of where she was before she started having all these medical complications?

R: Before the trauma.

I: Oh, before the trauma.

R: Before the trauma. Now, she's just kind of, we can't tell if she's stuck or if this is the normal 19-year-old. Now, she's 19. Is this the normal 19-year-old marine kind of attitude? (laughs)

I: So, she's kind of in a agitated…

R: Not agitated. She's very sexual. She's very vocal. She's very loud. She has a dirty mouth. (laughs)

I: Okay, alright.

R: And that's kind of where we've stopped at this point. So, again we don't know if she's stuck…

I: Stuck in that place?

R: Yes.

I: Okay, okay. I'm going to have you move on to a different example now. I want you to think about the most memorable experience that you've had working with someone who came in to rehab in a vegetative or a minimally conscious state and describe that experience.

R: Let's see. He came in unconscious, actually it was a fight to get him here. They flew him in from xxx. Again, very young man. They couldn't even get him stable to get him here. So, when they finally did get him here he came in he was stable for a day or two and then it went downhill. He had to have surgery then a shunt placed. We went through, it seemed like all of the stages the way he…the confused, the scared, the angry, the sexual and slide back to angry. (laughs)

I: Okay, can you kind of take me through that? So, describe to me what he was like when he was waking up or before he woke up. Like what was he doing when he first came?

R: When he first came he was in that vegetative state. I'd say days into it. He kind of was conscious coming to, very scared you could see. The scared, the cries. Had a lot of pain at first, but he was pretty heavily medicated. About a week into it, he pulling at tubes and just unhappy. Not understanding so pulling at everything, wanting everything out.

I: Sure, so you said he was like in that confused state? Right?

R: Absolutely. Absolute confusion.

I: So, when you say that he was starting to "come to" tell me what was he doing that made you realize that he was "coming to?"

R: When you go into the room, he would open his eyes for you if you asked him. Can you open your eyes? Squeezing hands. And it’s a slow process, even though he’s looking at you and you kind of just know that you’re talking to him. For me, I always talk to my patients. I always tell them what I’m doing and why I’m doing it. So, the confusion, just kind of scared.

I: So, there’s a sense that you get that they’re there.

R: You do. Absolutely.

I: Can you describe that at all? Can you describe that sense? Like, what is it? Can you put any words to it?

R: You read so much of a person with their eyes.

I: So, what is it about their eyes? Can you think about that and describe what it is that what are they doing with their eyes that make you think they’re there?

R: Following direction. They’ll follow you. Look at me. Can you see me? Try to get head nods. Again, eyes and hands are important when they’re there. You can see the tears. You know when you get angry, you squint or your eyes get squinty or surprised, your eyes are fully opened.

I: So, you feel like you’re seeing reactions in their eyes to what’s going on in the room?

R: Yep.

I: Is that fair to say that?

R: Absolutely.

I: Okay, so tell me about…first of all…okay so we were talking about the most memorable experience. You were talking to me about how we went through all the stages. So, what was it about that experience that's the most memorable to you? Is it where he ended up? Or?

R: Where he ended up, yes, not completely recovered. He'll always have a deficit, but to see him go through this process and actually come back out of it. Having memories and knowing what his life was and all of those things. He doesn't remember a block of time. He doesn't remember waking up and using his eyes and talking and things like that, but he was here for a long time. So, watching the process of him "coming to" to him actually getting up and walking. Such a huge thing.

I: That's exciting, right? It's nice to see.

R: It's amazing! It's, I don't know you don't get a feeling like that just doing your regular job. It's pretty amazing.

I: It's pretty cool. Okay, so along the same lines can you think of the most rewarding experience that you've had working with a patient like this? Who started out in a vegetative or a minimally conscious state?

R: It goes back to the gentlemen who had both of his skull caps removed. Never in a million years would we have thought that he would completely recover. You would never know that he was in such a scary, terrible state. We couldn't get his heart rate under 160. We sat in the room with washcloths. We did everything possible to keep him comfortable. Watching him go through therapies. Standing up in the hallway. Me, other staff coaching him, "Come on, Marine. Get moving, Marine. Keep going." It's an incredible feeling. And for him to actually do it, to walk

to me and get there and the joy in his face. The joy in his wife and his mom. To see him come back.

I: So, describe to me one of the times that you were with him when he got into this standing frame. Can you describe what that was like and how he reacted to it? May be what his body was doing or what you know…?

R: He was with therapy. They'll start from one into the hallway, getting up out of the chair. He's got a put his hands on the chair. In the beginning, he's getting instructions "Make sure the breaks are on…on the wheelchair" "Put both hands on when you stand up." I'm standing at the other end of the hallway. He's standing and he's trying to stand tall because he's a Marine. You can see, it's hard to get up, but he gets up. He stands and catches his bearings. It's one step at a time. It's slow steps. It's hard for him, but he's doing it. He's pushing. Again, I'm at the end of the hallway yelling, "Come on, Marine. Let's do this." And for him to respond to that and keep going. He takes ten steps one day and then pushing for twenty the next. "Come on. You can do this." Again, the joy to see in his face when he actually gets there. It's our victory.

I: Sure, absolutely. That's a huge victory, right? So, then describe to me. Can you describe a little bit about before he got to the point where he was able to get in the standing chair? So, once you were able to kind of manage some of the storming and everything medically; describe to me kind of how he was responding to general stimulation in the room.

R: Low stimulation. We kept everything kind of dim. He had fantastic family that were here often. We actually had to shoo them away sometimes, but them to control, it was important to keep things quiet. You could see when things got loud and there was too much stimulation, his

heart rate would go back up. Not that he would neuro-storm then, but you could see the anxiety. You could see body stiffen out. Then you could see him relax once things were quiet. We used lots of essential oils in that room. Rubbed feet.

I: To relax him?

R: To keep him calm and relaxed.

I: So, when you were doing those kinds of things would you say that he was still in a vegetative or a minimally conscious state?

R: Yes.

I: So, when you were rubbing his feet…how would he respond to that?

R: You could see his body physically relax a little bit. There wasn't much of a facial response or anything like that, but you could see again, his body would relax.

I: So, then as he progressed did you continue to have keep a low stim environment?

R: Absolutely, for a long time it was quiet. They would come bedside to do therapies and stuff like that. Once he was more conscious, he was young, he wanted to see people and interact with people, but it took a little while. We kind of eased into the bigger groups and things like that. So, when he had may be recovered consciousness or was at a little bit higher level…would you still sometimes help him calm down by rubbing his feet again or did that…It became more when he was more conscious you could rub shoulders or just you could even, when he was more conscious, let's take some deep breaths, in through your nose…out through your mouth. Again, if he was too overwhelmed back to the room where it's quiet.

I: And how would he react? Can you describe his response to those types of things?

R: To too much stimulation?

I: Well, like let's say so you when from rubbing his feet to kind of relaxing and calming down to doing his shoulders and deep breaths…

R: You could feel the tension. It did take a lot of attention and time with him because it's…I mean his whole body would respond to the anxiety or the tension or too much noise. So, it was important with him to do everything you could to help him relax. You rub the shoulders. You even use the actual oils on him. And that was something that his wife did at home before his accident.

I: Oh, interesting.

R: So, that would…you could feel the tension kind of coming out with his shoulders. Explain to him, it's okay for you to rest. Sometimes because they're military, sometimes it's hard for them to understand that you have to rest. So, we would put him back to bed and kind of force the issue of even if you don't go to sleep, you're just going to lay here for a little while.

I: Yeah, none of us are, right?

R: Right.

I: None of us are good at that. (laughs) So, can you think of an experience that was frustrating working with a patient like this?

R: Yeah, we just had a patient, stuck I mean stuck in this mean, angry, sexual state.

I: And did they come in a vegetative or minimally conscious state?

R: He came in minimally conscious. He was on special right away, on a one-to-one.

I: Oh, okay because of what? Why was he on one-to-one right away?

R: As he was emerging, he emerged right into angry, you know pulling things, yelling. We had him in a posey bed.

I: So, describe that. I know what a posey bed is, but will you describe it?

R: A bed, it's almost got a tent over it. Where it zips. You can open it and it unzips on both sides, so there's access to the bed on both sides. It's like a tent, it zips, but it zips from the outside and you can clip it shut.

I: And the purpose of using that type of bed is…

R: Safety. Safety for him, safety for us. He tend to calm down. In the beginning, he would calm down immensely. I think it made him feel safe. Actually, I had some difference of opinions on the bed for a little while. It's hard because you feel like, you are restraining your veteran in a very strange way. It feels mean almost, but he was so combative as he "came to" and he was 6'4" and 280 lbs. I'm 5'3." So, it very intimidating.

I: It's a little overwhelming.

R: It is. So, it's overwhelming, it's scary and all you can do is redirect. Try to redirect the conversation. The entire time he was here was convinced that we were getting married. So, it was very difficult. It was very difficult to be in a room and when you're on a one-to-one you're in the room for 8 hours. So, 8 hours of that is very relentless, very difficult.

I: Yeah, I can see where that would be very frustrating. Can you think of another situation that was maybe frustrating? Were there ever any patients that were in the vegetative minimally conscious state that you found for whatever reason to be a frustrating experience?

R: I can think of a specific one. I mean there's ten specific ones. What's frustrating is when you know how wonderful they are. You can see the pictures and things like that. He's somebody that, he does respond. He can't open his eyes. He's not clear. It's very frustrating when you can't fix that. You can't seem to hit that one little spot that's going to make it all happen.

I: So, when you say he's not clear what do you mean by that.

R: Some words come out clearer than others, very garbled.

I: So, it's almost like he's trying to communicate.

R: He's trying. So, it's frustrating for him because we're not getting him. Frustrating because I'm not getting it and I want to get it because he's so wonderful. He's a very kind man. He'll all of sudden say, "Good morning." Or…he cares. So, it's frustrating.

I: So, he's kind and he cares, you can tell that by the way he's trying to interact now and you also know that by the way…

R: Family…

I: The family description of him before.

R: And pictures. Yeah.

I: Yeah, I know.

R: It's frustrating because you see that family too. That they want their loved one back.

I: Sure. Yeah, yeah that's hard.

R: That is the hardest part of our job, I think. Is not being able to pull that [inaudible 4:49] little bit back.

I: That things aren't necessarily going to every be back to the way they were. I think that's the hardest part about working with neurological disease.

R: Absolutely.

I: Okay, those are great, great examples. Are there any other examples that you can think of that either, from a surprised, stuck, rewarding…

R: Again, we have a lot of young people that have come through. My very first patient was a man that had just gotten married and he came in in a vegetative state. He left here kind of conscious, not…

I: Okay, describe kind of conscious. Like what, what was he doing?

R: He would tell you no he doesn't want anything to eat. He would use his hands to…some people though he was trying to hit or things like that, but it was more of a no I don't want this. Could shake his head yes or no. It was again stuck with no vocal response really, but still had life. He had his wife was here daily. Successful because we did get him to wake up. He's aware of what's going on around him. He knows who his family is. And he responds to them, hugs and kisses. Will laugh, can cry, but just that's it.

I: So, he kind of got stuck right there? He didn't regain that…

R: Couldn't get up and walk. There was no walking. There was really no talking.

I: Was he eating?

R: He did eat. Yep. Yep. We made a deal in the very beginning when he started to really be conscious and remember things that when he would say well forget that. Get solid foods and stuff, we would have pizza. And he did, we had pizza and he was on top of the world. So, that was good.

I: That's cool. So, is there anything else, are there any other questions that you wish I would've asked you about working with this population? Or anything else you want to tell me about working with the vegetative/minimally conscious patients?

R: I think it's very hard to watch the process sometimes because they're all so different. You never really know. They don't ever come out of a vegetative state the same. Always, different. And it's time. Taking time. Sometimes, where we get lost because we are a hospital and it's a fast paced. I mean it could take ten minutes, but then it's with that patient just to show that patient that you have time that you want to hear or you want to figure it out because again sometimes it's blink with your eyes or squeeze with your hands or shake your head. You have to take time to figure that out and get to know your patient. And that helps when they know you are taking time. And they do know.

I: Oh, sure. That's the connection that you get with those patients, right?

R: There's always boundaries in the hospital and those boundaries are important yes, but when you work in a place like XXX and your patients are here long-term they count on you to get to

know them. We are the people that make them feel safe and that's important. The consistency of our staff. They get to know us and we get to know them. So, it is a social thing. It is being social with them and interacting with them. I think it helps. If you watch patients that even that don't have family to patients that do have family. The recovery is very different.

I: Yeah, that's a good point.

R: Their interactions, their consistency, the support is a huge, huge important thing and you can see it clearly.

I: Yeah, that's true. Well, good. Okay, I'm going to stop this because that's all the questions I have.
