# Supplementary material for: “Fluctuation is the norm”: Rehabilitation practitioner perspectives on ambiguity and uncertainty in their work with persons in disordered states of consciousness after traumatic brain injury
Source: PLoS One. 2022 Apr 21;17(4):e0267194. doi: 10.1371/journal.pone.0267194 (PMC9022828; doi:10.1371/journal.pone.0267194)
Supplement: Attachment — Submitted filename: Papadimitriou Response Letter3.pdf [file pone.0267194.s003.pdf]

October 30, 2021

Rebuttal letter for manuscript: “Fluctuation is the norm”

Dear Sara Rubinelli,

We thank you and reviewers for the careful review of our research manuscript: “Fluctuation is the norm:” Rehabilitation practitioner perspectives on ambiguity and uncertainty in their work with persons in disordered states of consciousness after traumatic brain injury. We have made all the changes requested by the editorial team and reviewers.

Eight editorial changes were requested.

1. We reviewed the PLOS ONE style requirements and adjusted our manuscript.
2. We now include tables as part of our main manuscript. There is one figure that we add as a separate file and as supporting information, as you requested.
3. Role of Funder: Funding for this project came from the Congressionally Directed Medical Research Programs (CDMRP) and Joint Warfighter Medical Research Programs (JWFMRP) of the United States Department of Defense. The funder had no role in the study design, data collection and analysis, decision to publish, or preparation of the manuscript. The specific award names and numbers are: CDMRP W81XWH-14-1-0568 and JWFMRP W81Xwh-16-2-0023.
4. We expand the acronym CDMRP: Congressionally Directed Medical Research Programs
5. We removed funding from acknowledgments. We have added the funding information in the Funding Statement section of the online submission form.
6. We have completed the competing interest online submission forms.
7. Data sharing statement: The data used in this manuscript are all qualitative interviews. De-identified data are provided to you in a single PDF file in the revised submission. We thank you for your support in making these available.
8. We include full ethics names of the IRB in the methods section: Northwestern University and Edward Hines, Jr. VA in IL approved the study and verbal consent: NU IRB: STU00203840 and Hines IRB: 16-037.

We address both reviewer comments and suggestions in our revised manuscript. Specifically, Reviewer #1 commented:

1. “Provide a more detailed definition of severe TBI and DoC.”

The introduction now includes information on sTBI and DOC with additional references.

“About 59% of persons who receive specialty rehabilitation will recover from DoC within the first year of recovery. For those remining in DoC recovery will continue for several years, but the odds of substantive recovery incrementally decreasing each year thereafter.

Recovery from DoC is described by a gradient of consciousness where less consciousness is associated with more disruption of functional and structural neural connectivity. While the gradient is delineated clinically as the vegetative state (VS), minimally conscious state (MCS), and emergence from MCS (eMCS) recovery, is not necessarily a linear progression along this gradient. Persons remaining in states of DoC, often experience fluctuating levels of wakefulness and external awareness and, even with highly specialized care, this inherent variability in neurobehavioral performance obscures clinical observations

of functioning during rehabilitation. This fluctuation challenges practitioners' day-to-day work because it is hard to unequivocally determine patients' level of consciousness. In this way, practitioners work in a context of scientific uncertainty regarding accurate detection of changes in levels and states of consciousness which is the basis for monitoring recovery. At the same time, there is a lack of empirical data to guide clinical treatment[9], which creates ambiguity about treatment decision-making. How practitioners perceive and make sense of diagnostic and prognostic uncertainty and therapeutic ambiguity remains an uncharted psychosocial domain and an unappreciated aspect of DoC rehabilitation treatment."

2. "The authors cite several articles from the ACRM DoC task force. Would it make sense to discuss this further in your paper?"

The ACRM DoC task force is a group of researchers and practitioners that volunteer their time to share with each other information about research and clinical projects around DoC. They often come together to draft guidelines and policy statements. We have cited these articles because they are, to our knowledge, the most current evidence-based, peer-reviewed scholarship on the subject. We believe we would be remiss if we failed to cite this scholarship.

3. "Table 1. The sample is mostly females. Should this be mentioned in the limitations or is this representative of the population?"

According to several reports, our sample is representative of the rehabilitation workforce. The American Speech-Language-Hearing Association (ASHA) reported in 2019, that 96% of the 175,000 SLPs certified by ASHA are female. (American Speech-Language-Hearing Association. (2019). A Demographic Snapshot of SLPs. The ASHA Leader, 24(7), 32.0)

The results of the 2017 National Nursing workforce survey, indicate that 9.1% of the 4,639,548 registered nurses are male, in other words 90.9% of the RN workforce in the United States identifies as female. (Smiley, R.A., Lauer, P., Bienemy, C., Berg, J., Shireman, E., Reneau, K.A., Alexander, M. (2018). The 2017 national nursing workforce survey. Journal of Nursing Regulation. 9(3), S1-S88.)

According to the American Occupational Therapy Association Workforce and Salary Survey of 2019, 91% of the OT workforce identifies as female. (American Occupational Therapy Association. (2021). 2019 Workforce & Salary Survey.)

Similarly, statistics published by the American Physical Therapy Association indicate that 65% of physical therapists and 71% of physical therapy assistants identify as female. (American Physical Therapy Association. (2020). APTA Physical Therapy Workforce Analysis.)

We have included this information in the manuscript when we describe our findings.

4. "Table all exemplar quotes by theme/ categories. This makes it easier to read and review."

We revised Table 2 to make it easier to read and review.

5. "Clarify if a qualitative software analysis program was used and if so, which one? NVIVO?"

We added the following sentence in our Methods section "We used the qualitative software NVIVO 11 to organize our data and work on codes and categories."

We now turn to address Reviewer #2 comments:

1. "The major drawback of the manuscript is the unclear conclusion, as the creative tinkering has been an age old technique used for the DoC and is the alternative way in the state of unambiquity and uncertainty by the medical practitioners dealing with DoC. The conclusion drawn here seems very obvious and known to the field."

We suspect Reviewer #2 made a typo of the work 'ambiguity,' we are not familiar with the word "unambiquity." In this statement, Reviewer #2 states that our conclusion is "unclear" and "very obvious and known to the field." We interpret this statement to mean that our conclusions are unclear vis a vis innovation in the field of severe traumatic brain injury (sTBI) and disorders of consciousness (DoC) in particular. We respectfully disagree with this statement. The fields of sTBI and DoC do not explicitly talk about tinkering as a creative way to practice in medical rehabilitation. It is not routinely or explicitly taught as a clinical tool in medical curricula. Very importantly, it is not well documented in the peer reviewed literature. We do appreciate, however, that rehabilitation practitioners may in fact be using this clinical reasoning tool to make sense of as well as treat persons in DoC. Indeed, this became clear to us in the data and it is why we chose to report on it in this paper. We believe that we need to make this creative tool more explicit in the field-- show what it looks like in practice, explain how practitioners think about it, and learn from them about how to use it and educate others about it. As such, this paper explores a clinical reasoning practice that fills a gap in the peer reviewed literature in the field of DoC after sTBI. By adding to the peer-reviewed literature, we hope to bring awareness to rehabilitation practitioners' clinical practices that are often forgotten.

We have added several sentences throughout the manuscript that make the intention of this paper more obvious to the reader.

Page 22: "…we show the value that practitioner clinical practices bring to the field of DoC and thus expose the epistemic injustice of treating "thinking in action" as inferior to EBM. We hope that future research and scholarship continue to explicate practitioners' experiences, practices and ways of working with patients in DoC as valuable ways of knowing and doing. Our data and research in clinical practice suggest that "thinking in action" and tinkering is one of the tools/ ways practitioners use in clinical practice. Yet, there is very little in the peer-reviewed literature about the value this practice can offer rehabilitation medicine."

Page 23: "Tinkering as a practice and as a way of caring, however, is not generally taught in educational curricula and it is not celebrated as a creative response to caring for complex patients. Tinkering is not perceived as important in the peer reviewed literature since there is a paucity of studies that explicate it as a practice, even though our practitioners clearly use it daily."

Page 24: "In searching for consciousness, practitioners breach the canon of EBM. In looking for consciousness, practitioners tinker with their treatment toolbox. As they tinker, they expose the limitations of the current state of scientific knowledge in the field of DoC. Tinkering, in this sense then, is a clinical reasoning practice that breaches the canon of rehabilitation science. As such, it has the potential to open the field of DoC practice to celebrating practitioners' ways of caring and treating. This may promote exploration and innovation of new treatment modalities and practices."

2. “The manuscript need to include some scientific reasoning behind the unambiguity and uncertainty associated with TBI.”

We suspect Reviewer #2 made a typo of the work ‘ambiguity,’ because we are not sure how the word “unambiguity” is related to what we are discussing. We have included additional references for this (in the introduction), per the request of the reviewer.

In addition to information added in the introduction to explain sTBi and DoC (per Reviewer #1 comments), we added the following on page 4 to respond to this comment:

“Rather than positioning this study as an examination of how practitioners’ clinical decision-making influences patient outcomes in order to inform quality improvement initiatives or advance person-centered care, we describe the more elementary practice of decision-making processes and underlying reasoning that enable monitoring of recovery and the inter-related treatment decisions. Doing so enables a more nuanced understanding of practitioners’ everyday clinical reasoning and the strategies they use to cope with diagnostic uncertainty and therapeutic ambiguity. This is turn can lead to new insights and innovation regarding clinical practice and knowledge in sTBI and DoC.”

3. “Again, it would be advantageous to include some scientific reasoning why tinkering methods could be helpful in regaining consciousness.”

To address this comment, we decided to explain better why the field of DoC is complex and replete with ambiguity and uncertainty (per reviewer #2, second concern) in order to contextualize the importance of understanding how practitioners respond to these complexities and challenges. In contextualizing the practice of tinkering in this way, we acknowledge practitioners’ creative commitment to their patience and clinical practice and engage in learning from them. They are, after all, the ones who interact with patients in order to support the regaining of consciousness. In this study, we didn’t set out to research whether tinkering practices are efficacious to regaining consciousness. We agree with Reviewer #2 that this is a very important question and it would be advantageous for the field to study this topic. Our data do not render themselves to answer this question at this moment. We argue that understanding deeply how practitioners work with patients and families in the field of DoC helps the field by making explicit clinical practices that could be helpful in medically complex context when there is scientific uncertainty regarding accurate detection of DoC states and epistemic ambiguity about treatment decision making which lead to diagnostic and prognostic uncertainty and inaccuracy. In these contexts, practitioners use tinkering to make sense of and cope with clinical complexity. By making explicit this clinical practice, we show that practitioners are “thinking in action” in ways that may be transgressive of the rehabilitation canon. We hope that by showing “thinking in action” we are supporting the practitioners of the field honor their work and actively learn from it.

To this end, we have added several sentences in the manuscript to remind the reader of these issues.

Page 26-7: “We hope future studies in the field of medical rehabilitation and DoC in particular will continue to make visible the creative ways that practitioners use to respond to epistemic uncertainty when they care for complex patients. Making visible how practitioners engage in tinkering practices is one way that we contribute to the field of rehabilitation and DoC. Whether tinkering is efficacious to supporting emergence to consciousness for patients in DoC remains unclear and an area of study that needs to be further explored.”

In response to Reviewer #2 comments, we heavily edited our concluding paragraph: "Rehabilitation practitioners who care for patients in DoC work in an environment of ambiguity and uncertainty. Ambiguity exists when there is either no evidence base or there is an imprecise scientific basis to guide diagnoses, prognostication and treatment decisions. Uncertainty occurs when there is high variability in patient responses to treatment and recovery patterns are unpredictable. EBM rehabilitation training curricula do not provide the tools to manage ambiguity, and the diagnostic and prognostic uncertainty of DoC challenges practitioners. The practitioners in our study responded to ambiguity and uncertainty by using their observation skills to monitor nuances in patients' responses that might indicate emerging consciousness. They did so by *searching for consistent* behavioral responses to stimuli as indications that the patient is improving, *observing* fine nuances, and *collaborating* with peers to grapple with unexplained recoveries or stalls in patient progress. While uncertainty raises discomfort, practitioners in our study used "thinking in action" tools such as tinkering to respond to uncertainty in order to care for their patients. They "tried things," used trial and error, worked "outside the box", tweaked things –they tinkered in order to provide optimal care. They sometimes admitted they didn't know why patients recovered the way they did. In admitting they didn't know, they showed their capacity for humility and vulnerability. Practitioners do not simply provide care to patients according to pre-established guidelines; they generate important knowledge by "thinking in action" and tinkering described in this paper. Understanding these practices can lead to new knowledge; practitioners' innovations can generate new insights that can move the science and practice of DoC forward. This study described the innovative ways rehabilitation practitioners deal with ambiguity and uncertainty in working with patients in DoC through tinkering, and as such, opens up the black box of rehabilitation practice. "

In closing our letter, we want to reiterate an important point about our findings that responds to the critique that our work may not be innovative or rigorous enough.

The work we present in this manuscript uses qualitative interviewing to understand rehabilitation practitioners' work in severe traumatic brain injury and disorders of consciousness. Interviewing practitioners is not novel. But using the methodology of narrative interviewing is uncommon in the field of rehabilitation medicine. This methodology enables the interviewee to share detailed events and stories from their experiences that allow us to gain in depth understanding and nuance that is not typically achieved in other forms of interviewing. We have outlined in the methods section our rigorous analytic process within our multi-disciplinary team which included multiple readings of the transcripts, iterative discussions, dialogical intersubjectivity, and audit trails. We have followed a rigorous study design and analysis process, and argue that judging qualitative interviews as lacking rigor is concerning since the research topic of our work required these methods.

Practitioners in DoC are well aware of the complexity of working in this field. Our findings uncovered practitioners' creative and committed responses to the ambiguity and uncertainty of their day to day work with patients. The fields of rehabilitation medicine, sTBI and DoC do not commonly study practitioners' work in the detail and nuance that we report in this manuscript. Typically, practitioners' work is seen as driven by tasks, understood within contexts of competencies and adherence to guidelines. Our work follows the seminal works of P. Benner in Nursing, C. Mattingly in occupational Therapy, and G. Jensen in Physical therapy, all of whom used qualitative designs include interviewing, to

understand practitioners' interactions with each other and with patients in order to explicate clinical practices that can be defined as expert, competent, emerging, and show how clinical reasoning occurs in practice. These works allow us to appreciate that practitioners use practices that may or may not be taught in the classroom, that they act in ways that are hard to communicate verbally but are observable, and that their work involves a thinking in action and a wisdom that are often not talked about, and not discussed in most textbooks. Our work adds to this scholarship. In the field of rehabilitation medicine and practice, this kind of scholarship is innovative. Our work has the potential to transform rehabilitation practice by making visible what rehabilitation practice really looks like for those working in DoC.

We thank the editorial team and reviewers for their careful and thoughtful suggestions on how to improve our manuscript. We look forward to your feedback.

Sincerely,

Christina Papadimitriou, PhD

Jennifer A Weaver, PhD, OTR/L,

Ann Guernon, PhD, CCC-SLP,

Elyse Walsh, PT, DPT, NCS, CCRC,

Trudy Mallinson, PhD, FACRM,

and Theresa L. Bender Pape, MA, CCC-SLP, Dr.PH., FACRM
