# Supplementary material for: “Fluctuation is the norm”: Rehabilitation practitioner perspectives on ambiguity and uncertainty in their work with persons in disordered states of consciousness after traumatic brain injury
Source: PLoS One. 2022 Apr 21;17(4):e0267194. doi: 10.1371/journal.pone.0267194 (PMC9022828; doi:10.1371/journal.pone.0267194)
Supplement: Attachment — Submitted filename: Letter to Editor Revision 3 Fluctuation is the norm.pdf [file pone.0267194.s005.pdf]

2/16/2022

Rebuttal letter for manuscript: “Fluctuation is the norm”

Dear Sara Rubinelli,

We thank you and reviewers for the careful review of our revised manuscript: “Fluctuation is the norm:” Rehabilitation practitioner perspectives on ambiguity and uncertainty in their work with persons in disordered states of consciousness after traumatic brain injury. We respond to your request for further information below and in the manuscript.

Reviewer #2 stated: “The authors of the manuscript (“Fluctuation is the norm”: Rehabilitation practitioner perspectives on ambiguity and uncertainty in their work with persons in disordered states of consciousness after traumatic brain injury”) have provided satisfactory answers to the questions. The authors are still advised to provide additional information for the following comments before the final submission.

1. Can there be any potential disadvantages of the tinkering? Please throw some light on the potential ways these practices can be implemented in a systematic manner and what are the potential limitations.”

We appreciate the inquiry about addressing disadvantages of tinkering as well as about potential ways to implement it.

In our paper we have stated that participants are challenged by the omnipresent uncertainty and ambiguity inherent in treating persons in DoC. We stated that “in searching for consciousness, practitioners breach the canon of EBM… [and] tinker with their treatment toolbox. As they tinker, they expose the limitations of the current state of scientific knowledge in the field of DoC. Tinkering, in this sense then, is a clinical reasoning practice that breaches the canon of rehabilitation science.” While breaching the cannon of EBM may provide an opportunity to promote innovation of new treatment modalities and practices, explicit use of tinkering as a form of clinical reasoning may expose patients to unnecessary risk, or at the very least, expose patients to ineffective interventions. We do not believe the state of the science of tinkering is such that it could be recommended for implementation in a “systematic manner.” Future research is needed to understand the role of tinkering in tailoring interventions to patients, perhaps by balancing EBM training with ethical clinical practice.

We have included this additional information in the *Implications for Rehabilitation* section of our paper (pp. 31-2, lines 550-556). We hope it addresses the comments of reviewer #2 sufficiently.

We reviewed the whole manuscript again and noticed a few typos which we corrected, and a repeated sentence on lines 534-5 which we have now deleted.

Thank you for the opportunity to revise our manuscript and for your thorough review process.
Sincerely,

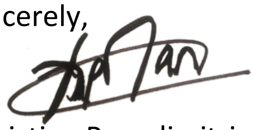

Christina Papadimitriou, PhD (corresponding author)

Jennifer A Weaver, PhD, OTR/L,

Ann Guernon, PhD, CCC-SLP,

Elyse Walsh, PT, DPT, NCS, CCRC,

Trudy Mallinson, PhD, FACRM,

and Theresa L. Bender Pape, MA, CCC-SLP, Dr.PH., FACRM
