# Supplementary material for: “Fluctuation is the norm”: Rehabilitation practitioner perspectives on ambiguity and uncertainty in their work with persons in disordered states of consciousness after traumatic brain injury
Source: PLoS One. 2022 Apr 21;17(4):e0267194. doi: 10.1371/journal.pone.0267194 (PMC9022828; doi:10.1371/journal.pone.0267194)
Supplement: Attachment — Submitted filename: Plos one_Fluctuations is the norm_Revison_HV.doc [file pone.0267194.s002.doc]

The manuscript under review, **“Fluctuation is the norm”: Rehabilitation practitioner perspectives on ambiguity and uncertainty in their work with persons in disordered states of consciousness after traumatic brain injury** addresses the issues related to uncertainty and unambiguity associated with the rehabilitation science for the improvement of patients in disordered states of consciousness (DoC). The authors have specifically illustrated the importance and limitations of the canonical and scientific procedures adopted by medical practitioners and distinctively provided the importance of alternative tinkering and the canonical breaching methodologies adopted by the practitioners dealing with DoC. Here authors have emphasized that the interpretation and narration of the practitioner’s specific strategy could be his own way of understanding and interpretation. They have addressed this as a major concern for the rehabilitation science dealing with DoC. Overall the manuscript is written in a well narrative manner, but as per my view it lacks the suitability with the journal’s goal, scientific novelty and rigor and is more relevant for the psychological sciences.

Here are the major comments:

1. The major drawback of the manuscript is the unclear conclusion, as the creative tinkering has been an age old technique used for the DoC and is the alternative way in the state of unambiquity and uncertainty by the medical practitioners dealing with DoC. The conclusion drawn here seems very obvious and known to the field.
2. The manuscript need to include some scientific reasoning behind the unambiguity and uncertainty associated with TBI.
3. Again, it would be advantageous to include some scientific reasoning why tinkering methods could be helpful in regaining consciousness.
