# Supplementary material for: “Fluctuation is the norm”: Rehabilitation practitioner perspectives on ambiguity and uncertainty in their work with persons in disordered states of consciousness after traumatic brain injury
Source: PLoS One. 2022 Apr 21;17(4):e0267194. doi: 10.1371/journal.pone.0267194 (PMC9022828; doi:10.1371/journal.pone.0267194)
Supplement: Attachment — Submitted filename: Fluctuation is the norm_Reviewer 2 comments_v2.docx [file pone.0267194.s004.docx]

The authors of the manuscript (**“Fluctuation is the norm”: Rehabilitation practitioner perspectives on ambiguity and uncertainty in their work with persons in disordered states of consciousness after traumatic brain injury”)** have provided satisfactory answers to the questions. The authors are still advised to provide additional information for the following comments before the final submission.

1. Can there be any potential disadvantages of the tinkering? Please throw some light on the potential ways these practices can be implemented in a systematic manner and what are the potential limitations.
